# The Missing Element in the World of Macrocycles—*p*‑Pyriporphyrin

**DOI:** 10.1021/acs.orglett.6c01338

**Published:** 2026-05-05

**Authors:** Julia Kamyszek, Radomir Myśliborski, Daria Modelska, Paulina Krzyszowska, Michał J. Białek, Marta Gordel-Wójcik, Piotr J. Chmielewski, Lechosław Latos-Grażyński, Karolina Hurej

**Affiliations:** Department of Chemistry, 49572 University of Wroclaw, F. Joliot-Curie 14, 50383 Wroclaw, Poland

## Abstract

The missing element of the pyriporphyrin macrocycle library was synthesized and investigated. The [3 + 1] synthesis enabled the production of 24-thia- and 24-tellura-*para*-pyriporphyrin. The resulting macrocycles exhibit interesting conformational dynamics, analyzed using nuclear magnetic resonance (NMR) and circular dichroism (CD) spectroscopy. Further studies revealed that they act as ligands, coordinating to metal ions, including palladium­(II) and ruthenium­(II). Additionally, thiophene-containing derivatives and their ruthenium complexes exhibit photoluminescence properties. The palladium complexes of both macrocycles catalyze the dealkylation of tertiary amines.

## Graphical abstract

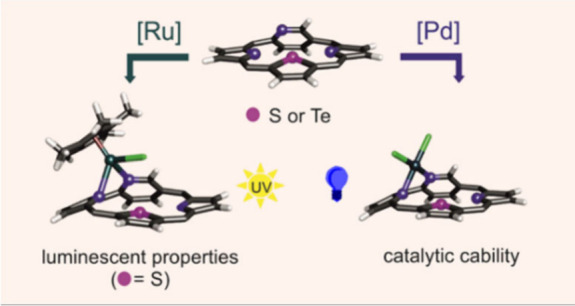

Incorporating a pyridine ring into the macrocycle skeleton results in the compounds exhibiting very different reactivity from regular porphyrins.^1–6^ In most pyriporphyrins, until now, the six-membered ring has been incorporated into positions α and α′ or γ and γ′.^1,7,8^ In 2018, we synthesized an expanded, acid-controlled, conformational-inversion-based pyridine macrocycle **1** that exhibits a photoacoustic response upon palladium­(II) coordination.^9^

Pyriporphyrins are analogues of benziporphyrins, which belong to the broader class of carbaporphyrins (Figure 1). The structural difference lies in the macrocyclic core, where at least one of the inner nitrogen atoms of a classical porphyrin is replaced by a carbon atom.^10–12^ The propensity of compound **2** to coordinate metal ions through diverse binding types, including η^2^-type interactions, as well as its ability to undergo a six-membered ring contraction leading to cyclopentadiene formation, suggests that its pyridine analogue may likewise display distinctive and potentially versatile coordination behavior.^13–17^

**Figure 1:**
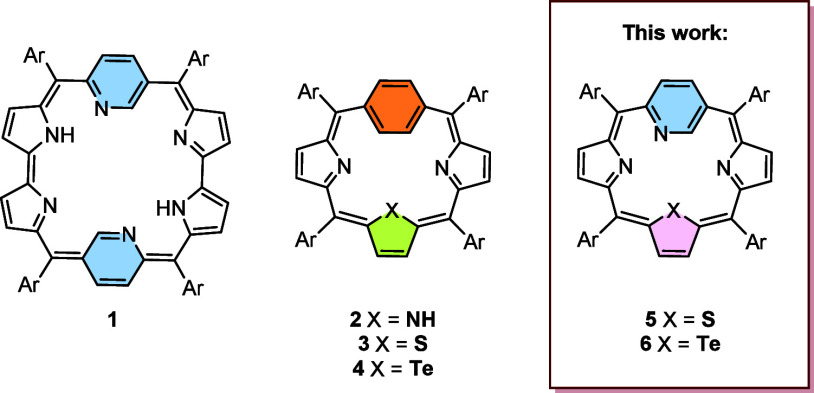
Di-*para*-pyrirubyrin **1**, 24-hetero-*para*-benziporphyrins **2**–**4**, and 24-hetero-*para*-pyriporphyrins **5** and **6**.

Heterocarbaporphyrins featuring a [CXNN] coordination cavity represent structurally related systems that exhibit comparable metal-binding characteristics.^18–22^ In 2019, Ravikanth and co-workers reported heteroanalogues of *p*-benziporphyrin incorporating thiophene (**3**), selenophene, or tellurophene (**4**) rings.^23^ These macrocycles were synthesized using a [3 + 1] condensation strategy, albeit in modest yields (4–6%). Furthermore, their coordination chemistry toward metal ions was investigated. Notably, studies on tellurium-containing derivative **4** revealed that palladium­(II) coordinates to both tellurium and nitrogen donor atoms.^23^

We have obtained 24-hetero-*p*-pyriporphyrins **5** and **6** based on a pathway similar to that of the di-*p*-pyriporphyrin precursors described earlier (Scheme 1).^24^

**Scheme 1:**
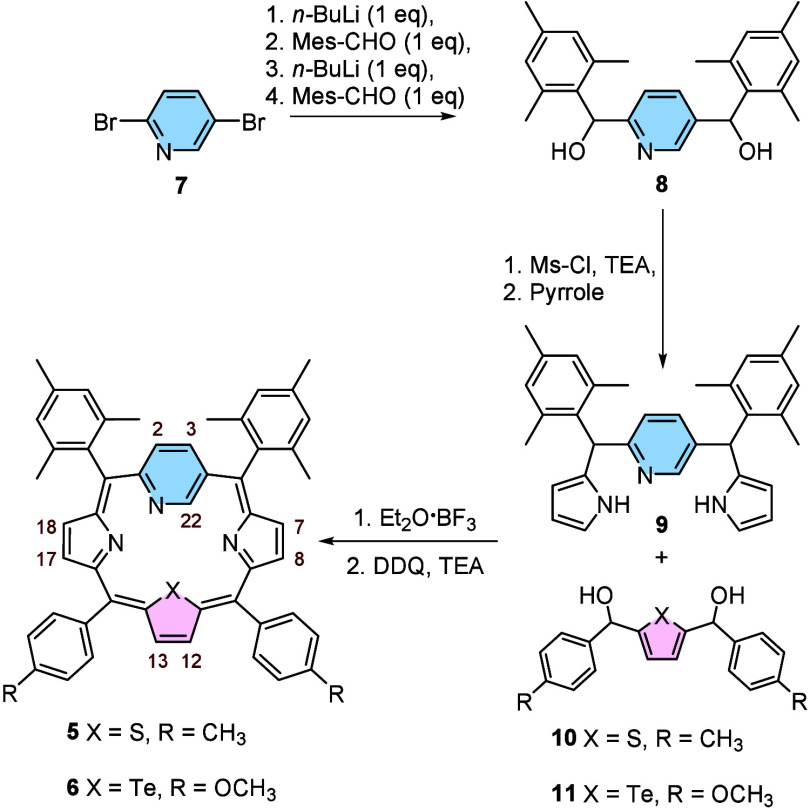
Synthetic Pathway for Obtaining 24-Hetero-*para*-pyriporphyrins **5** and **6**

The reaction of *p*-pyritripyrrane **9** with the proper heterodiol, **10** or **11**, catalyzed by Et_2_O• BF_3_ and oxidized by DDQ, produced aromatic macrocyclic compounds with yields of 25 and 21%, respectively. The compositions of both macrocycles were confirmed by HRMS analysis.

The resulting macrocycles, 24-thia-*p*-pyriporphyrin (**5**) and 24-tellura-*p*-pyriporphyrin (**6**), exhibit similar ^1^H NMR spectra (Figure 2), but the aromatic ring current effect was slightly more pronounced for compound **5**. Although the heterocyclic ring proton signals of compounds **5** and **6** differ only slightly (δ 8.49 vs 8.39 ppm, respectively), a pronounced difference is observed for the inner CH(22) resonance, which appears at 4.68 ppm for **5** and 6.45 ppm for **6** (Figure 2). A similar effect was observed in the case of 24-hetero-*p*-benziporphyrins, where the proton resonances of the six-membered ring were shifted toward lower chemical shifts, with the magnitude of the shift depending on the nature of the incorporated heterocycle.^23^ This result is primarily due to the less planar structure of macrocycle **6**. The deviation of the α,β-pyridine moiety, in the main conformation, with respect to the plane defined by four *meso*-carbon atoms is 39° for compound **5**. For macrocycle **6**, the deviation is 46° (Figure S53). The positions of the C(22) signals at 156 ppm (for **5**) and 159 ppm (for **6**) are notable in the ^13^C NMR spectrum, as this is a shift specific for α-pyridine carbon atoms.^23^

**Figure 2:**
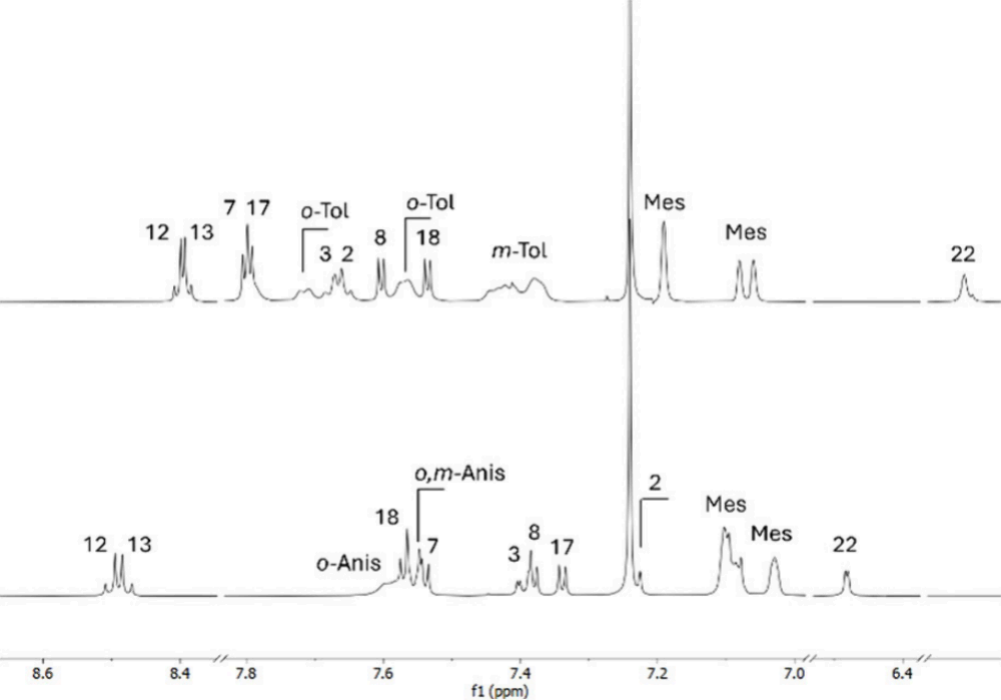
Selected regions of the ^1^H NMR spectra of **5** (top) and **6** (bottom) (600 MHz, CDCl_3_, 300 K).

Tetraphenyl-*p*-benziporphyrin rapidly interconverts between two equivalent conformations that differ only in the orientation of the *p*-phenylene moiety relative to the plane defined by the four *meso*-carbon atoms. This librational motion freezes below 168 K in solution.^12^ A more complex situation is observed for 24-hetero-*p*-pyriporphyrins **5** and **6** (Figure 3). Incorporation of a nitrogen atom into the six-membered ring of 24-hetero-*p*-benziporphyrins leads to the formation of two enantiomeric pairs: (1) **5**_in,down_ and **5**_in,up_ and (2) **5**_out,down_ and **5**_out,up_, as shown in Figure 4. Variable-temperature ^1^H NMR spectra recorded in several solvents, including dichloromethane-*d*_2_, benzene-*d*_6_, toluene-*d*_8_, and pyridine-*d*_5_, indicate that the solvent strongly influences the preferred conformations (see Figures S3–S6). The complexity of the ^1^H NMR spectra reflects both the conformational exchange process and the thermodynamic equilibrium between conformers (see the Supporting Information).

**Figure 3:**
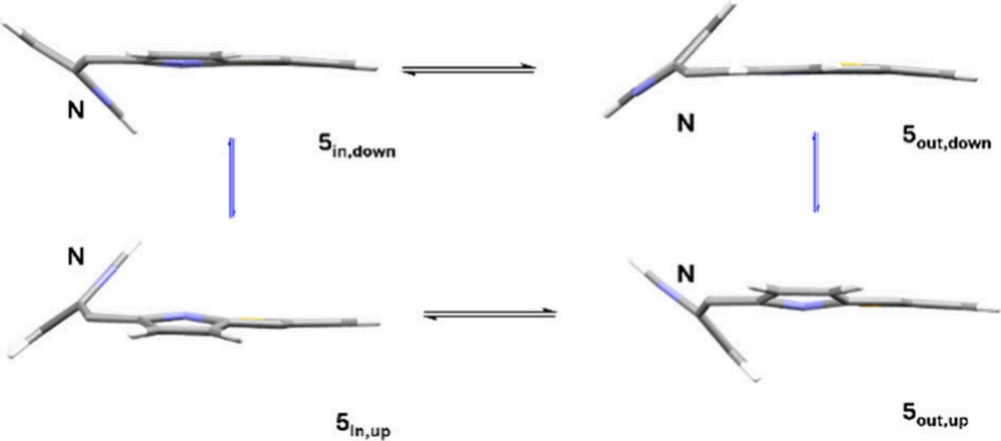
DFT-optimized models of conformers of **5**. Black arrows, changes observable in ^1^H NMR; blue arrows, changes that cannot be observed by ^1^H NMR but can be detected using CD spectroscopy. For clarity, *meso*-aryl groups were omitted.

**Figure 4:**
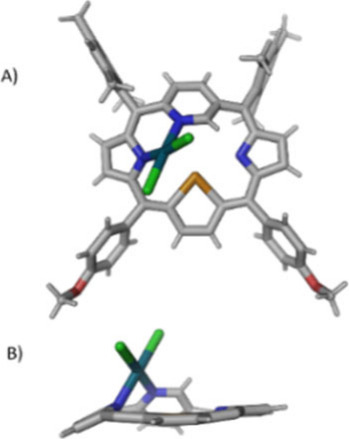
X-ray crystal structure of **13**, showing (A) perspective view of **13** and (B) side view (aryl groups omitted for clarity).

The ^1^H NMR spectra of **5** were measured in dichloromethane-*d*_2_ over the entire accessible temperature range, indicating that both conformers are in exchange (Figure 2). In toluene-*d*_8_, however, at temperatures below 200 K, enantiomers **5**_in,down_ and **5**_in,up_ become predominant. Although the exchange process slows down, the second pair, **5**_out,down_ and **5**_out,up_, cannot be directly observed due to severe line broadening. Upon heating the sample in toluene-*d*_8_, benzene-*d*_6_, or pyridine-*d*_5_, the pyridine proton resonances gradually converge (see Figures S4–S6). At temperatures above 350 K, an averaged spectrum is observed, indicating nearly equal populations of the two conformers. Such ring rotations are typical of this type of dynamic structure and have also been observed in 1,4-naphthiporphyrin^25^ and in expanded derivatives with an embedded *para*-phenylene moiety.^26^

The racemization process, that is, inversion of the pyridine moiety through the interior of the macrocycle, is very slow on the ^1^H NMR time scale. This is evidenced by the persistent observation of four *meta*-H and four *ortho*-CH_3_ resonances. However, CD experiments following HPLC separation of the principal enantiomers revealed that racemization occurs with an enantiomeric half-life of approximately 60 s (see Figure S73).

Recently, the presence of a biradical character has been reported for di-*p*-benziporphyrins.^27^ EPR measurements of 24-hetero-*p*-pyriporphyrins **5** and **6** confirmed the presence of thermally accessible triplet states (see Figure S54), although the contribution of the diradical form does not exceed a few percent (see Figure S65). The energy gap between the states is approximately 1.0 kcal mol^–1^ for **5** and 2.4 kcal mol^–1^ for **6**.

In di-*p*-pyrirubyrin **1**, the expected ring rotation was not observed. This is probably because the pyridine position is stabilized through N(25)–H···N(21) hydrogen bonding.^24^ A similar behavior was observed during the titration of **5** with acid. The only monocationic product formed is conformer **I** due to the stabilization of the six-membered ring position by a N(25)–H···N(21) interaction (Scheme 2). In general, titration of 24-thia-*p*-pyriporphyrin with tetrafluoroboric acid resulted in the stepwise protonation of both nitrogen atoms in the pyrrole rings. No trication was observed. These changes were monitored using NMR and UV–vis spectroscopy (see Figures S69 and S70). A similar effect was observed for 24-tellura-*p*-pyriporphyrin (Figure S71).

**Scheme 2:**
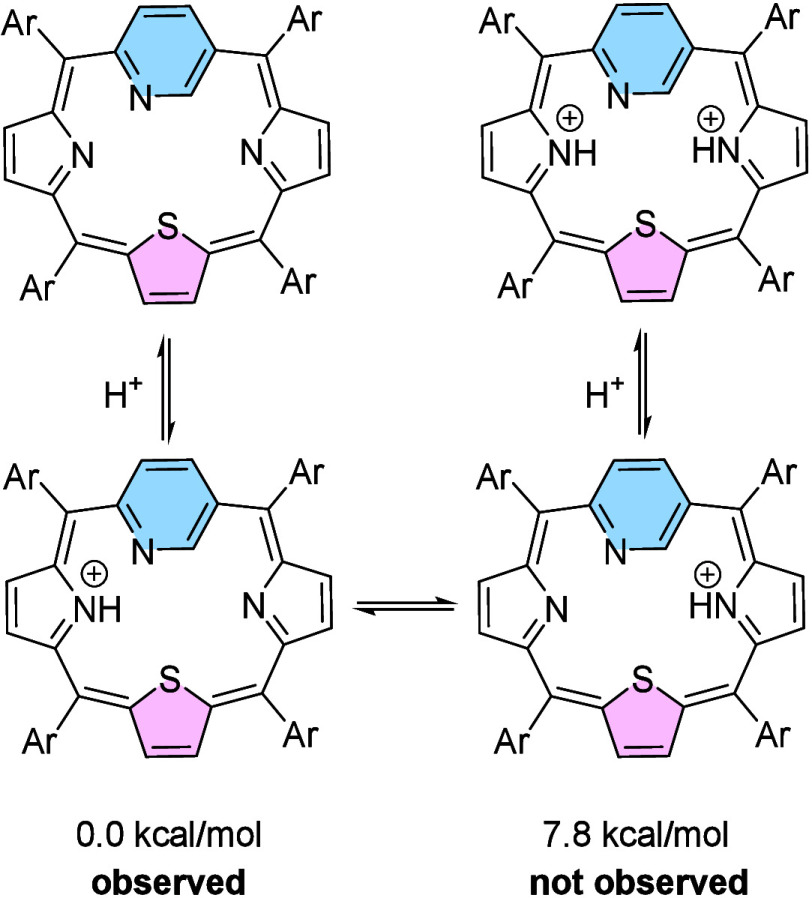
Protonation of **5** with HBF_4_, with Relative Energies of DFT-Optimized Models

The study of the coordination chemistry of compounds **5** and **6** began with experiments involving palladium­(II) ion insertion (Scheme 3). We obtained side-on complexes **12** and **13**. In both cases, the presence of a palladium ion was confirmed by mass spectroscopy (see Figures S49 and S50). Additionally, a single crystal of complex **13** was obtained and analyzed by using X-ray diffraction (Figure 4).

**Scheme 3:**
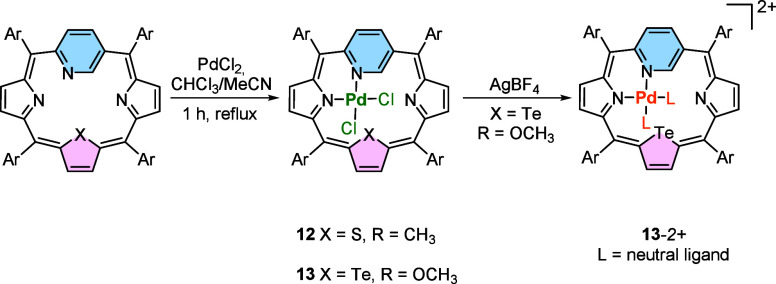
Synthetic Pathway of **12**, **13**, and **13-2+**

The Pd^2+^ ion adopts a square-planar geometry, binding to the nitrogen atoms of pyridine and pyrrole (Figure 4). Two other sites are occupied by chlorides that balance the charge. The binding of pyridine locks it at a 41.6° angle relative to the four *meso* mean plane. Another palladium coordination effect is the forced apical interaction between palladium and tellurium atoms from the five-membered ring. The distance between these atoms is 3.165(11) Å, whereas the sum of their van der Waals radii is 3.69 Å.

Density functional theory (DFT) studies for **12** (see Figure S55) confirmed the formation of an analogous complex for derivative **5**, similar to that observed for 24-tellura-*p*-pyriporphyrin **13**.

The ^1^H NMR spectra of complexes **12** and **13** confirmed changes in the chemical shifts of signals in comparison to the initial macrocycles **5** and **6** due to metal ion coordination (Figure 5). The proton signals of both the thiophene and tellurophene rings shifted to a higher frequency area (**12**, 9.17 ppm; **13**, 9.28 ppm), while proton CH(22) moves in the opposite direction (**12**, 3.73 ppm; **13**, 5.39 ppm). These changes indicate a greater influence of the aromatic ring current. The AMX spin system of the pyridine ring protons in both compounds is retained (Figure 5), which confirms that the C–H bond is not activated.

**Figure 5:**
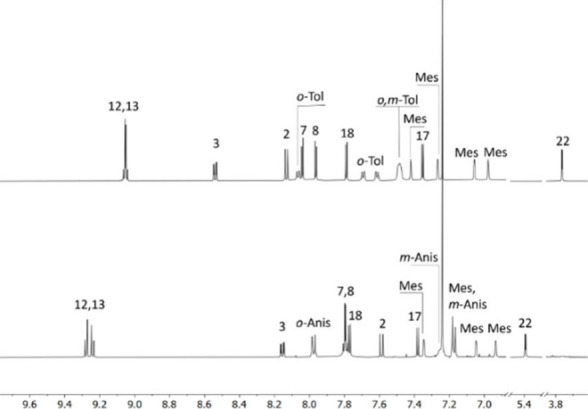
Selected regions of the ^1^H NMR spectra of **12** (top) and **13** (bottom) (600 MHz, CDCl_3_, 300 K).

The reactivity of complex **13** was further investigated in the presence of silver­(I) tetrafluoroborate (Scheme 3). The silver salt was added stepwise by titration, and the progress was monitored by ^1^H NMR spectroscopy (see Figures S38–S42). The results imply that chloride atoms were removed from the complex, thereby transforming it into a dicationic form, **13-2+**.

The addition of a base, such as triethylamine, revealed the catalytic potential of compounds **12** and **13**. Introducing triethylamine to **12** or **13** led to dealkylation of tertiary amines, yielding secondary amines and the corresponding aldehydes (Scheme 4; see the Supporting Information). Rhodium complexes (including porphyrin complexes) are often used as catalysts in this process.^29^ Although the overall efficiency did not exceed 50% under these conditions, we believe that further research will confirm the complexes’ high catalytic potential.

**Scheme 4:**
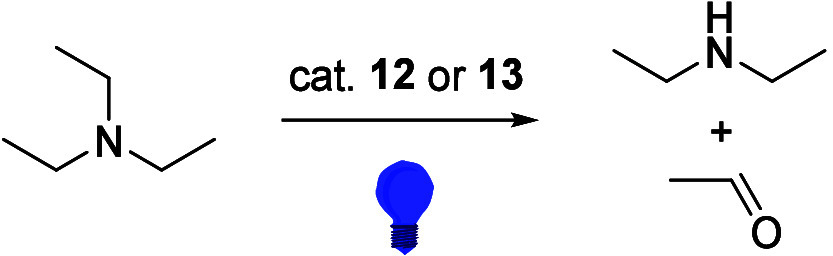
TEA Dealkylation in the Presence of Palladium Complexes **12** or **13** and Light

Next, we studied the coordination chemistry of ligands **5** and **6** with ruthenium­(II) ions (Scheme 5). Both reactions required anaerobic conditions and were carried out in a glovebox at room temperature for 24 h. The efficiencies of both reactions exceeded 90%. The cationic form of ligand **5** or **6** is the main product when a similar reaction is conducted in the air or at a higher temperature.

**Scheme 5:**
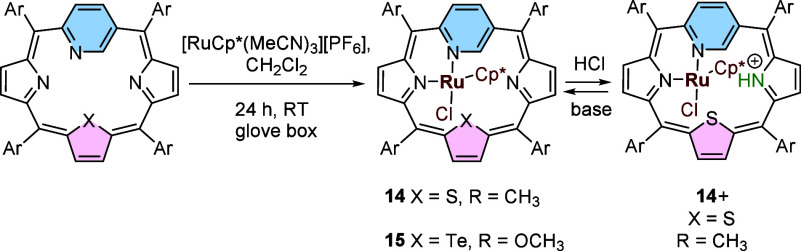
Synthetic Pathway for **14**, **15**, and **14+**

The ^1^H NMR spectra of complexes **14** and **15** are similar to those of their palladium analogues (**12** and **13**; Figure 6). However, the chemical shifts of the CH(22) protons are noteworthy, with values of 5.31 ppm for **14** and 6.38 ppm for **15**. These protons resonate in a higher frequency region, closer to that of free bases **5** and **6** than palladium complexes **12** and **13**. One explanation could be interactions with chloride anions, which are reasonably well-reproduced in DFT-optimized models (Figure 7).

**Figure 6:**
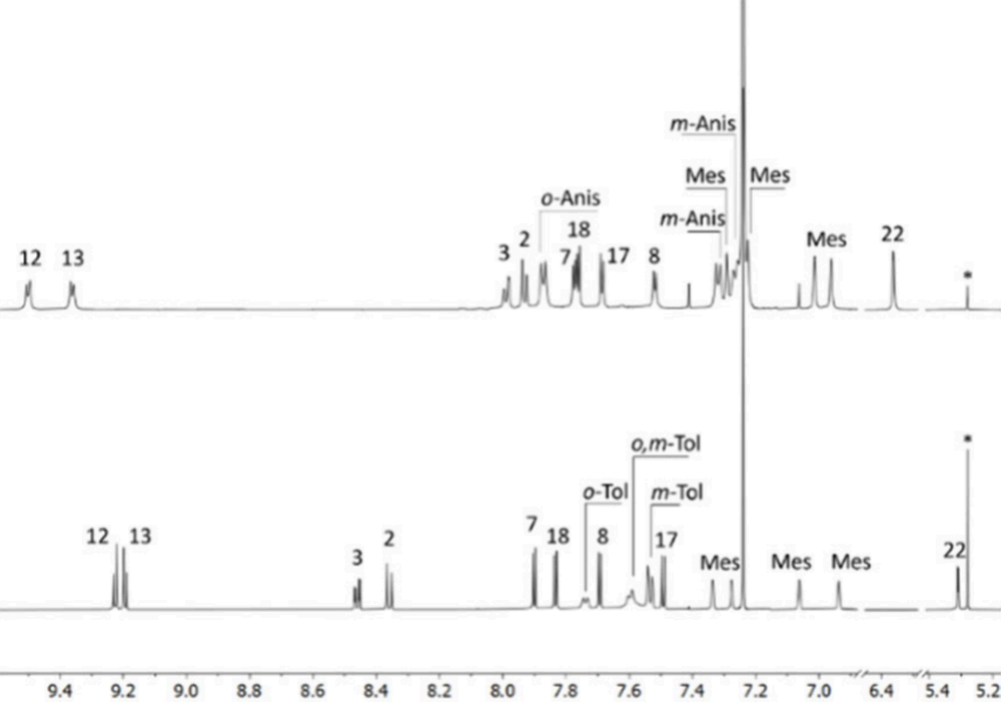
Selected regions of the ^1^H NMR spectra of **14** (bottom) and **15** (top) (600 MHz, CDCl_3_, 300 K).

**Figure 7:**
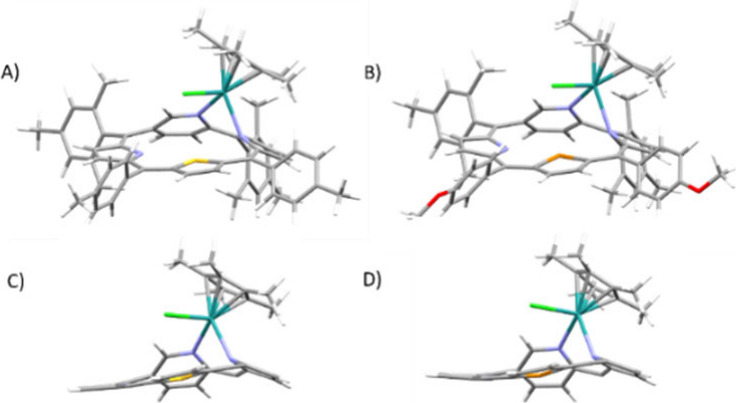
DFT-optimized models of (A and C) **14** and (B and D) **15**. For clarity, aryl groups were omitted from the side views (C and D).

Thus, the Ru^II^ ion in **14** and **15** coordinates two nitrogen atoms, one chloride ion, and a pentamethylcyclopentadienyl ring in η^5^ fashion (Figure 7). This is similar to the geometry observed in complexes with the *p*-cymene unit.^30^ The presence of the pentamethylcyclopentadiene ring is evidenced by signals at 0.35 ppm for **5** and 0.54 ppm for **6**, with an intensity of 15H, which originate from the Cp* methyl groups. The ^13^C NMR spectrum of compound **14** shows characteristic signals at 79 and 8 ppm, providing further confirmation that the Cp* motif has been preserved (Figure S20).

The addition of the base, such as triethylamine, does not affect compounds **14** and **15**. However, even a small amount of acid added to compound **14** converts it into its cationic form, **14+** (Scheme 5).

The electronic spectra were measured to provide a comprehensive characterization of the newly obtained macrocycles (Figure S74). All compounds exhibit features characteristic of aromatic porphyrins and carbaporphyrins.^31,32^ Emission spectra were observed only for 24-thia-*p*-pyriporphyrin **5**, its cationic form **5+**, and ruthenium complex **14**.

The emission spectra exhibit photoluminescence with a Stokes shift (Figure 8). The established quantum yields (QYs) (**5**, 7.3%; **5+**, 5.0%; and **14**, 4.9%) indicate slightly different emission efficiencies. The lifetimes recorded for these three molecules (**5**, 1.22 ns; **5+**, 1.21 ns; and **14**, 1.21 ns) are very similar and a bit shorter than those of regular porphyrins.^32,33^

**Figure 8:**
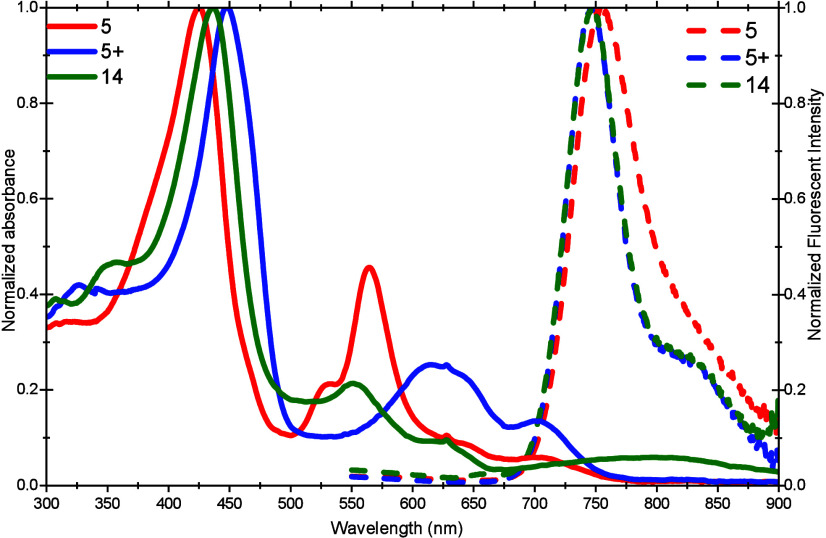
Normalized electronic absorption spectra (solid line) together with normalized fluorescence emission spectra (λ_ex_ = 450 nm, dotted line) of compounds **5**, **5+**, and **14** in dichloromethane.

In summary, 24-hetero-*p*-pyriporphyrins were synthesized using appropriate thiophene- or tellurophene-based diols and the tripyrrane precursors, containing pyridine substituted in α,β′ positions. Side-on complexes with palladium­(II) and ruthenium­(II) ions were obtained for both macrocycles. Palladium­(II) complexes catalyze the dealkylation reaction of tertiary amines, such as triethylamine. Furthermore, 24-thia-*p*-pyriporphyrin, its cationic form, as well as the ruthenium­(II) complex exhibit photoluminescent properties.

## Supplementary Material



## Data Availability

The data underlying this study are available in the published article and in its Supporting Information and openly available in Zenodo at 10.5281/zenodo.19221288.

## Notes

General comments and procedures, analytical data, and HRMS, UV–vis, and NMR spectra (PDF)
