# Supplementary material for: The Missing Element in the World of Macrocycles—p‑Pyriporphyrin
Source: Org Lett. 2026 May 5;28(19):6060–5. doi: 10.1021/acs.orglett.6c01338 (PMC13299050; doi:10.1021/acs.orglett.6c01338)
Supplement: Supplementary file 1 [file ol6c01338_si_001.pdf]

Supporting Information

The Missing Element in the World of Macrocycles – *p*-Pyriporphyrin

Julia Kamyszek, Radomir Myśliborski, Daria Modelska, Paulina Krzyszowska, Michał J. Białek, Marta Gordel-Wójcik, Piotr J. Chmielewski, Lechosław Latos-Grażyński and Karolina Hurej*

Table of contents

Table of contents 2

Methods 6

Experimental 8

Figures 13

NMR 13

Figure S1. Signals assignment - part of the ^{1}H NMR spectrum of 5 (top; 600 MHz, 300 K, $CDCl_3$); The ^{1}H NMR spectrum of 5 (bottom; 600 MHz, 300 K, $CDCl_3$). 13

Figure S2. The ^{13}C NMR spectrum of 5 (600 MHz, 300 K, $CDCl_3$). 14

Figure S3. Part of the ^{1}H NMR spectrum of 5 at temperatures ranging from 300 K to 170 K, with a 10 K interval between each temperature (600 MHz, VT, CD_2Cl_2). 14

Figure S4. Part of the ^{1}H NMR spectrum of 5 at temperatures ranging from 340 K to 180 K, with a 20 K interval between each temperature (600 MHz, VT, Tol-d_8). Stacked angle: 5%. 15

Figure S5. Part of the ^{1}H NMR spectrum of 5 at temperatures ranging from 300 K to 350 K, with a 10 K interval between each temperature (600 MHz, VT, C_6D_6). Stacked angle: 5%. 15

Figure S6. Part of the ^{1}H NMR spectrum of 5 at temperatures ranging from 300 K to 370 K, with a 10 K interval between each temperature (600 MHz, VT, Py-d_5). Stacked angle: 5%. 16

Figure S7. Changes in the position of the signal originating from the CH(22) group in the ^{1}H NMR spectrum as a function of temperature (top: *Tol-d_8*, VT, 600 MHz, middle: *Tol-d_8*, VT, 600 MHz, bottom: *CD_2Cl_2*, VT, 600 MHz). 17

Figure S8. Part of the COSY spectrum of 5 (600 MHz, 300 K, $CDCl_3$). 18

Figure S9. Part of the COSY spectrum of 5 (600 MHz, 300 K, $CDCl_3$). 18

Figure S10. Part of the NOESY spectrum of 5 (600 MHz, 300 K, $CDCl_3$). 19

Figure S11. Part of the HSQC spectrum of 5 (600 MHz, 300 K, $CDCl_3$). 19

Figure S12. Part of the HMBC spectrum of 5 (600 MHz, 300 K, $CDCl_3$). 20

Figure S13. Signals assignment - part of the ^{1}H NMR spectrum of 12 (top, 600 MHz, 300 K, $CDCl_3$), the ^{1}H NMR spectrum of 12 (bottom, 600 MHz, 300 K, $CDCl_3$) 21

Figure S14. The ^{13}C NMR spectrum of 12 (600 MHz, 300 K, $CDCl_3$). 22

Figure S15. Part of the COSY spectrum of 12 (600 MHz, 300 K, $CDCl_3$). 22

Figure S16. Part of the HSQC spectrum of 12 (600 MHz, 300 K, $CDCl_3$). 23

Figure S17. Part of the NOESY spectrum of 12 (600 MHz, 300 K, $CDCl_3$). 23

Figure S18. Part of the HMBC spectrum of 12 (600 MHz, 300 K, $CDCl_3$). 24

Figure S19. Signals assignment - part of the ^{1}H NMR spectrum of 14 (top; 600 MHz, 300 K, $CDCl_3$), the ^{1}H NMR spectrum of 14 (bottom; 600 MHz, 300 K, $CDCl_3$) 25

Figure S20. The ^{13}C NMR spectrum of 14 (600 MHz, 300 K, $CDCl_3$). 26

Figure S21. Part of the COSY spectrum of 14 (600 MHz, 300 K, $CDCl_3$). 26

Figure S22. Part of the NOESY spectrum of 14 (600 MHz, 300 K, $CDCl_3$). 27
Figure S23. Part of the HSQC spectrum of 14 (600 MHz, 300 K, $CDCl_3$). 27
Figure S24. Part of the HMBC spectrum of 14 (600 MHz, 300 K, $CDCl_3$). 28
Figure S25. Signals assignment - part of the ^{1}H NMR spectrum of 6 (top; 600 MHz, 300 K, $CDCl_3$); the ^{1}H NMR spectrum of 6 (bottom; 600 MHz, 300 K, $CDCl_3$) 29
Figure S26.The ^{13}C NMR spectrum of 6 (600 MHz, 300 K, $CDCl_3$). 29
Figure S27. Part of COSY spectrum of 6 (600 MHz, 300 K, $CDCl_3$). 30
Figure S28. Part of COSY spectrum of 6 (600 MHz, 300 K, $CDCl_3$). 30
Figure S29. Part of the HSQC spectrum of 6 (600 MHz, 300 K, $CDCl_3$). 31
Figure S30. Part of the NOESY spectrum of 6 (600 MHz, 300 K, $CDCl_3$). 31
Figure S31. Part of the HMBC spectrum of 6 (600 MHz, 300 K, $CDCl_3$). 32
Figure S32. Signals assignment - part of the ^{1}H NMR spectrum of 13 (top; 600 MHz, 300 K, $CDCl_3$), the ^{1}H NMR spectrum of 13 (bottom; 600 MHz, 300 K, $CDCl_3$)....................33
Figure S33. The ^{13}C NMR spectrum of 13 (600 MHz, 300 K, $CDCl_3$). 34
Figure S34. Part of the COSY spectrum of 13 (600 MHz, 300 K, $CDCl_3$). 34
Figure S35. Part of the COSY spectrum of 13 (600 MHz, 300 K, $CDCl_3$). 35
Figure S36. Part of the HSQC spectrum of 13 (600 MHz, 300 K, CDCl3). 35
Figure S37. Part of the HMBC spectrum of 13 (600 MHz, 300 K, $CDCl_3$). 36
Figure S38. Part of the COSY spectrum of 13-2+ (600 MHz, 250 K, $CDCl_3$). 37
Figure S39. Part of the COSY spectrum of 13-2+ (600 MHz, 250 K, $CDCl_3$). 37
Figure S40. Part of the HSQC spectrum of 13-2+ (600 MHz, 250 K, $CDCl_3$). 38
Figure S41. Part of the NOESY spectrum of 13-2+ (600 MHz, 250 K, $CDCl_3$). 38
Figure S42. Part of the HMBC spectrum of 13-2+ (600 MHz, 250 K, $CDCl_3$). 39
Figure S43. Signals assignment - part of the ^{1}H NMR spectrum of 15 (top; 600 MHz, 300 K, $CDCl_3$), the ^{1}H NMR spectrum of 15 (bottom; 600 MHz, 300 K, $CDCl_3$)....................40
Figure S44. Part of the COSY spectrum of 15 (600 MHz, 300 K, $CDCl_3$). 41
Figure S45. Part of the HSQC spectrum of 15 (600 MHz, 300 K, $CDCl_3$). 41
Figure S46. Part of the HMBC spectrum of 15 (600 MHz, 300 K, $CDCl_3$). 42
MS 42
Figure S47. Selected region of the HRMS ESI (+MS) spectrum of 5. 42
Figure S48. Selected region of the HRMS ESI (+MS) spectrum of 6. 43
Figure S49. Selected region of the HRMS ESI (+MS) spectrum of 12. 43
Figure S50. Selected region of the HRMS ESI (+MS) spectrum of 13. 44
Figure S51. Selected region of the HRMS ESI (+MS) spectrum of 14. 44
Figure S52. Selected region of the HRMS ESI (+MS) spectrum of 15. 45
DFT 46
Figure S53. The DFT-optimised models of 5 (left) and 6 (right). For clarity, aryl groups were omitted from the side views (bottom). 46

Figure S54. Spin density distribution for an optimized triplet model. Isovalue = 0.002.....46

Figure S55. The geometries and relative energies of the three possible coordination modes of the side-on palladium(II) complex with 24-thia-*p*-pyriporphyrin were obtained by DFT optimisation. Aryl groups omitted for clarity. 47

Figure S56. The DFT-optimized models (B3LYP 6-31G(d,p)/LANL2DZ(Te, Ru)) of 14 (A and C) and 15 (B and D). For clarity, aryl groups were omitted from the side views (C and D). 47

Investigate the effect of metalation on π-conjugation and ring current within macrocycles 5 and 12. 47

Figure S57. Electron density isosurface of A) and B) different conformers of 5, c) 12, drawn with an isovalue of 0.017. EDDB population analysis has been done at ωB97xD/def2sVP level using previously optimized DFT models. 48

NMR calculations 49

Figure S58. The correlation between calculated and experimental NMR values for 5. 49

Figure S59. The correlation between calculated and experimental NMR values for 6. 49

Figure S60. The correlation between calculated and experimental NMR values for 12. 50

Figure 61. The correlation between calculated and experimental NMR values for 13. 50

Figure S62. The correlation between calculated and experimental NMR values for 14. 51

Figure S63.The correlation between calculated and experimental NMR values for 15. 51

Table 1. Coordinates for compound 5....................51

Table 2. Coordinates for compound 6....................54

Table 3. Coordinates for compound 12....................58

Table 4. Coordinates for compound 13....................61

Table 5. Coordinates for compound 14....................64

Table 6. Coordinates for compound 15....................67

Table S7. Computational details for the optimized structures of compounds. 70

EPR 72

Figure S64. The associated $\chi_{EPR}T$ vs T plot (B) for 5....................72

Figure *S*65. X-band EPR spectra of 5 in the solid state recorded at variable temperature*s*. 72

Figure S66. The associated $\chi_{EPR}T$ vs T plot (B) for 6. 73

Catalytic reactions....................74

Figure S67. TEA dealkylation following in time by ^1H NMR spectroscopy ($CDCl_3$, 300 K, 600 Mz) with formyl signal marked. 74

Figure S68. TEA dealkylation following in time by ^1H NMR spectroscopy ($CDCl_3$, 300 K, 600 Mz). The scale of the spectra in Figure B is one-third that of Figure A. General conditions: 2 mg of catalyst 12 or 13, 0.1 mmol of tertiary amine, 1 ml of chloroform. The mixture was stirred for 3 hours at room temperature in open air under a blue light (470 nm). 75

Titrations....................76

Figure S69. Titration of 5 with HBF_4 (600 MHz, CD_2Cl_2, 200 K). 76

Figure S70. Titration of 5 A) with TFA to monocationic form, B) with HBF_4 to dicationic form (RT, DCM). 77
Figure S71. Titration of 6 with HBF_4 (RT, DCM). 78
Figure S72. Titration of 14 with HCl (RT, DCM). 78
CD spectroscopy 79
Figure S73. Changes in the CD intensity. 79
Photoluminescence 80
Figure S74. UV-Vis spectra of compounds 5, 6, 12, 13, 14, and 15. 80
Figure S75. Fluorescence decay curves of samples 5, 5+, and 14 in dichloromethane, along with the instrument response function (IRF). 80
Table S8. Fluorescence lifetime ($\boldsymbol{\tau}$), the correlation coefficient ($\boldsymbol{\chi 2}$), and fluorescence quantum yield (QY, %) for samples 5, 5+, and 14 in dichloromethane. 81

Methods

Nuclear Magnetic Resonance

NMR spectra were recorded on Bruker Avance III 500 MHz, and 600 MHz spectrometers, and JEOL JNM-ECZ500R 500 MHz spectrometer. Chemical shifts were reported in ppm with reference to residual protons and carbons of $CDCl_3$ (δ 7.24 ppm in ^{1}H NMR), CD_2Cl_2 (δ 5.32 ppm in ^{1}H NMR, δ 54.0 ppm in ^{13}C NMR), C_5D_5N (δ 8.74 ppm in ^{1}H NMR), and C_6D_6 (δ 7.16 ppm in ^{1}H NMR).

Mass spectrometry

Mass spectra were carried out on a Bruker qTOF compact spectrometer using electrospray ionization.

UV-Vis spectroscopy

UV-Vis spectra were recorded on a Varian Carry 60 using 1 cm path length optical glass/quartz cuvettes.

Luminescence

Emission spectra and luminescence decay profiles were recorded using an FLS1000 fluorescence spectrometer (Edinburgh Instruments Ltd.). Fluorescence measurements were performed with a 450 W continuous xenon arc lamp as the excitation source, while luminescence decay traces were acquired using a picosecond pulsed diode laser (EPL-450 nm, Edinburgh Instruments). The instrument was equipped with TMS302-X double-grating excitation and emission monochromators with a focal length of 325 mm. Luminescence signals were detected using a high-gain Hamamatsu R928P photomultiplier tube, thermoelectrically cooled to -22 °C. Intensity decay curves were analyzed by fitting exponential functions using Fluoracle software. Luminescence quantum yields (QY) were determined with an FLS980 spectrometer equipped with a 150 mm integrating sphere. All measurements were carried out using samples placed in 1 cm path-length quartz cuvettes.

X-Ray

Monocrystals were measured on an XtaLAB Synergy R, DW system, HyPix-Arc 150 diffractometer at 100 K, using CuKα radiation; λ = 1.54184 Å. Data reduction and analysis were carried out with the CrysAlisPro program. An absorption correction was applied. Structures were solved using the SHELXT [1] program and refined using all F2 data, as implemented by the SHELXL program [2]. All non-hydrogen atoms were further refined by SHELXL with anisotropic displacement coefficients; the positions of hydrogen atoms were calculated and refined in a riding mode.

Circular dichroism spectroscopy

Circular dichroic spectra were obtained by means of Jasco 1500 spectropolarimeter equipped with a Peltier-controlled VT equipment.

Theoretical calculations

Geometry optimizations were carried out with the Gaussian 16 software package [3] within unconstrained C1 symmetry, with starting coordinates derived from molecular mechanics or X-ray analysis. Becke's three-parameter exchange functionals with the gradient corrected correlation formula of Lee, Yang and Parr (DFT-B3LYP) [4,5] were used with the 6-31G(d,p) basis set with the LANL2DZ pseudopotential applied for Pd and Ru centers and the solvation (PCM=$CHCl_3$). Harmonic vibrational frequencies were calculated using analytical second derivatives as a verification of local minimum achievement with no negative frequencies observed. The structures were found to have converged to a minimum on the potential energy surface. Proton and carbon chemical shifts were calculated using the GIAO method and referenced to the absolute shielding of tetramethylsilane calculated at the same level of theory. The structure models consistently reflect the constraints imposed by the appropriate NOE experiments. The ^{1}H chemical shifts calculated for DFT-optimized structures of **5**–**15** are in qualitative agreement with the corresponding experimental data.

The NMR, HRMS, and DFT data files are available at doi.org/10.5281/zenodo.19221288.

Experimental

All solvents (Dichloromethane, Ethyl Acetate, Chloroform, *n*-hexane), if not indicated differently, were used without purification.

Chloroform-d_1 was prepared directly before use by passing through a basic alumina column.

Precursors of **7** and **8** were synthesized according to a literature procedure. [6]

Precursor **10** was synthesized according to a literature procedure.[7]

Precursor **11** was synthesized according to a literature procedure.[8]

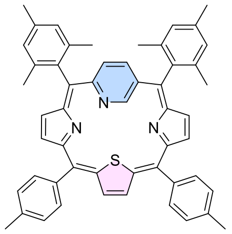

5: 5,20-Dimesityl-10,15-di-*p*-tolyl-24-thia-*p*-pyriporphyrin: 2,5-bis-[mesityl(2-pyrrolyl)methyl]-pyridine (190 mg, 0.4 mmol) and 2,5-bis[hydroxyl(*p*-tolil)methyl]thiophene (130 mg, 0.4 mmol) were added to dry $CHCl_3$ (400 mL) with 8 mL of absolute ethanol under nitrogen. Next, $Et_2O{:}BF_3$ (80 µL, 0.65 mmol) was added, and the reaction mixture was protected from light and stirred for 1 h under nitrogen. The reaction mixture was neutralized with triethylamine 92 µL (0.66 mmol) and the DDQ (284 mg, 1.25 mmol) was subsequently added. The mixture was stirred for several minutes and evaporated under reduced pressure. The residue was subjected to chromatography on a short pad of grade III basic alumina and eluted with CH_2Cl_2 (1000 mL). Solvent was evaporated one more time, and the residue was subjected to chromatography (grade II basic alumina) with CH_2Cl_2/n-hexane (1:1) at the beginning. The desired product was eluted with CH_2Cl_2/n-hexane (2:1) as a green band on the column and crimson in the solution. Recrystallisation from CH_2Cl_2/CH_3OH afforded a deep-green solid. Yield: 75 mg (25%).

^{1}H NMR ($CDCl_3$, 600 MHz, 300 K): δ 8.40 (AB, 1H, $^3J_{HH}$ = 5.7 Hz, thiophene), 8.39 (AB, 1H, $^3J_{HH}$ = 5.7 Hz, thiophene), 7.80 (d, 1H, $^3J_{HH}$ = 4.3 Hz, 7/17), 7.79 (d, 1H, $^3J_{HH}$ = 4.3 Hz, 7/17), 7.78 (br.s, 1H, *o*-Tol), 7.72 (br.d, 1H, *o*-Tol), 7.68 (AB, 1H, pyri), 7.65 (AB, 1H, pyri), 7.60 (d, $^3J_{HH}$ = 4.6 Hz, 1H, 8), 7.57 (br.d, 2H, *o*-Tol), 7.54 (d, $^3J_{HH}$ = 4.6 Hz, 1H, 18), 7.46-7.35 (m, 4H, *m*-Tol), 7.19 (s, 2H, *m*-Mes), 7.08 (s, 1H, *m*-Mes), 7.06 (s, 1H, *m*-Mes), 4.69 (s, 1H, 22), 2.55 (s, 3H, *p*-Tol), 2.54 (s, 3H, *p*-Tol), 2.48 (s, 3H, *o*-Mes), 2.47 (s, 3H, *o*-Mes), 2.46 (s, 3H, *o*-Mes), 2.45 (s, 3H, *o*-Mes) 1.75 (s, 3H, *p*-Mes), 1.64 (s, 3H, *p*-Mes).

^{13}C NMR (CDCl$_3$, 151 MHz, 300 K): δ 167.4, 166.0, 161.7, 158.6, 157.8, 155.6, 151.4, 150.7, 143.7, 142.3, 140.5, 138.8, 138.5, 138.2, 138.0, 137.8, 137.7, 137.5, 137.4, 136.5, 136.3, 136.2, 136.0, 136.0, 134.4, 132.8, 132.7, 132.6, 132.6, 132.6, 132.5, 132.4, 130.8, 130.7, 130.0,.128.72, 128.69, 128.4, 128.2, 128.1, 120.1, 22.3, 22.2, 21.55, 21.51, 21.48.

HRMS (ESI) m/z: $[M+H]^+$ calc. for. $C_{53}H_{46}N_3S$ 756.3407; found 756.3935.

UV-Vis (CH_2Cl_2) λmax [nm] (logε) = 425 nm (4.39), 565 nm (4.02).

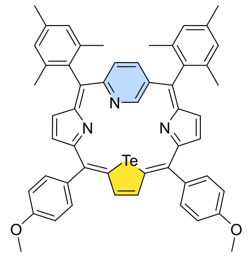

6: 10,15-Di-*p*-anisole-5,20-dimesityl-24-tellura-*p*-pyriporphyrin: In the same condensation and isolation conditions as for 5,20-dimesityl-10,15-di-*p*-tolyl-24-thia-*p*-pyriporphyrin, 24-tellura analog was obtained when 2,5-bis[*p*-anisol(hydroxyl)methyl]-tellurophene (181 mg, 0.4 mmol) instead of 2,5-bis[hydroxyl-(*p*-tolyl)methyl]thiophene was used. Yield: 74 mg (21%).

^{1}H NMR (CDCl$_3$, 500 MHz, 300 K): δ 8.50 (AB, $^3J_{HH}$ = 7.07 Hz, 1H, tell), 8.48 (AB, $^3J_{HH}$ = 7.07 Hz, 1H, tell), 7.59 (br.s, 2H, *o*-Anis), 7.57 (d, $^3J_{HH}$ = 4.7 Hz, 1H, 18), 7.56 (br.s, 2H, *o*-Anis), 7.55 (d, $^3J_{HH}$ = 4.7 Hz, 1H, 7), 7.40 (dd, $^3J_{HH}$ = 8.2 Hz, $^4J_{HH}$ = 2.2 Hz, 1H, 3), 7.38 (d, $^3J_{HH}$ = 4.7 Hz, 1H, 8), 7.34 (d, $^3J_{HH}$ = 4.7 Hz, 1H, 17), 7.23 (d, $^3J_{HH}$ = 8.2 Hz, 1H, 2), 7.13-7.06 (m, 6H, *m*-Mes, *m*-Tol), 7.03 (s, 2H, Mes), 6.48 (d, $^4J_{HH}$ = 2.2 Hz, 1H, 2), 3.93 (s, 3H, *p*-Anis), 3.92 (s, 3H, *p*-Anis), 2.45 (s, 3H, *o*-Mes), 2.42 (s, 3H, *o*-Mes), 2.421 (s, 3H, *o*-Mes), 2.413 (s, 3H, *o*-Mes), 1.85 (s, 3H, *p*-Mes), 1.79 (s, 3H, *p*-Mes).

^{13}C NMR (CDCl$_3$, 151 MHz, 300 K): δ 171.3, 170.7, 162.3, 159.1, 157.7, 156.0, 155.9, 154.8, 154.0, 143.2, 143.0, 142.6, 142.2, 140.2, 138.6, 138.5, 138.4, 137.9, 137.5, 137.3, 137.1, 136.3, 135.9, 134.8, 133.1, 133.0, 132.4, 132.3, 131.3, 131.1, 131.0, 128.8, 128.3, 128.2, 119.5, 113.6, 113.5, 55.5, 22.0, 21.9, 21.43, 21.41, 21.2, 21.0.

HRMS (ESI) m/z: $[M+H]^+$ calc. for. $C_{53}H_{46}N_3O_2Te$ 886.2654; found 886.2615

UV-Vis (CH_2Cl_2) λmax [nm] (logε) = 305 (4.18), 413 nm (4.37), 636 nm (4.07).

Palladium(II) complexes:

Under anaerobic conditions, a mixture of $CHCl_3$/MeCN was used to dissolve palladium(II) chloride (3.5 mg; 19.85 μmol; 1.50 equiv.) and either 24-tellura-*p*-pyriporphyrin (11.7 mg; 13.19 μmol) or 24-thia-*p*-pyriporphyrin (10 mg; 13.23 μmol). The mixture was heated (using a heating mantle) and stirred under a reflux condenser for one hour. The solution was then cooled to room temperature, and the mixture was separated using silica gel chromatography.

The eluent was dichloromethane and ethyl acetate (10:1). Yields: **12** – 70% (8,5 mg), **13** – 62% (8,2 mg).

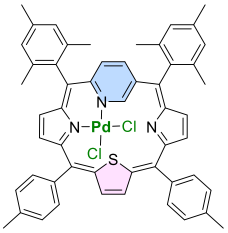

12: ^{1}H NMR ($CDCl_3$, 600 MHz, 300 K): *δ* 9.06 (AB, $^3J_{HH}$ = 5.5 Hz, 1H, tell), 9.04 (AB, $^3J_{HH}$ = 5.5 Hz, 1H, tell), 8.54 (dd, $^3J_{HH}$ = 8.3 Hz, $^4J_{HH}$ = 1.8 Hz, 1H, pyri), 8.13 (d, $^3J_{HH}$ = 8.4 Hz, 1H, pyri), 8.07 (d, $^3J_{HH}$ = 7.5 Hz, 1H, *o*-Tol), 8.04 (d, $^3J_{HH}$ = 4.5 Hz, 1H, pyrr), 7.96 (d, $^3J_{HH}$ = 4.5 Hz, 1H, pyrr), 7.78 (d, $^3J_{HH}$ = 4.8 Hz, 1H, pyrr), 7.69 (d, $^3J_{HH}$ = 7.8 Hz, 1H, *m*-Tol) 7.61 (d, $^3J_{HH}$ = 7.8 Hz, 1H, *m*-Tol), 7.42 (s, 1H, *m*-Mes), 7.35 (d, $^3J_{HH}$ = 5.1 Hz, 1H, β-pyrr), 7.27 (s, 1H, *m*-Mes), 7.05 (s, 1H, *m*-Mes), 6.98 (s, 1H, *m*-Mes), 3.76 (d, $^3J_{HH}$ = 1.9 Hz, 1H, 22), 3.11 (s, 3H, *p*-Tol/*o*-Mes), 2.63 (s, 3H, *p*-Tol/*o*-Mes), 2.59 (s, 3H, *p*-Mes, *p*-Tol/*o*-Mes), 2.51 (s, 3H, *p*-Tol/*o*-Mes), 2.50 (s, 3H, *p*-Tol/*o*-Mes), 2.49 (s, 3H, *p*-Tol/*o*-Mes), 1.48 (s, 3H, *p*-Mes), 0.74 (s, 3H, *p*-Mes).

^{13}C NMR ($CDCl_3$, 151 MHz, 300 K): *δ* 172.7, 166.0, 165.8, 160.1, 154.0, 153.1, 151.2, 148.6, 143.7, 142.7, 142.2, 141.5, 139.8, 139.8, 139.6, 139.1, 138.9, 138.6, 137.2, 136.7, 136.5, 136.2, 135.4, 135.0, 134.8, 134.1, 133.8, 133.1, 132.9, 132.4, 132.0, 131.5, 129.3, 129.3, 129.0, 128.6, 128.5, 128.3, 128.2, 121.8, 22.7, 22.5, 21.5, 21.4, 21.3, 20.7, 19.7.

HRMS (ESI) m/z: $[M-Cl+CH_3CN]^+$ calc. for $C_{55}H_{48}ClN_4PdS$ 939.2325; found 939.2285.

UV-Vis (CH_2Cl_2) λmax [nm] (logε) = 311 (4.68), 423 (4.87), 462 (4.87), 625 (4.44), 744 (4.11).

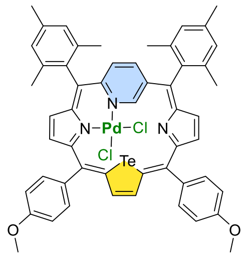

13: ^{1}H NMR ($CDCl_3$, 600 MHz, 300 K): *δ* 9.28 (AB, $^3J_{HH}$ = 6.8 Hz, 1H, tell), 9.24 (AB, $^3J_{HH}$ = 6.8 Hz, 1H, tell), 8.16 (dd, $^3J_{HH}$ = 8.4 Hz, $^4J_{HH}$ = 2.1 Hz, 1H, pyri), 7.98 (d, $^3J_{HH}$ = 8.4 Hz, 2H, *o*-Anis), 7.80 (d, $^3J_{HH}$ = 4.7 Hz, 1H, pyrr), 7.79 (d, $^3J_{HH}$ = 4.7 Hz, 1H, pyrr), 7.78 ($^3J_{HH}$ = 4.8 Hz, 1H, pyrr) 7.82-7.76 (br.s, 2H, *o*-Anis), 7.59 (d, $^3J_{HH}$ = 8.4 Hz, pyri), 7.38 (d, $^3J_{HH}$ = 4.8 Hz, 1H, pyrr), 7.35 (s, 1H, *m*-Mes), 7.18 (m, 2H, *m*-Anis), 7.17 (s, 1H, *m*-Mes), 7.05 (s, 1H, *m*-Mes), 6.94 (s, 1H, *m*-Mes), 5.40 (d, $^3J_{HH}$ = 2.1 Hz, 1H, 22), 4.0 (s, 3H, OCH_3), 3.96 (s, 3H, OCH_3), 3.06 (s, 3H, Mes), 2.45 (s, 3H, Mes), 2.42 (s, 3H, Mes), 2.41 (s, 3H, Mes), 1.70 (s, 3H, Mes), 0.82 (s, 3H, Mes).

^{13}C NMR ($CDCl_3$, 151 MHz, 300 K): *δ* 182.0, 169.2, 167.4, 162.2, 160.3, 160.1, 157.6, 154.2, 153.1, 148.3, 146.7, 145.4, 144.8, 142.7, 141.6, 141.1, 141.0, 139.9, 139.8, 139.7, 139.1, 136.6, 136.5, 135.9, 135.6, 135.5, 134.5, 133.9, 133.2, 133.0, 131.9, 131.3, 130.7, 129.4, 128.4, 128.2, 121.2, 114.1, 113.7, 55.6, 55.6, 22.6, 22.3, 21.4, 21.0, 19.7.

HRMS (ESI) m/z: $[M-Cl]^+$ calc. for $C_{53}H_{45}ClN_3O_2PdTe$ 1026.1293; found 1026.1233.

UV-Vis (CH_2Cl_2) λmax [nm] (logε) = 343 (4.44), 454 (4.61), 633 (4.11), 785 (4.06).

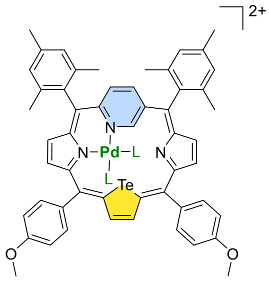
13-2+: ^{1}H NMR (CDCl$_3$, 600 MHz, 300 K): δ 9.70 (d, $^3J_{HH}$ = 6.6 Hz, 1H, tell), 9.65 (d, $^3J_{HH}$ = 6.6 Hz, 1H, tell), 8.42 (dd, $^3J_{HH}$ = 8.5 Hz, $^4J_{HH}$ = 2.0 Hz, 1H, pyri), 8.04 (d, $^3J_{HH}$ = 4.7 Hz, 1H, pyrr), 8.01 (d, $^3J_{HH}$ = 4.8 Hz, 1H, pyrr), 7.99 (d, $^3J_{HH}$ = 4.7 Hz, 1H, pyrr), 7.95 (d, $^3J_{HH}$ = 7.8 Hz, 1H, *o*-Anis), 7.86 (d, $^3J_{HH}$ = 8.5 Hz, pyri), 7.83 (d, $^3J_{HH}$ = 8.4 Hz, 2H, *o*-Anis), 7.66 (d, $^3J_{HH}$ = 4.3 Hz, 2H, pyrr), 7.38–7.29 (m, 6H, *m*-Anis, Mes), 7.20 (s, 2H, Mes), 7.04 (s, 1H, Mes), 6.97 (s, 1H, Mes), 4.44 (d, $^3J_{HH}$ = 2.0 Hz, 1H, 22), 4.01(s, 3H, OCH$_3$), 3.99 (s, 3H, OCH$_3$), 2.96 (s, 3H), 2.46 (s, 3H, Mes), 2.43 (s, 3H, Mes), 2.41 (s, 6H, Mes), 1.58 (s, 3H).

Ruthenium(II) complexes:

24-Tellura-*p*-pyriporphyrin (10.5 mg, 11.88 µmol) or 24-thia-*p*-pyriporphyrin (9 mg, 11.90 µmol) was dissolved in dichloromethane and stirred with pentamethylcyclopentadienyltris(acetonitrile)ruthenium(II) hexafluorophosphate (15 mg, 24.28 µmol, 2.04 equivalents). The reaction was carried out in a glove box under oxygen-free conditions. The solution was stirred at room temperature for 24 hours and then purified by silica gel chromatography. The eluents were dichloromethane and a mixture of dichloromethane and ethyl acetate, with increasing ester content. Yields: **14** - 94% (10.5 mg), **15** - 90% (12 mg).

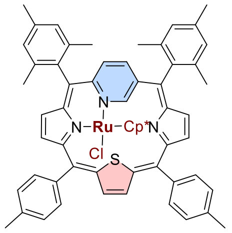
14: ^{1}H NMR (CDCl$_3$, 600 MHz, 300 K): δ 9.23 (AB, $^3J_{HH}$ = 5.6 Hz, 1H, thiophen), 9.19 (m, $^3J_{HH}$ = 5.6 Hz, 1H, thiophen), 8.46 (dd, $^3J_{HH}$ = 8.6 Hz, $^4J_{HH}$ = 2.0 Hz, 1H, pyri), 8.36 (d, $^3J_{HH}$ = 8.6 Hz, pyri), 7.90 (d, $^3J_{HH}$ = 4.6 Hz, pyrr), 7.83 (d, $^3J_{HH}$ = 4.9 Hz, pyrr), 7.74 (m, 1H, *o*-Tol), 7.69 (d, $^3J_{HH}$ = 4.6 Hz, pyrr), 7.60 (m, 2H, *m*-Tol), 7.53 (d, $^3J_{HH}$ = 8.1 Hz, *m*-Tol), 7.49 (d, $^3J_{HH}$ = 4.9 Hz, pyrr), 7.33 (s, 3H, *m*-Mes), 7.27 (s, 3H, *m*-Mes), 7.06 (s, 3H, *m*-Mes), 6.93 (s, 3H, *m*-Mes), 5.31 (d, $^4J_{HH}$ = 2.0 Hz, 1H, 22), 2. 78 (s, 3H, Mes), 2.61 (s, 6H, *p*-Tol), 2.53 (s, 3H, Mes), 2.48 (s, 6H, Mes), 1.54 (s, 3H, Mes), 0.65 (s, 3H, Mes), 0.35 (s, 15H, Cp*).

^{13}C NMR (CDCl$_3$, 151 MHz, 300 K): δ 168.1, 166.4, 163.9, 160.0, 152.5, 152.1, 151.0, 142.8, 142.5, 141.6, 140.2, 140.1, 139.7, 139.5, 139.3, 138.4, 138.3, 137.7, 137.1, 137.0, 136.4, 136.1, 136.0, 135.7, 135.2, 134.9, 134.7, 134.1, 133.3, 132.9, 132.7, 132.4, 131.7, 131.4,

129.8, 129.4, 129.3, 129.1, 128.9, 128.8, 128.6, 128.4, 78.7, 22.7, 22.0, 21.5, 21.4, 21.3, 20.8, 19.5, 8.1.

HRMS (ESI) m/z: [M-Cl]$^+$ calc. for. $C_{63}H_{60}N_3RuS$ 992.3562; found 992.3462.

UV-Vis (CH_2Cl_2) λmax [nm] (logε) = 436 (4.92), 554 (4.29), 784 (3.81).

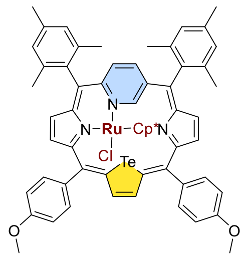

15: ^{1}H NMR ($CDCl_3$, 600 MHz, 300 K): δ 9.50 (d, $^3J_{HH}$ = 6.5 Hz, 1H, tell), 9.36 (d, $^3J_{HH}$ = 6.5 Hz, 1H, tell), 8.00 (dd, $^3J_{HH}$ = 8.6 Hz, $^4J_{HH}$ = 1.9 Hz, 1H, pyri), 7.93 (d, $^3J_{HH}$ = 8.6 Hz, 1H, pyri), 7.87 (d, $^3J_{HH}$ = 8.1 Hz, 2H, *o*-Anis), 7.78 (d, $^3J_{HH}$ = 4.9 Hz, 1H, pyrr), 7.76 (d, $^3J_{HH}$ = 4.6 Hz, 1H, pyrr), 7.69 (d, $^3J_{HH}$ = 4.6 Hz, 1H, pyrr), 7.52 (d, $^3J_{HH}$ = 4.9 Hz, 1H, pyrr), 7.32 (d, $^3J_{HH}$ = 7.9 Hz, 2H, *m*-Anis), 7.29 (s, 3H, *m*-Mes), 7.28-7.21 (m, 5H, *m*-Mes, *m*-Anis), 7.01 (s, 3H, *m*-Mes), 6.96 (s, 3H, *m*-Mes), 6.36 (dd, $^4J_{HH}$ = 1.9 Hz, 1H, pyri), 4.02 (s, 3H, OCH_3), 4.00 (s, 3H, OCH_3), 2.73 (s, 3H, Mes), 2.54 (s, 3H, Mes), 2.46 (s, 3H, Mes), 2.45 (s, 3H, Mes), 1.49 (s, 3H, Mes), 0.87 (s, 3H, Mes), 0.54 (s, 15H, Cp*).

HRMS (ESI) m/z: [M-Cl]$^+$ calc. for. $C_{63}H_{60}N_3O_2RuTe$ 1120.2793; found 1120.2796.

UV-Vis (CH_2Cl_2) λmax [nm] (logε) = 334 (4.45), 430 (4.52), 588 (4.13), 805 (3.64).

Figures

NMR

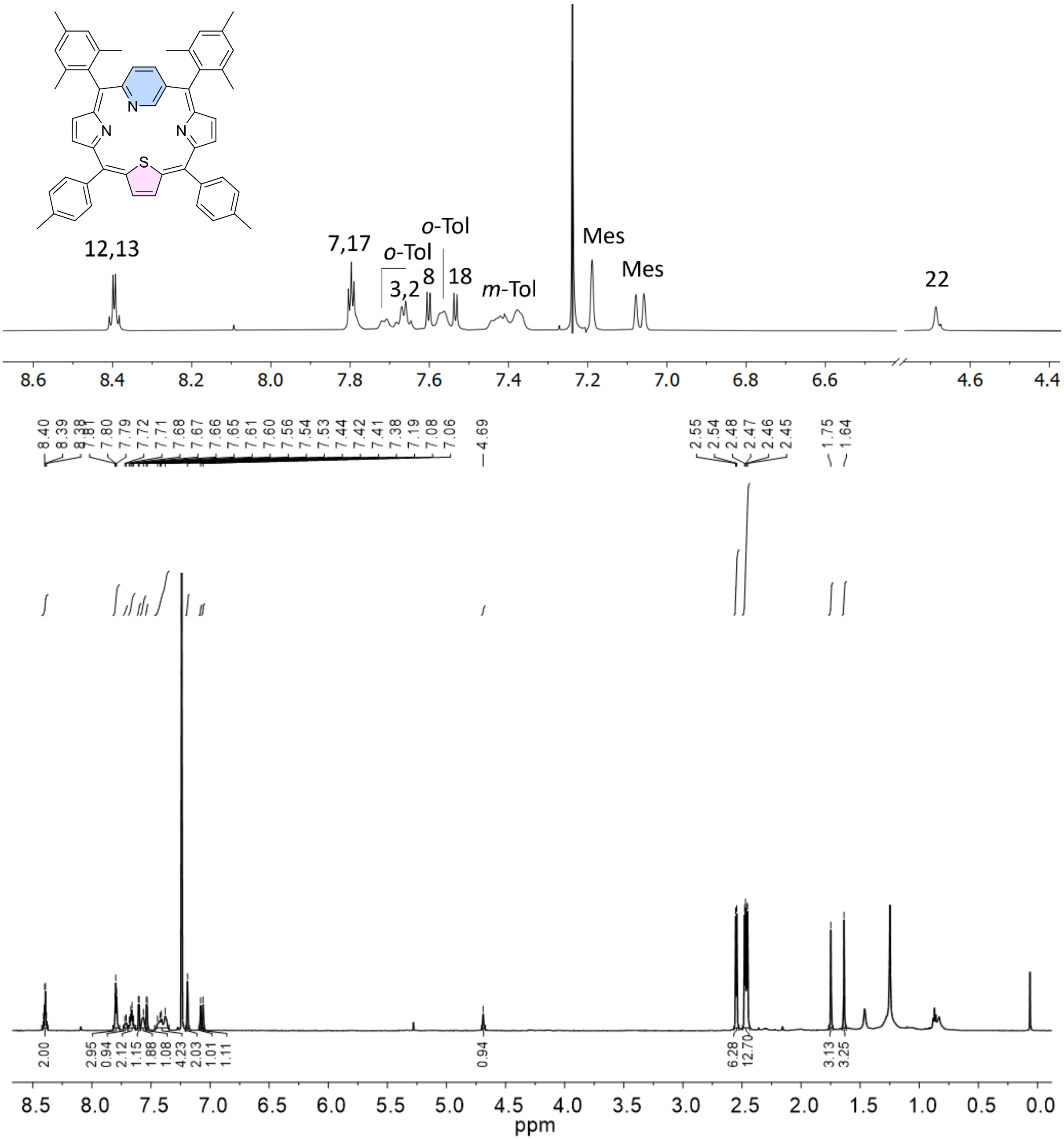

Figure S1. Signals assignment - part of the ^{1}H NMR spectrum of 5 (top; 600 MHz, 300 K, $CDCl_3$); The ^{1}H NMR spectrum of 5 (bottom; 600 MHz, 300 K, $CDCl_3$).

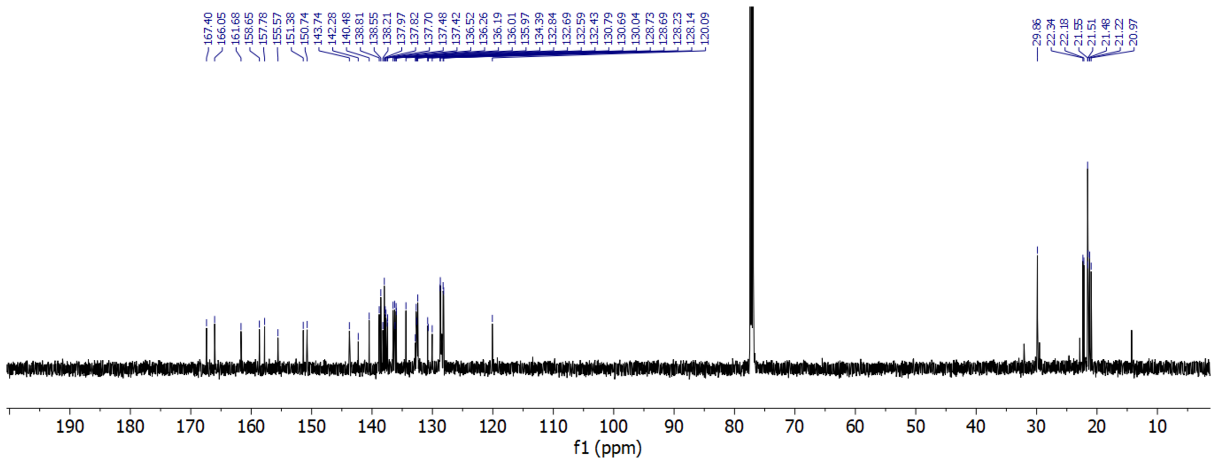

Figure S2. The ^{13}C NMR spectrum of 5 (600 MHz, 300 K, $CDCl_3$).

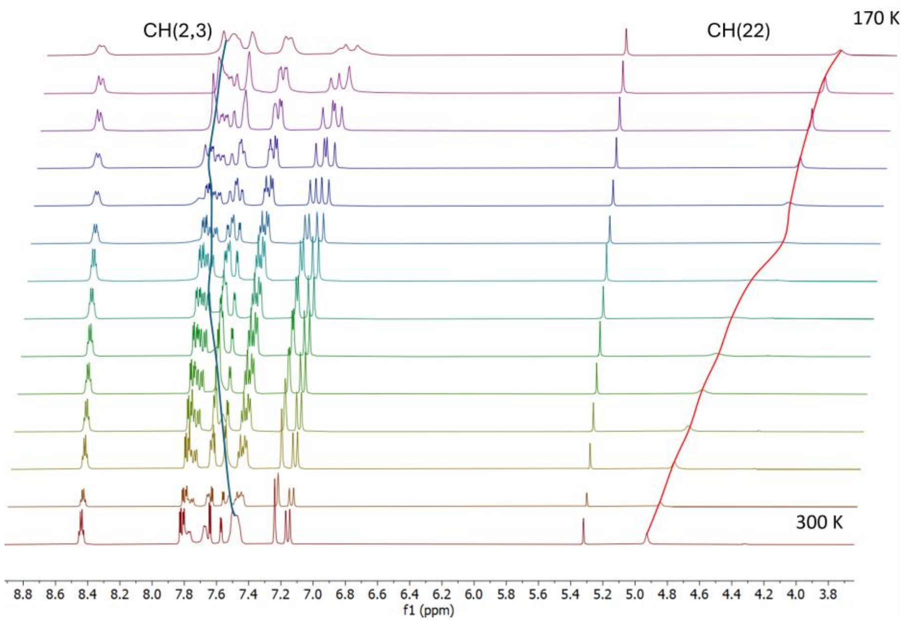

Figure S3. Part of the ^{1}H NMR spectrum of 5 at temperatures ranging from 300 K to 170 K, with a 10 K interval between each temperature (600 MHz, VT, CD_2Cl_2).

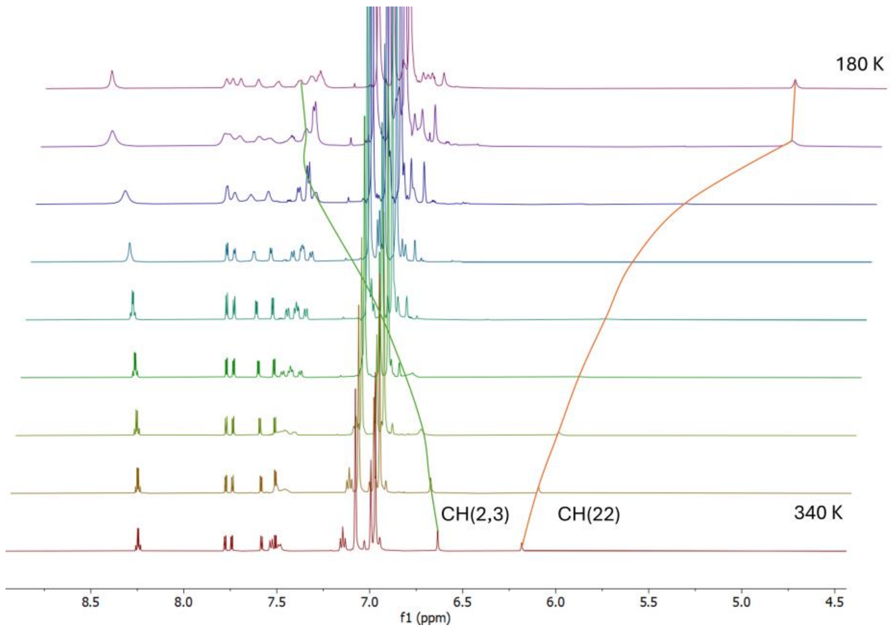

Figure S4. Part of the ^{1}H NMR spectrum of 5 at temperatures ranging from 340 K to 180 K, with a 20 K interval between each temperature (600 MHz, VT, Tol-d_8). Stacked angle: 5%.

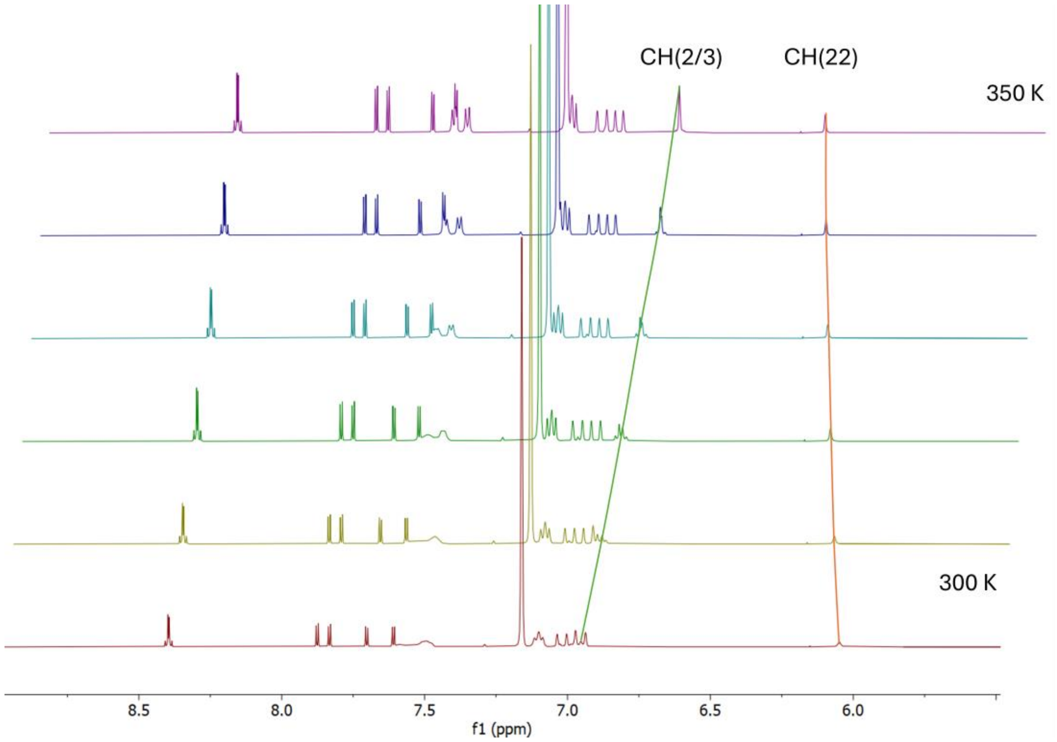

Figure S5. Part of the ^{1}H NMR spectrum of 5 at temperatures ranging from 300 K to 350 K, with a 10 K interval between each temperature (600 MHz, VT, C_6D_6). Stacked angle: 5%.

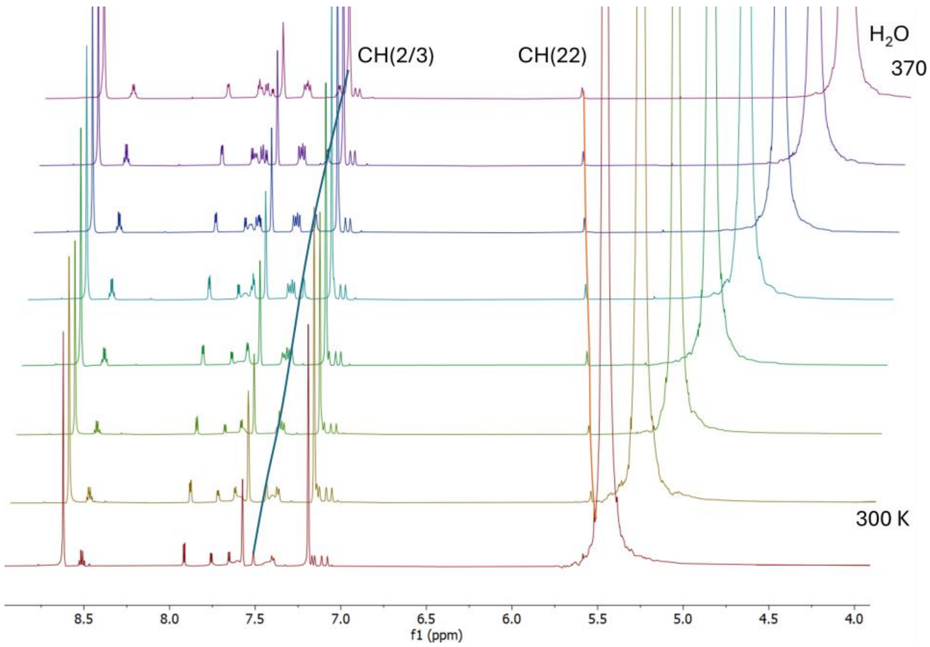

Figure S6. Part of the ^{1}H NMR spectrum of 5 at temperatures ranging from 300 K to 370 K, with a 10 K interval between each temperature (600 MHz, VT, Py-d_5). Stacked angle: 5%.

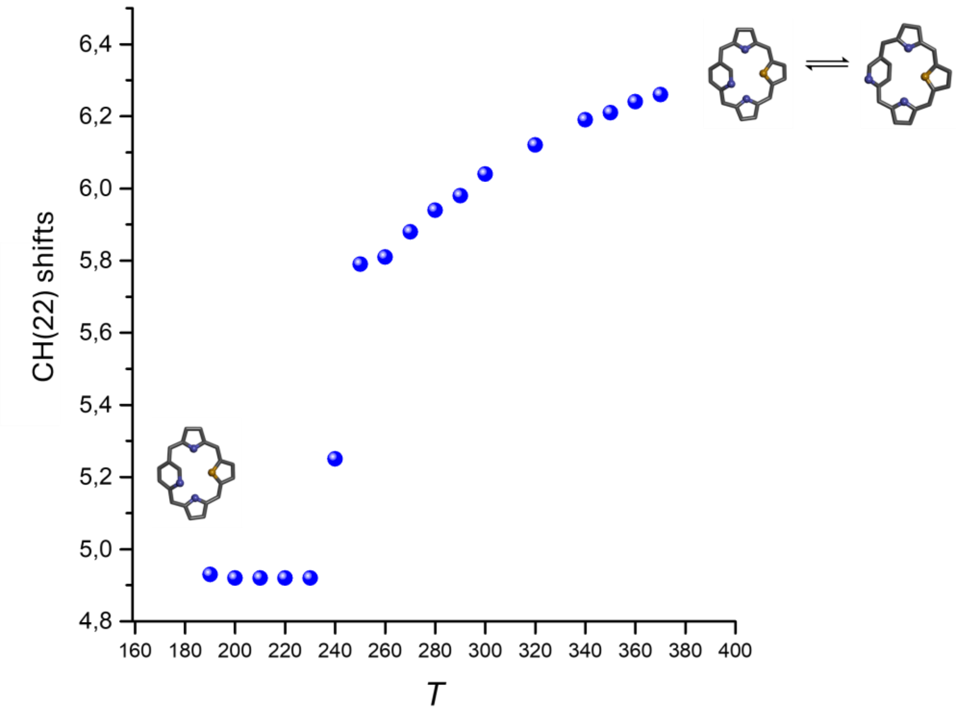

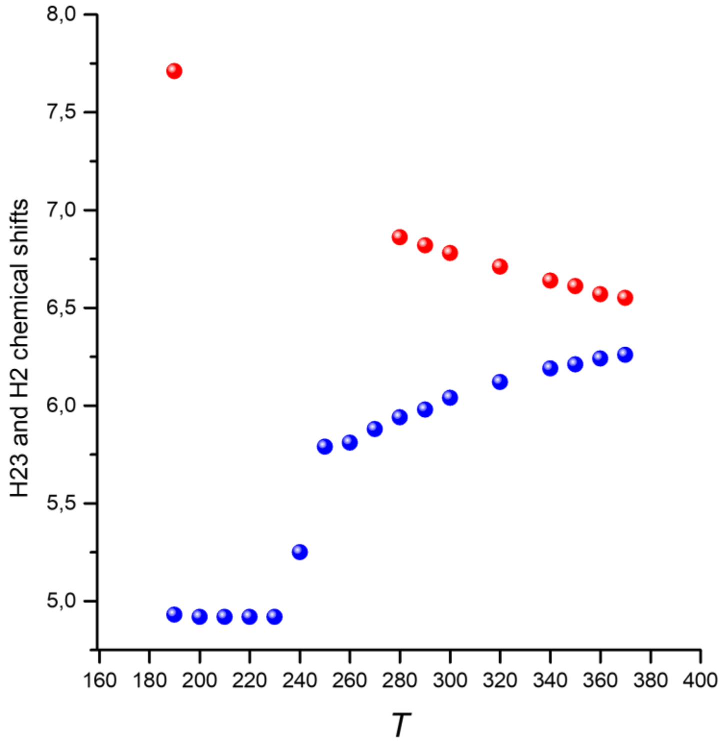

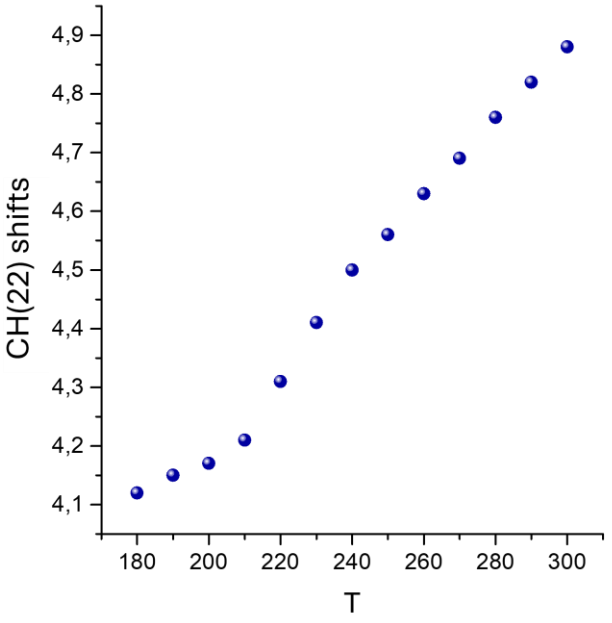

Figure S7. Changes in the position of the signal originating from the CH(22) group in the ^{1}H NMR spectrum as a function of temperature (top: *Tol-d$_8$*, VT, 600 MHz, middle: *Tol-d$_8$*, VT, 600 MHz, bottom: *CD$_2$Cl$_2$*, VT, 600 MHz).

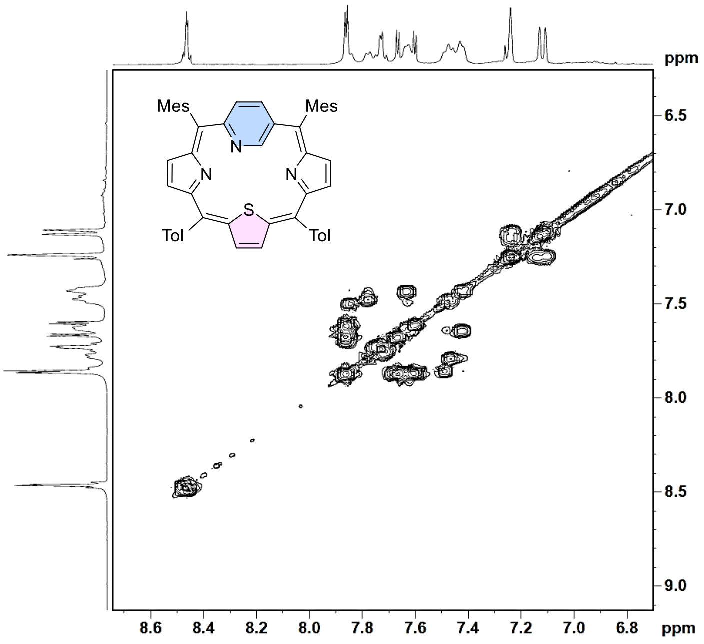

Figure S8. Part of the COSY spectrum of 5 (600 MHz, 300 K, CDCl$_3$).

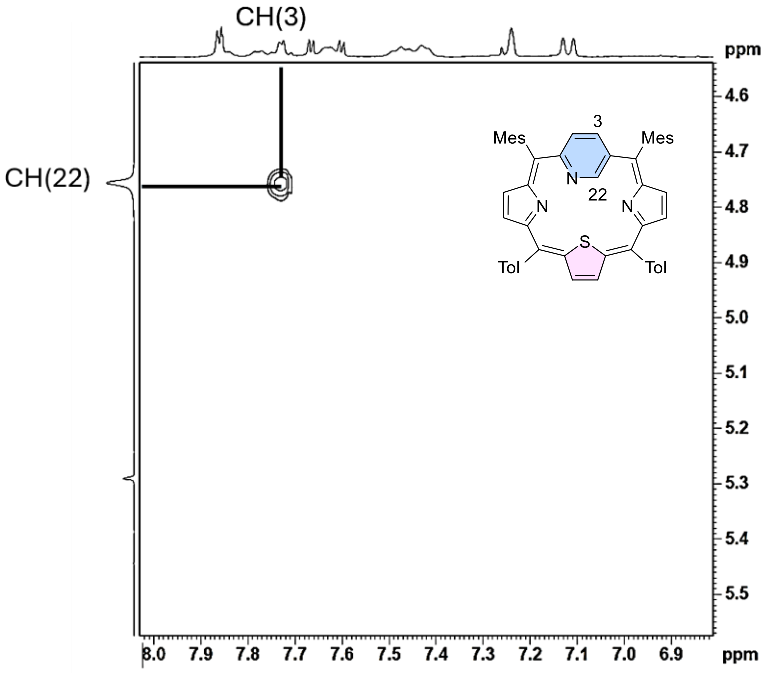

Figure S9. Part of the COSY spectrum of 5 (600 MHz, 300 K, CDCl$_3$).

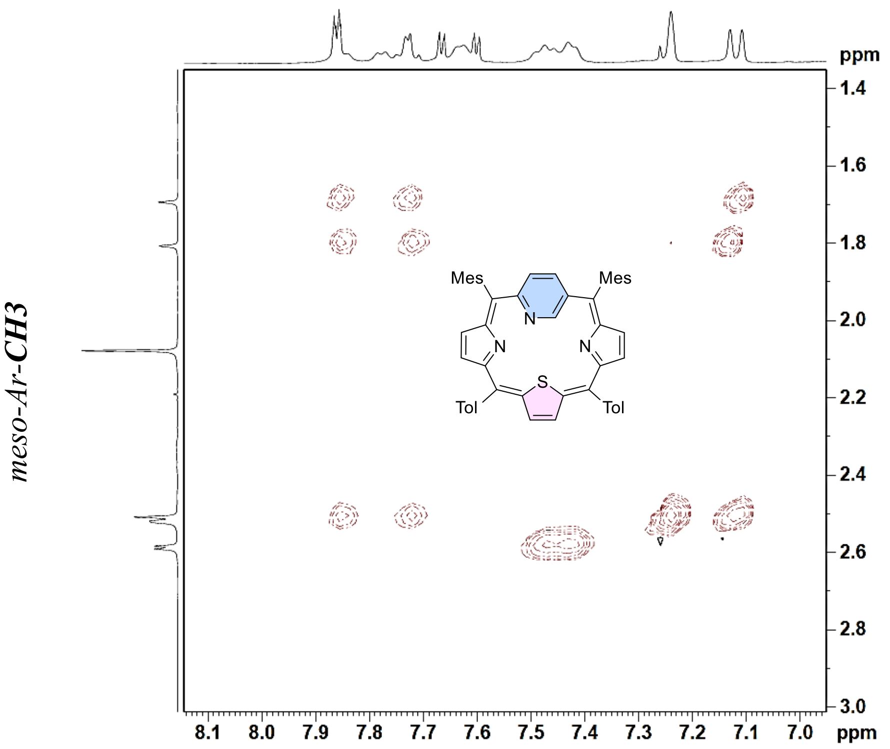

Figure S10. Part of the NOESY spectrum of 5 (600 MHz, 300 K, $CDCl_3$).

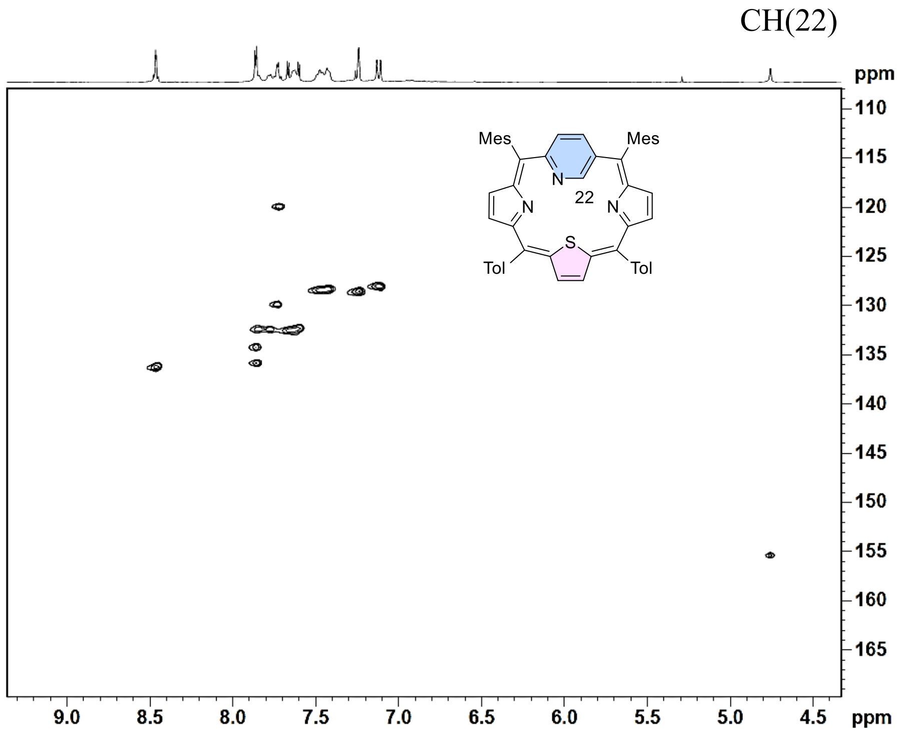

Figure S11. Part of the HSQC spectrum of 5 (600 MHz, 300 K, $CDCl_3$).

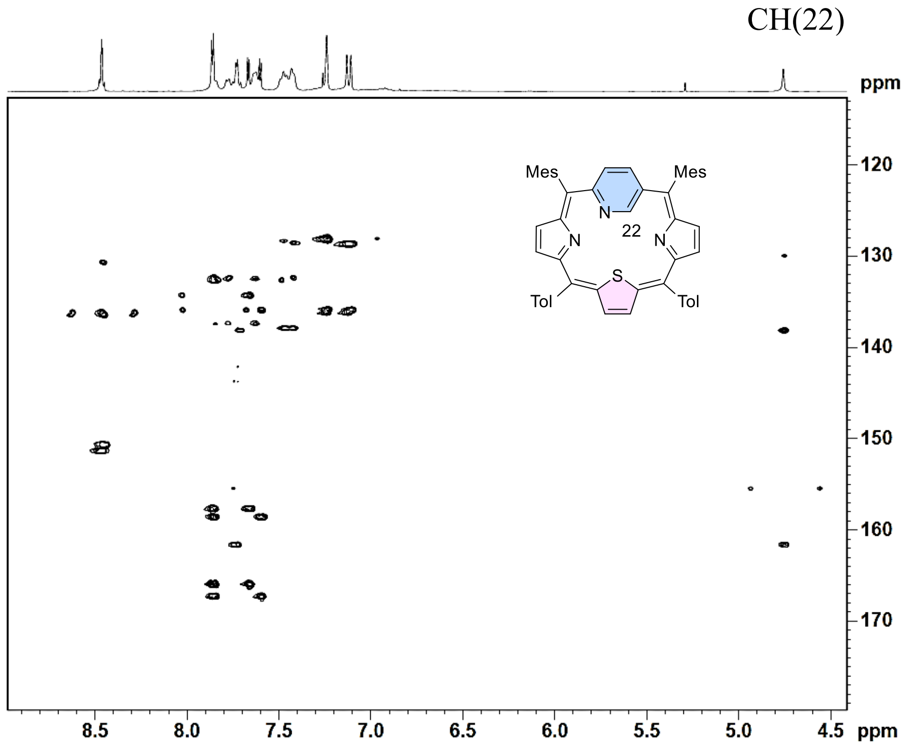

Figure S12. Part of the HMBC spectrum of 5 (600 MHz, 300 K, $CDCl_3$).

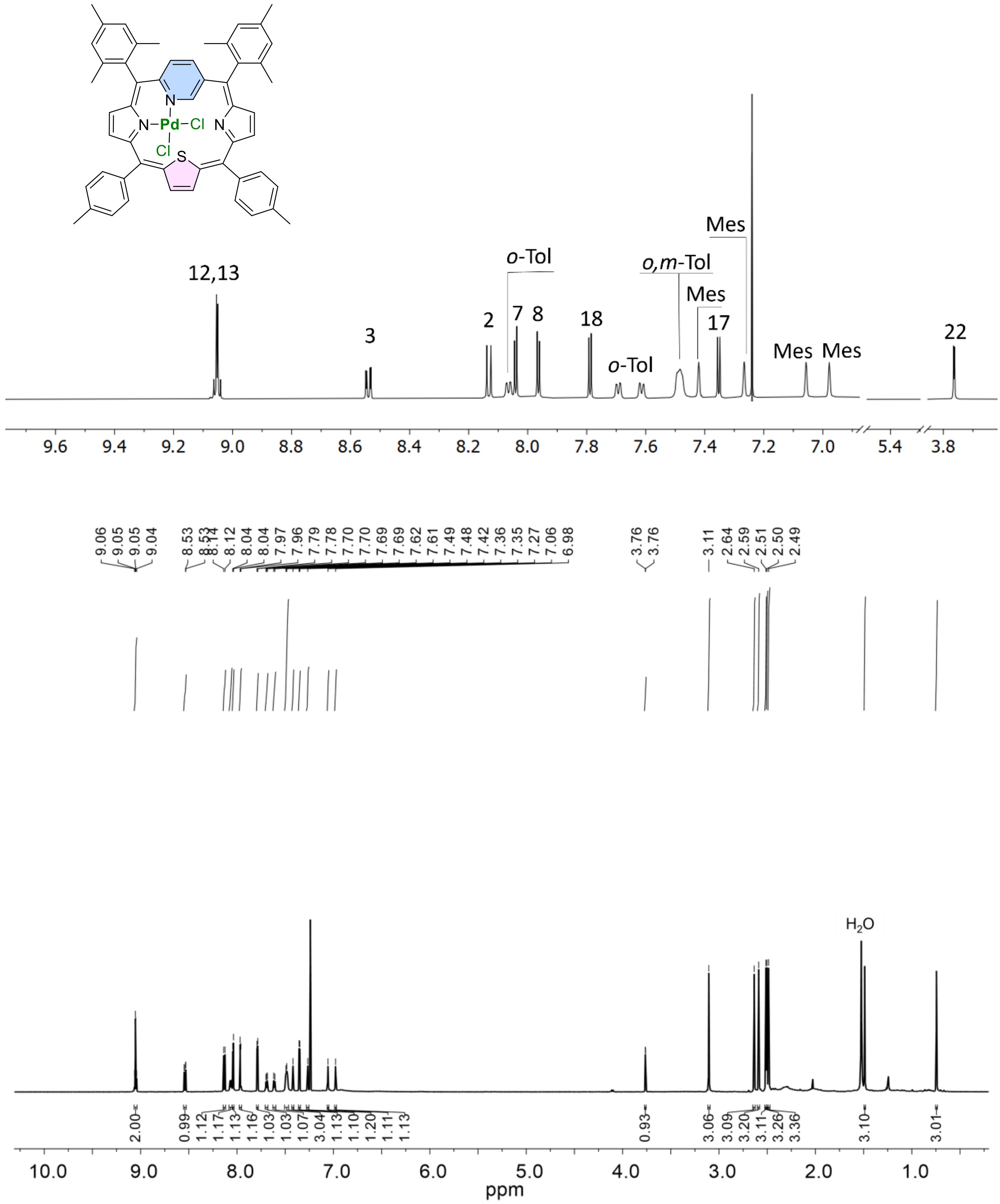

Figure S13. Signals assignment - part of the ^{1}H NMR spectrum of 12 (top, 600 MHz, 300 K, $CDCl_3$), the ^{1}H NMR spectrum of 12 (bottom, 600 MHz, 300 K, $CDCl_3$).

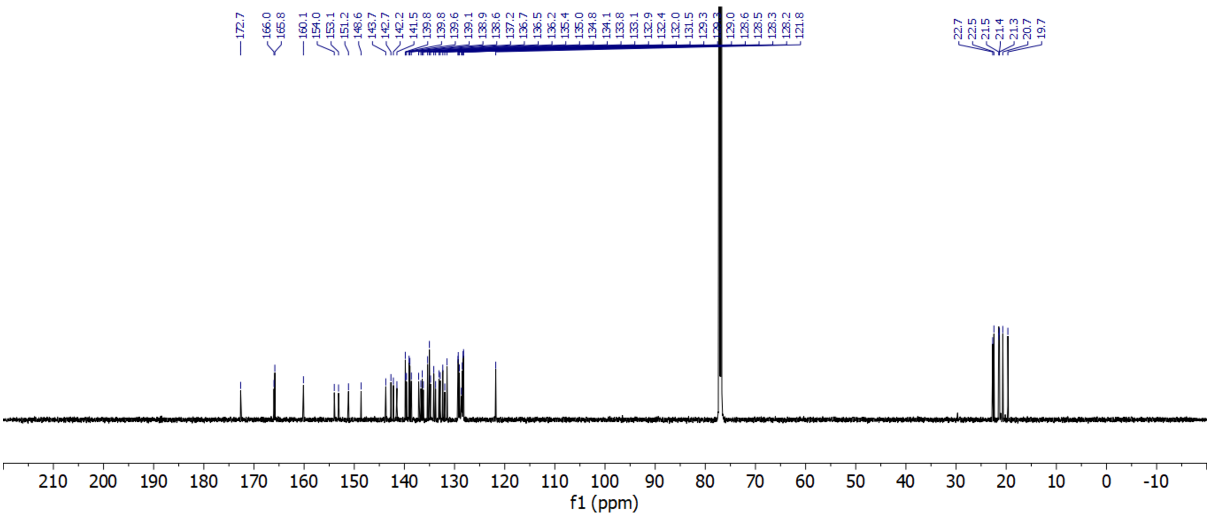

Figure S14. The ^{13}C NMR spectrum of 12 (600 MHz, 300 K, $CDCl_3$).

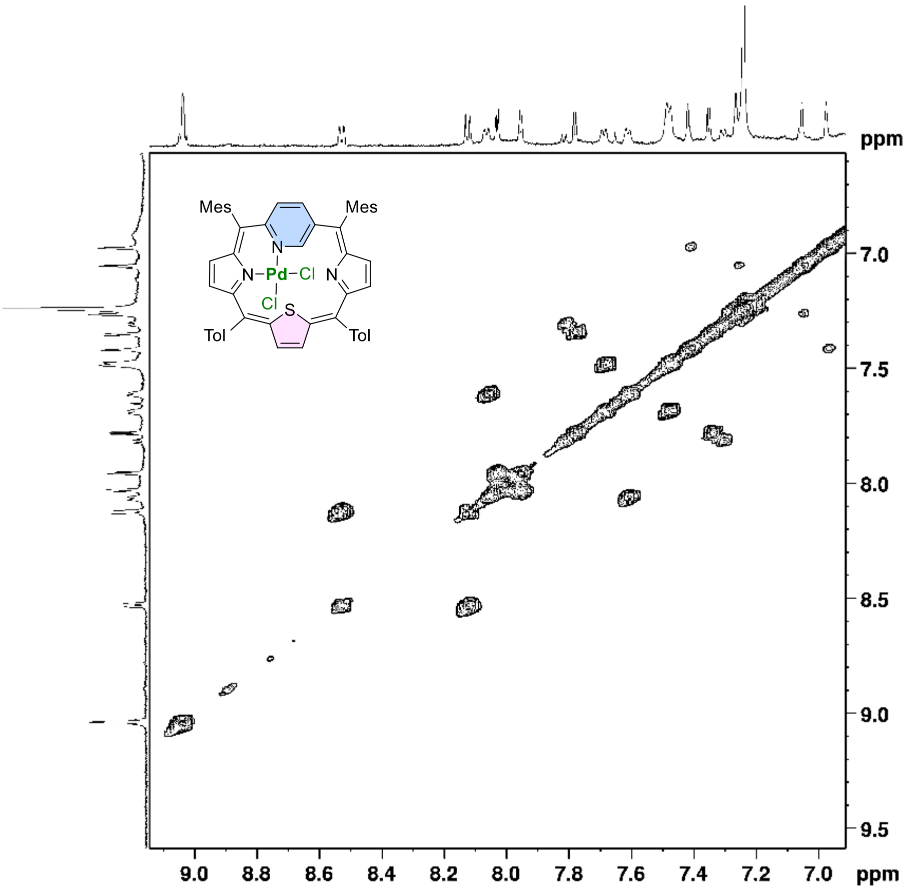

Figure S15. Part of the COSY spectrum of 12 (600 MHz, 300 K, $CDCl_3$).

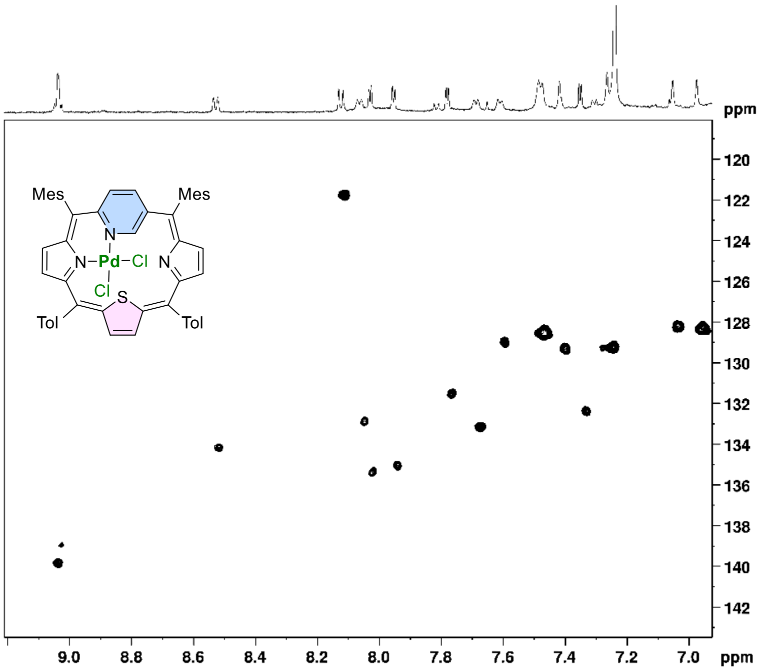

Figure S16. Part of the HSQC spectrum of 12 (600 MHz, 300 K, $CDCl_3$).

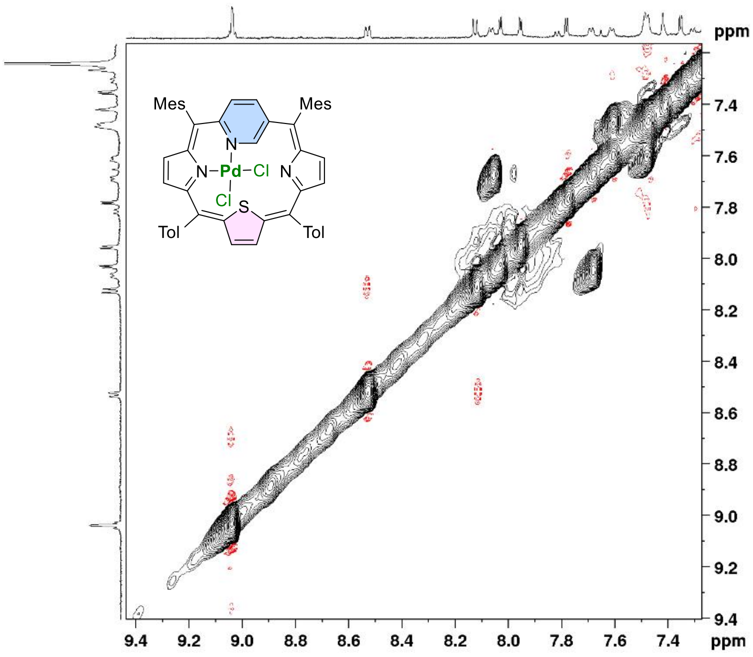

Figure S17. Part of the NOESY spectrum of 12 (600 MHz, 300 K, $CDCl_3$).

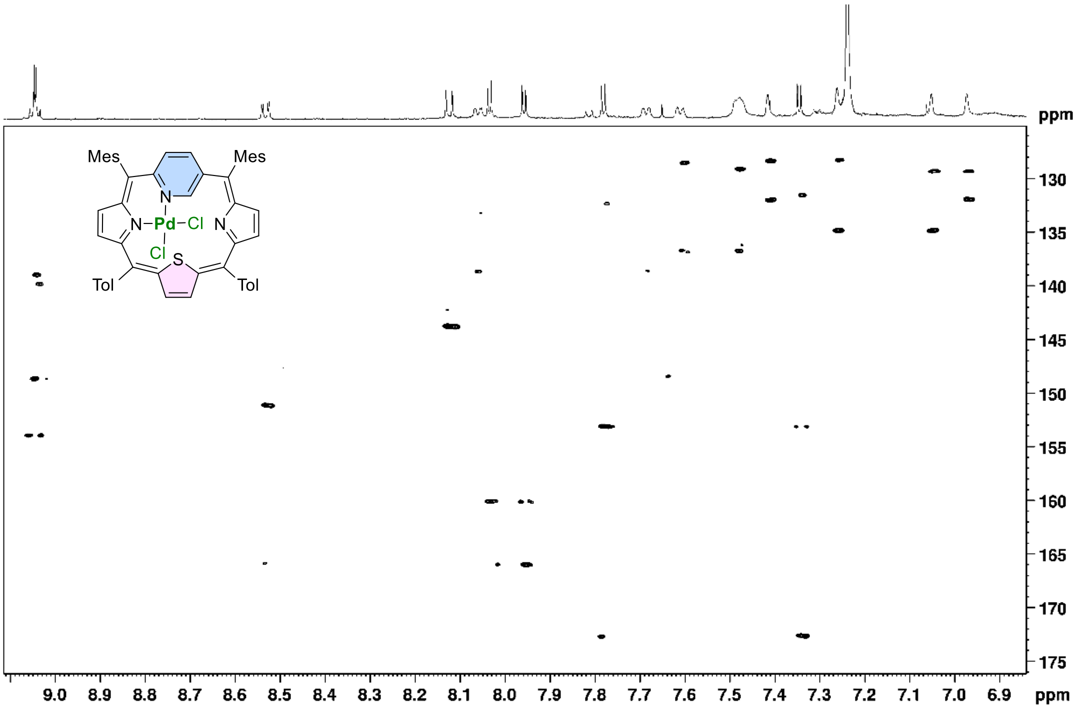

Figure S18. Part of the HMBC spectrum of 12 (600 MHz, 300 K, CDCl₃).

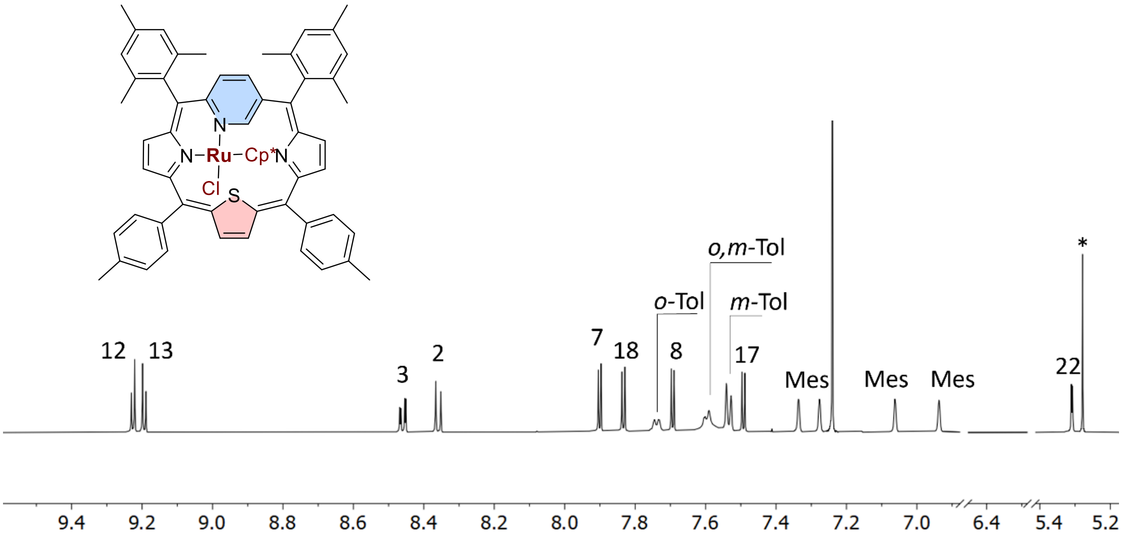

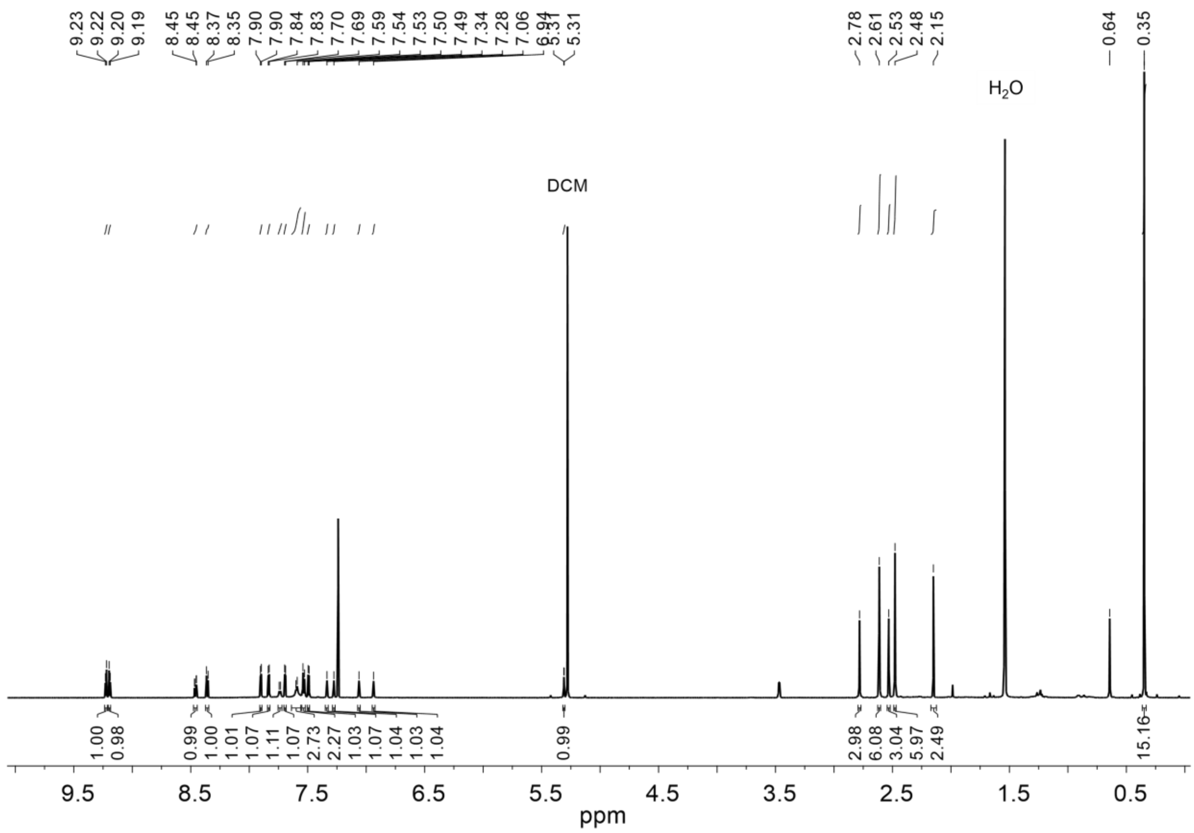

Figure S19. Signals assignment - part of the ^{1}H NMR spectrum of 14 (top; 600 MHz, 300 K, $CDCl_3$), the ^{1}H NMR spectrum of 14 (bottom; 600 MHz, 300 K, $CDCl_3$).

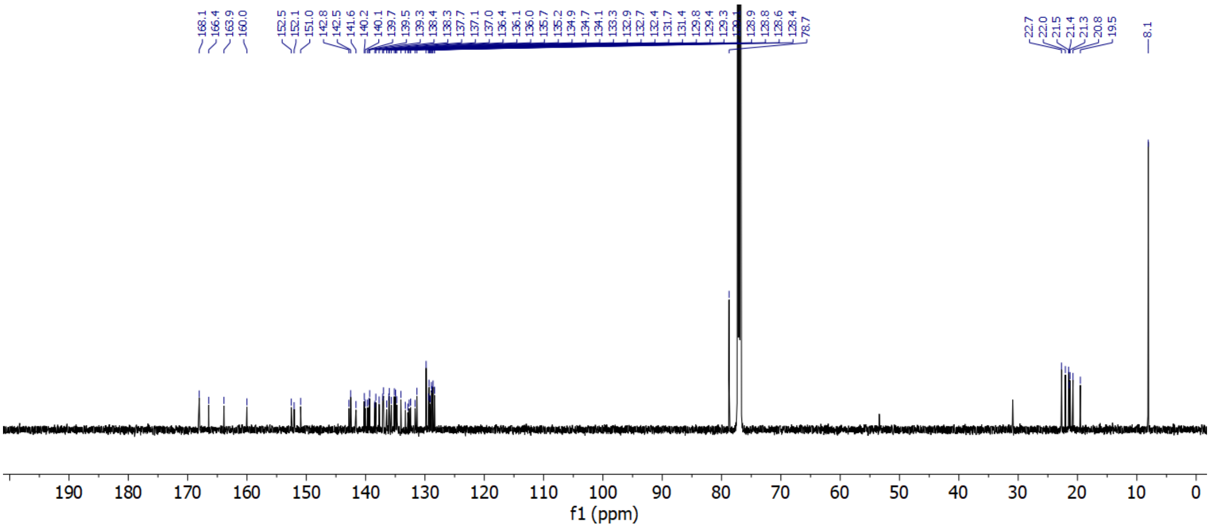

Figure S20. The ^{13}C NMR spectrum of 14 (600 MHz, 300 K, $CDCl_3$).

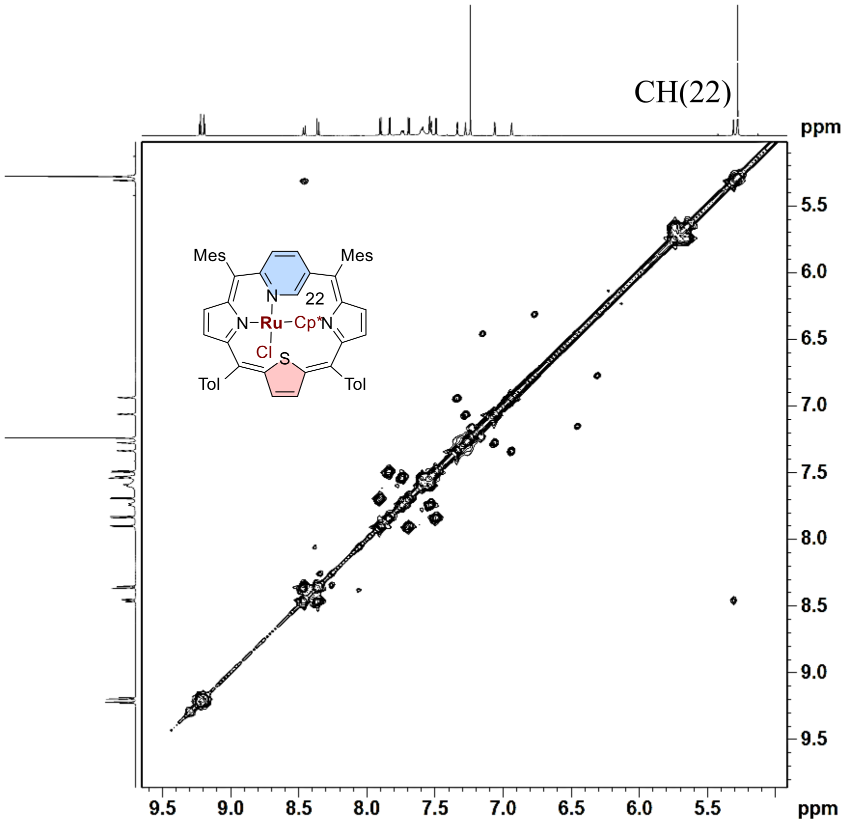

Figure S21. Part of the COSY spectrum of 14 (600 MHz, 300 K, $CDCl_3$).

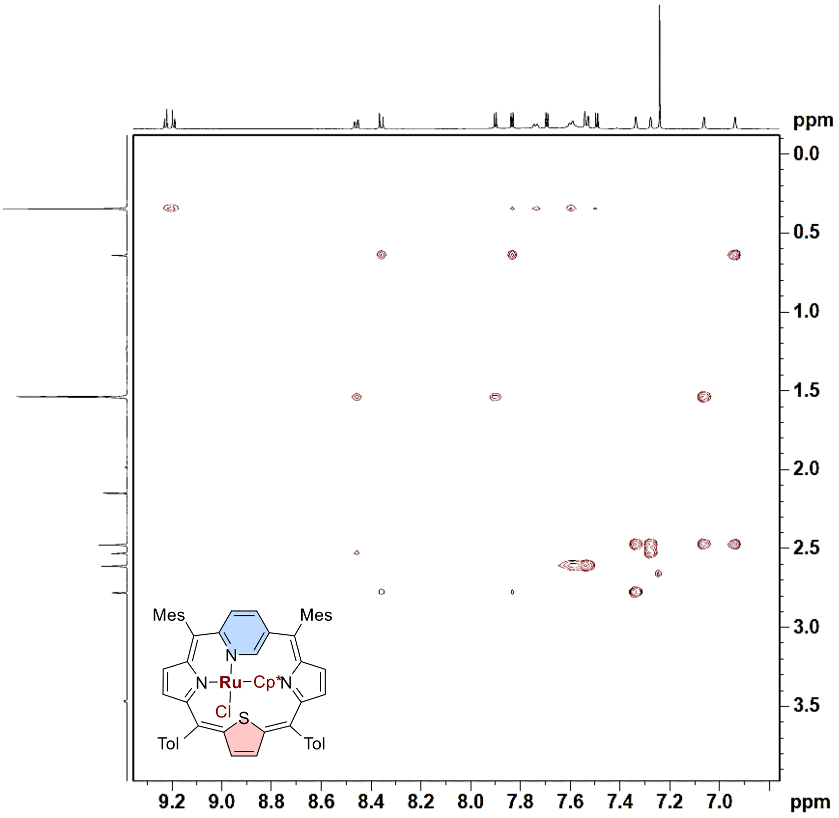

Figure S22. Part of the NOESY spectrum of 14 (600 MHz, 300 K, CDCl$_3$).

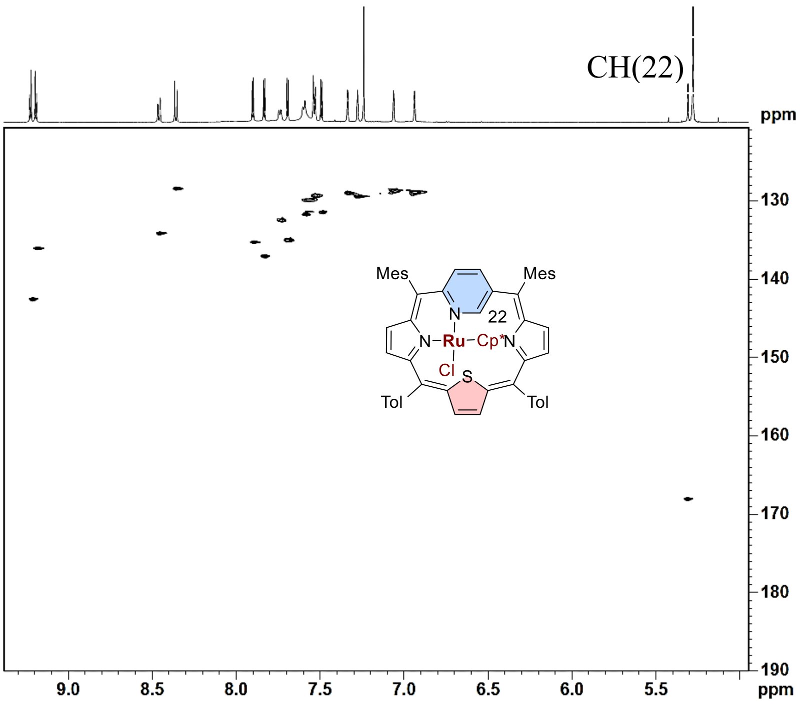

Figure S23. Part of the HSQC spectrum of 14 (600 MHz, 300 K, CDCl$_3$).

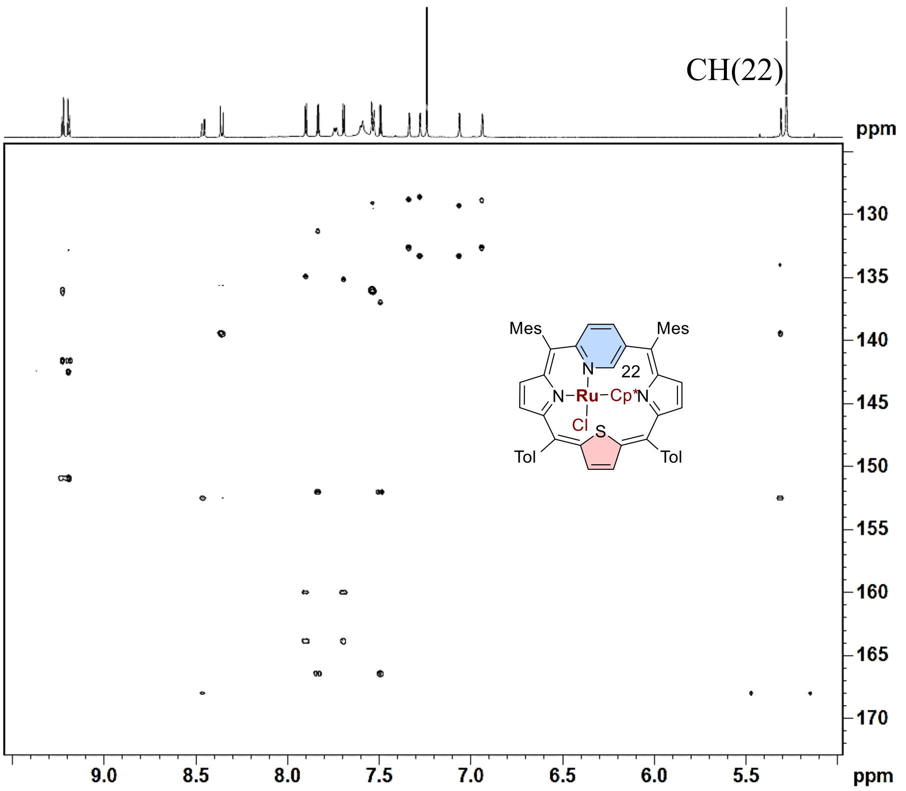

Figure S24. Part of the HMBC spectrum of 14 (600 MHz, 300 K, CDCl$_3$).

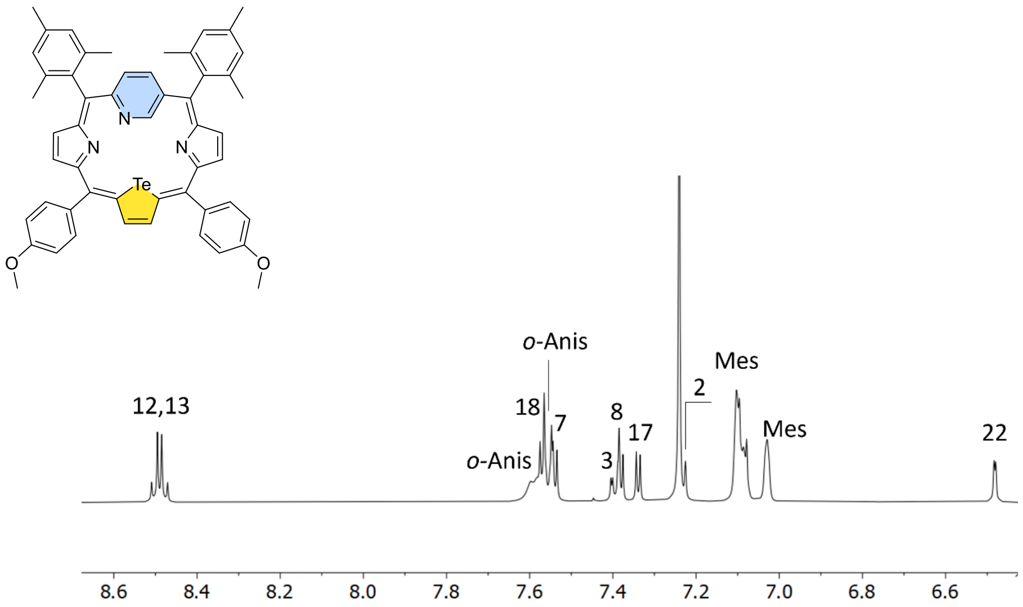

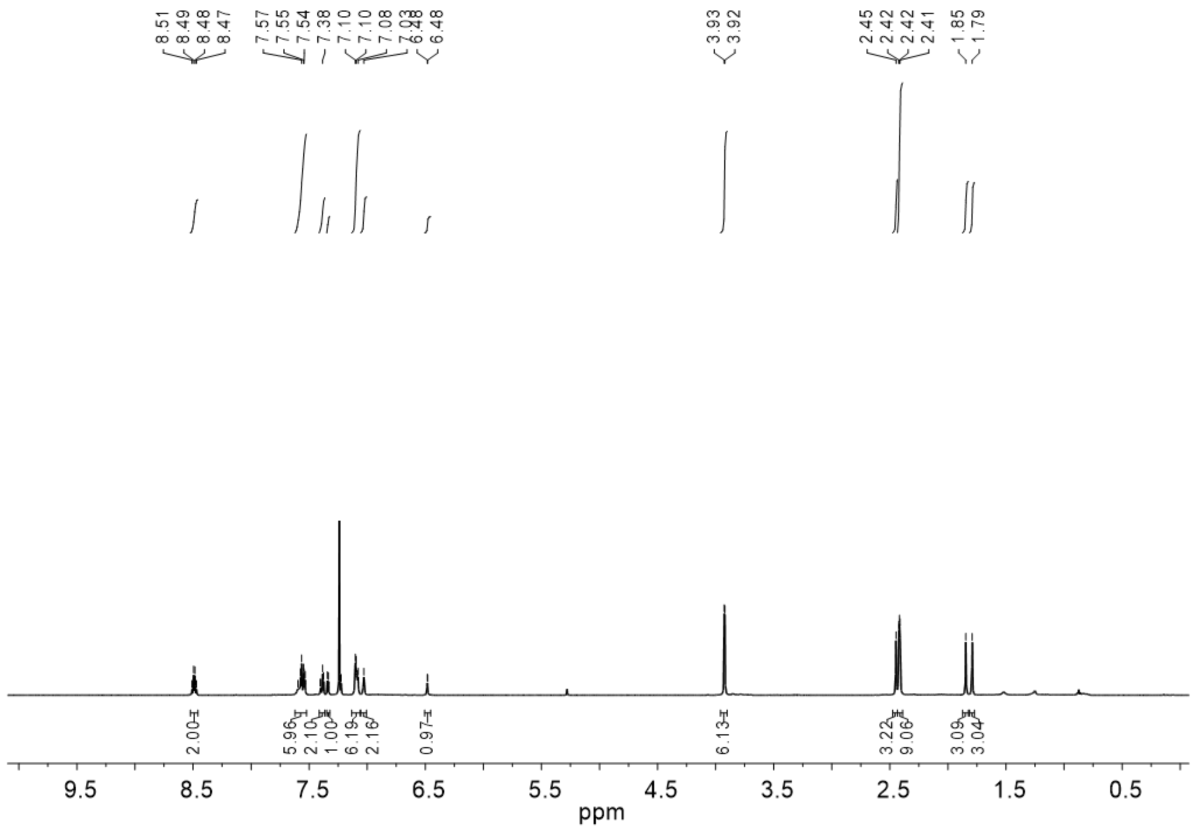

Figure S25. Signals assignment - part of the ^{1}H NMR spectrum of 6 (top; 600 MHz, 300 K, $CDCl_3$); the ^{1}H NMR spectrum of 6 (bottom; 600 MHz, 300 K, $CDCl_3$)

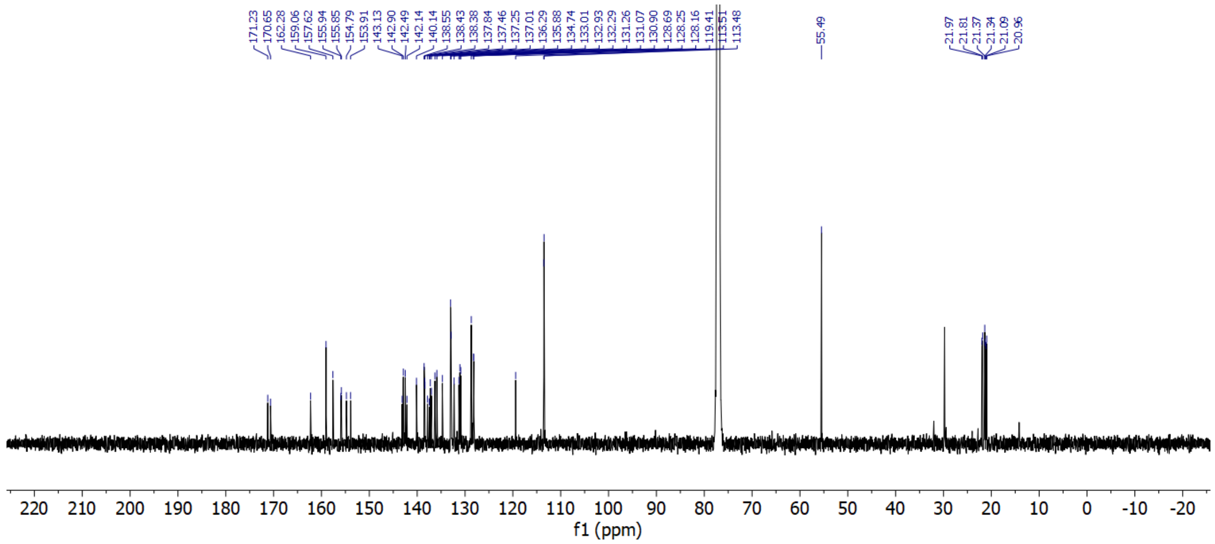

Figure S26.The ^{13}C NMR spectrum of 6 (600 MHz, 300 K, $CDCl_3$).

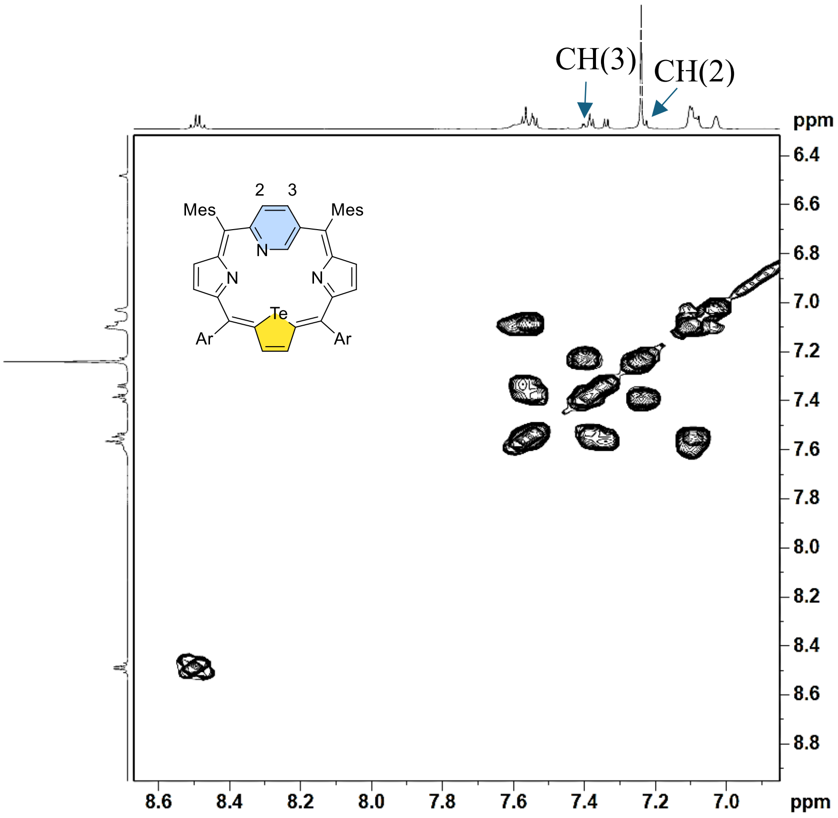

Figure S27. Part of COSY spectrum of 6 (600 MHz, 300 K, $CDCl_3$).

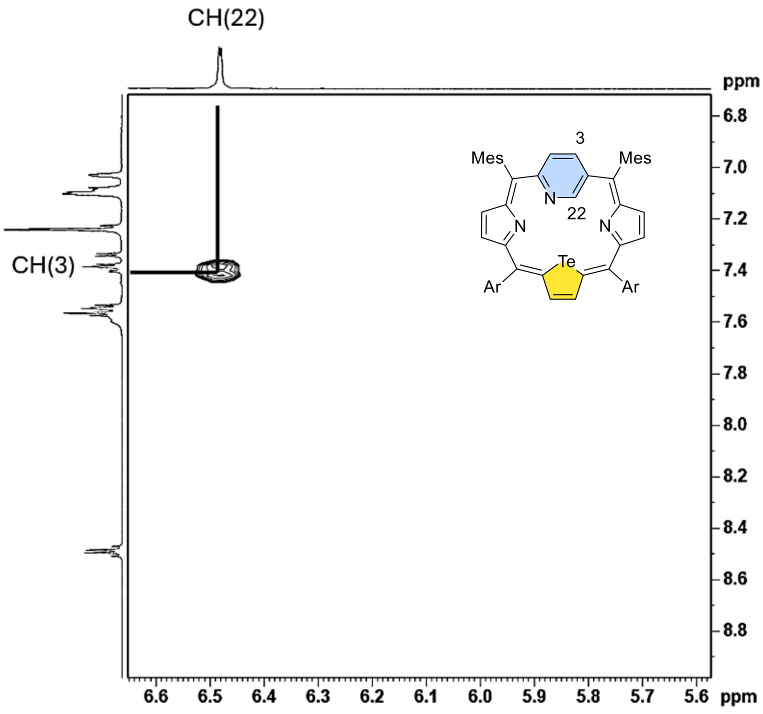

Figure S28. Part of COSY spectrum of 6 (600 MHz, 300 K, $CDCl_3$).

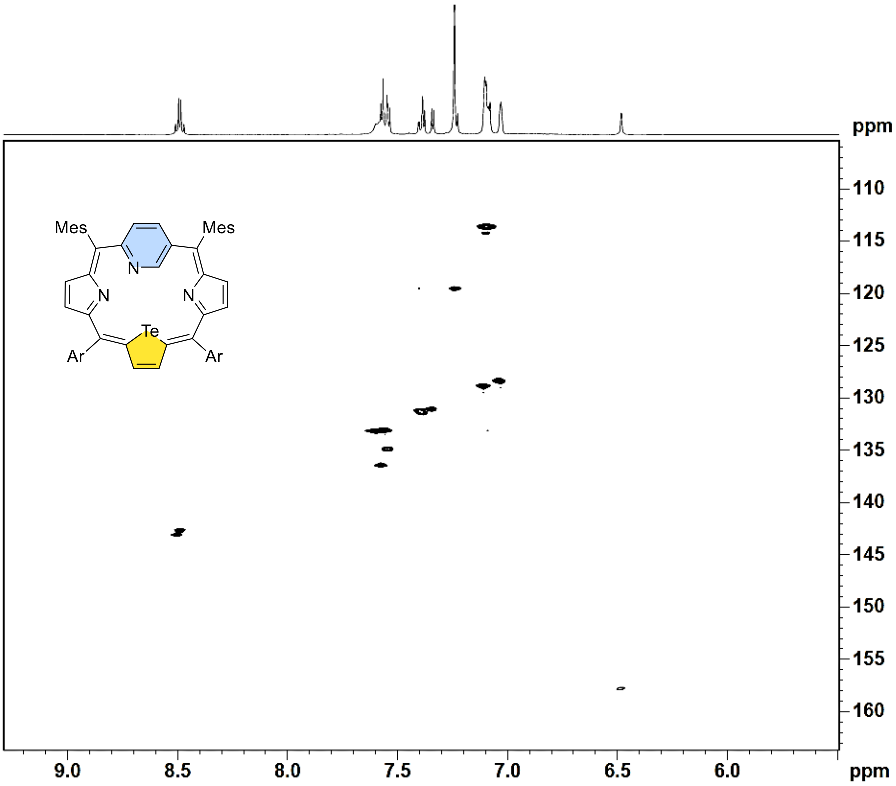

Figure S29. Part of the HSQC spectrum of 6 (600 MHz, 300 K, CDCl₃).

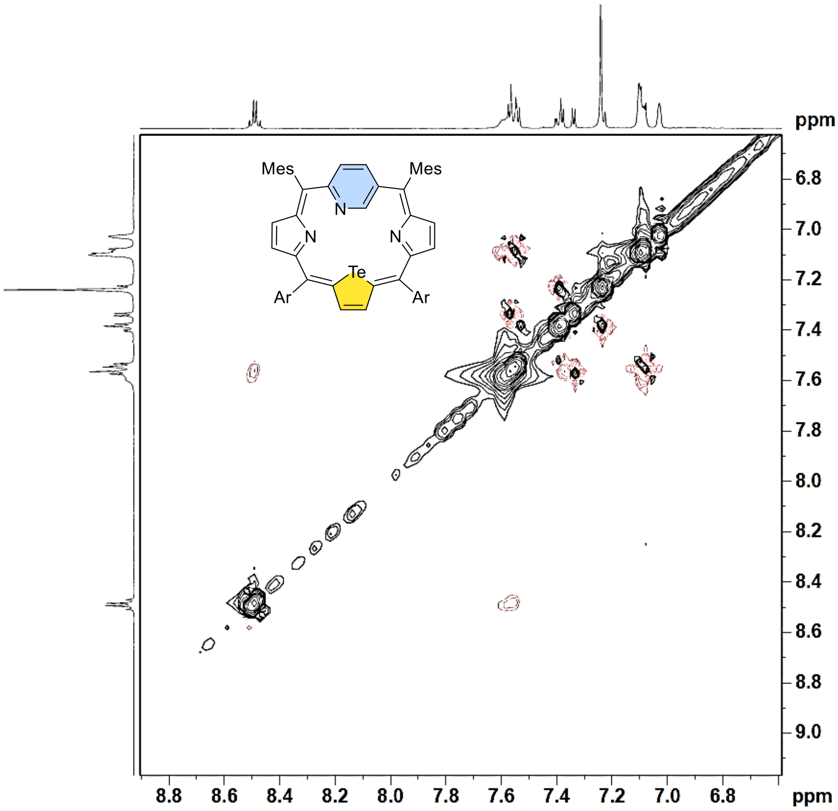

Figure S30. Part of the NOESY spectrum of 6 (600 MHz, 300 K, CDCl₃).

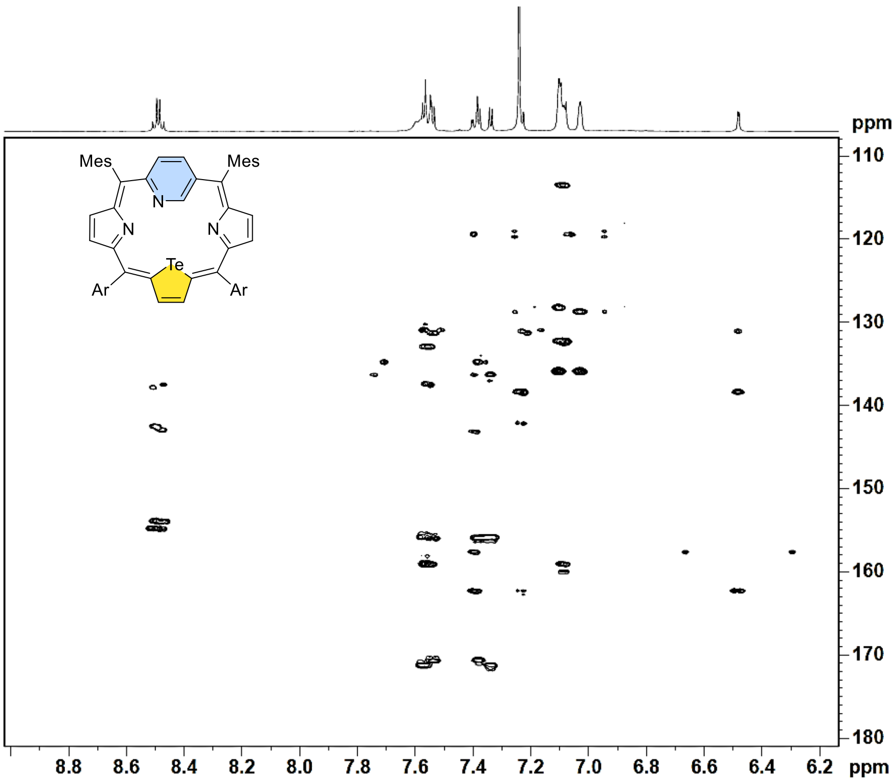

Figure S31. Part of the HMBC spectrum of 6 (600 MHz, 300 K, $CDCl_3$).

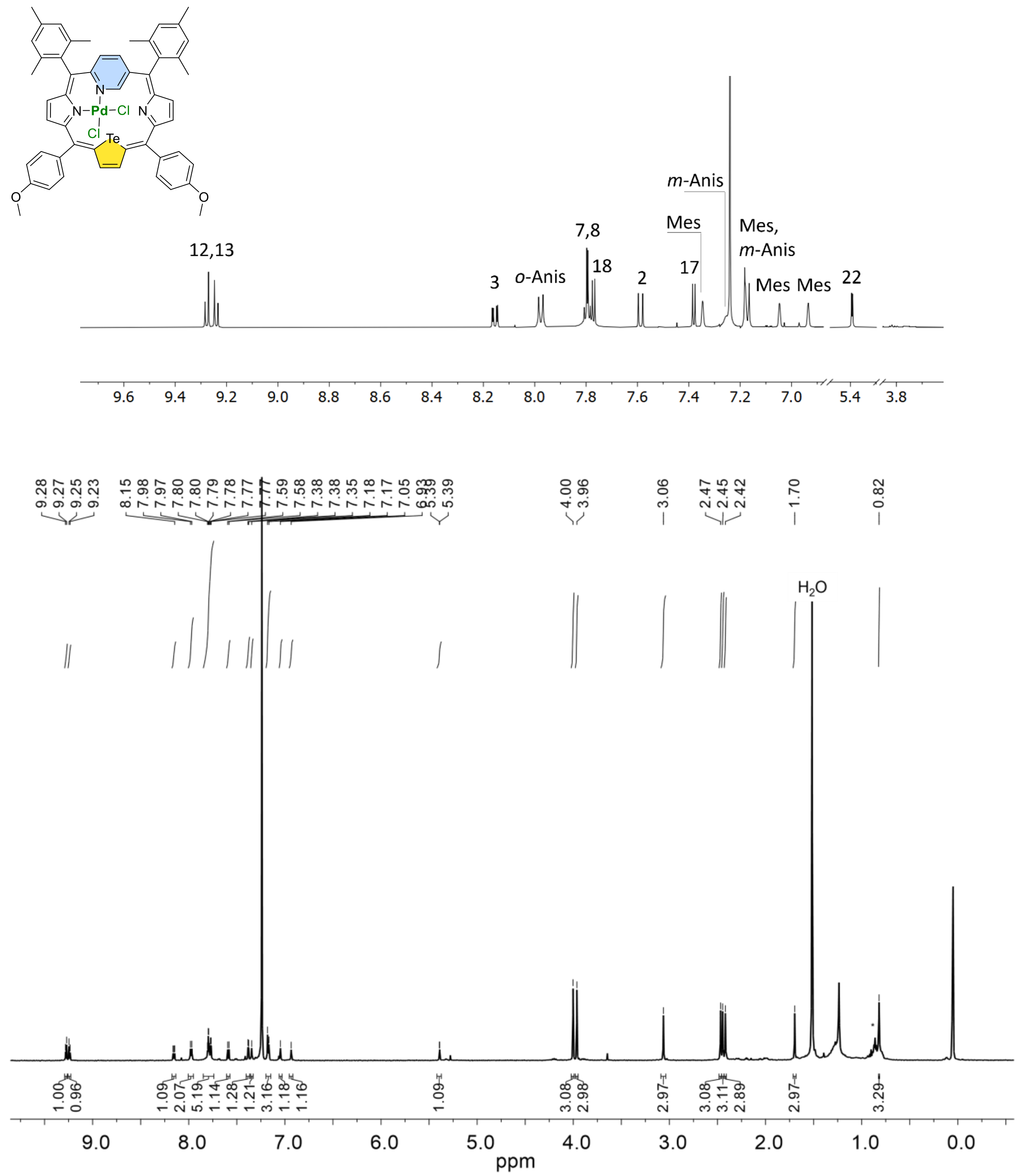

Figure S32. Signals assignment - part of the ^{1}H NMR spectrum of 13 (top; 600 MHz, 300 K, $CDCl_3$), the ^{1}H NMR spectrum of 13 (bottom; 600 MHz, 300 K, $CDCl_3$).

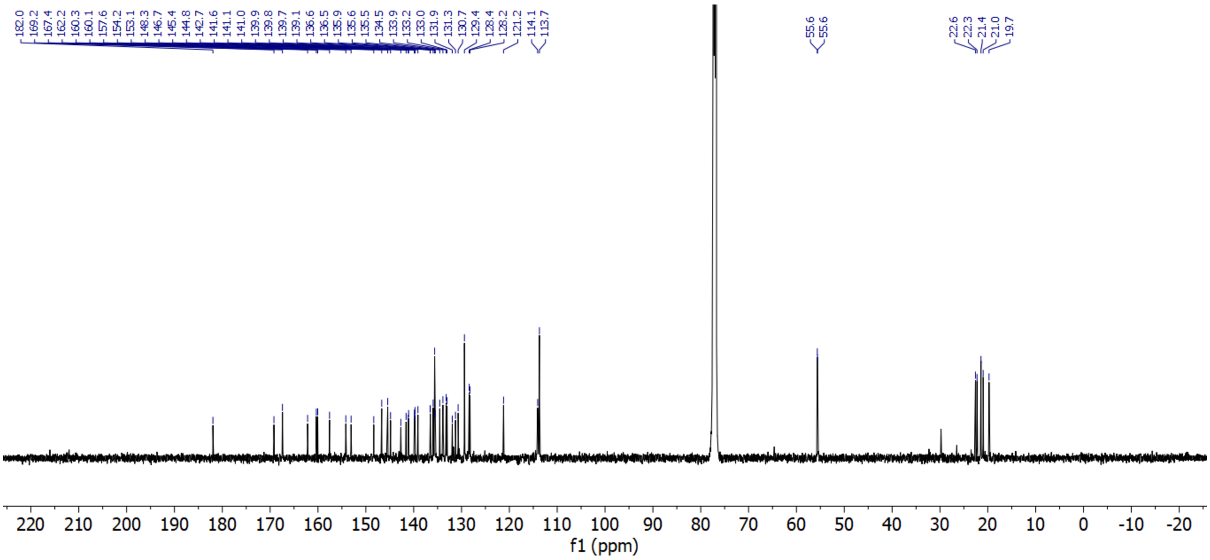

Figure S33. The ^{13}C NMR spectrum of 13 (600 MHz, 300 K, $CDCl_3$).

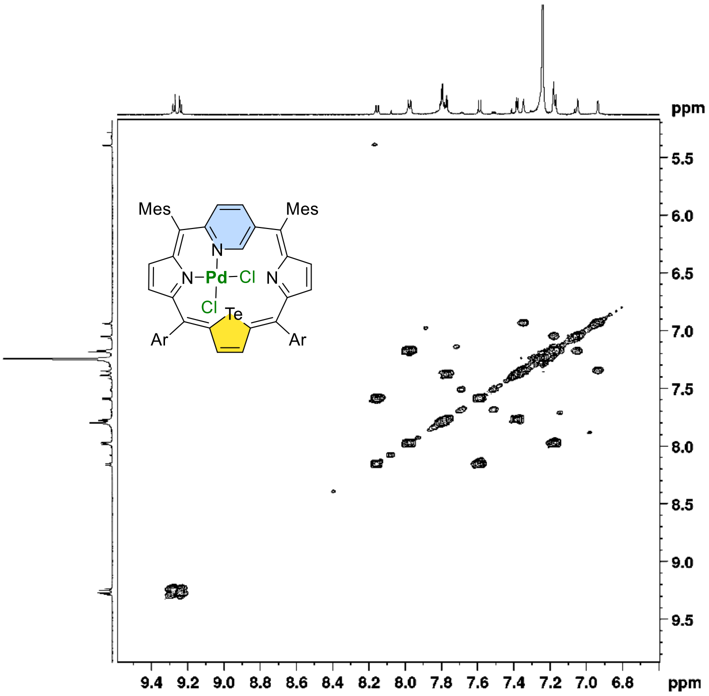

Figure S34. Part of the COSY spectrum of 13 (600 MHz, 300 K, $CDCl_3$).

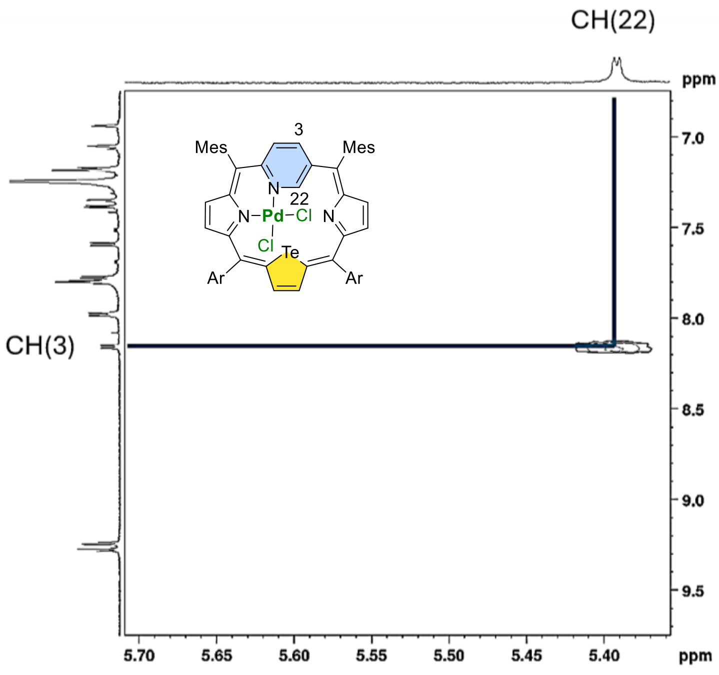

Figure S35. Part of the COSY spectrum of 13 (600 MHz, 300 K, $CDCl_3$).

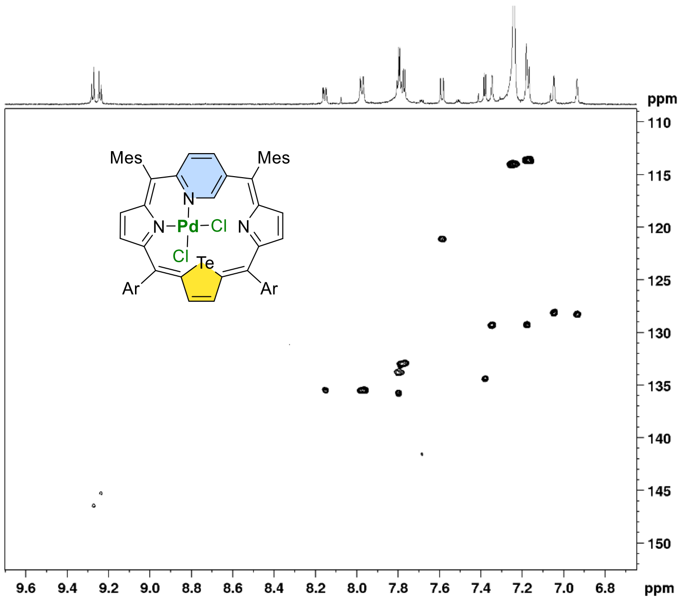

Figure S36. Part of the HSQC spectrum of 13 (600 MHz, 300 K, CDCl3).

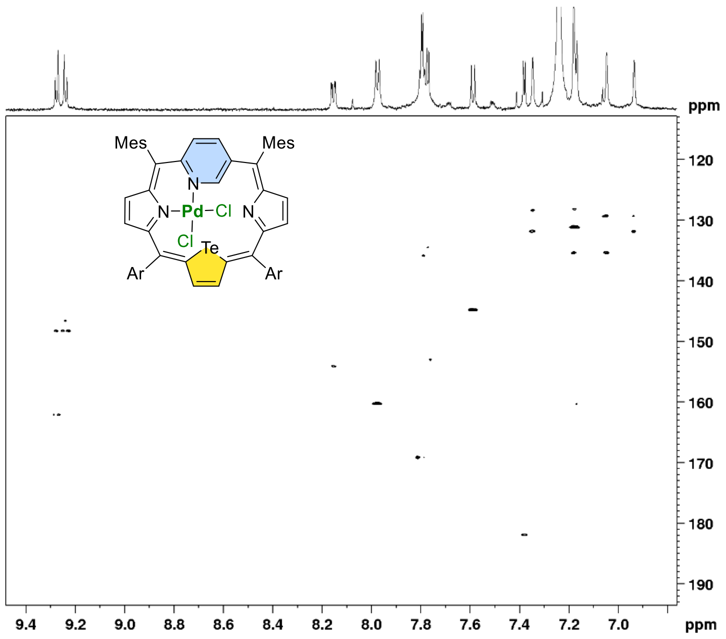

Figure S37. Part of the HMBC spectrum of 13 (600 MHz, 300 K, $CDCl_3$).

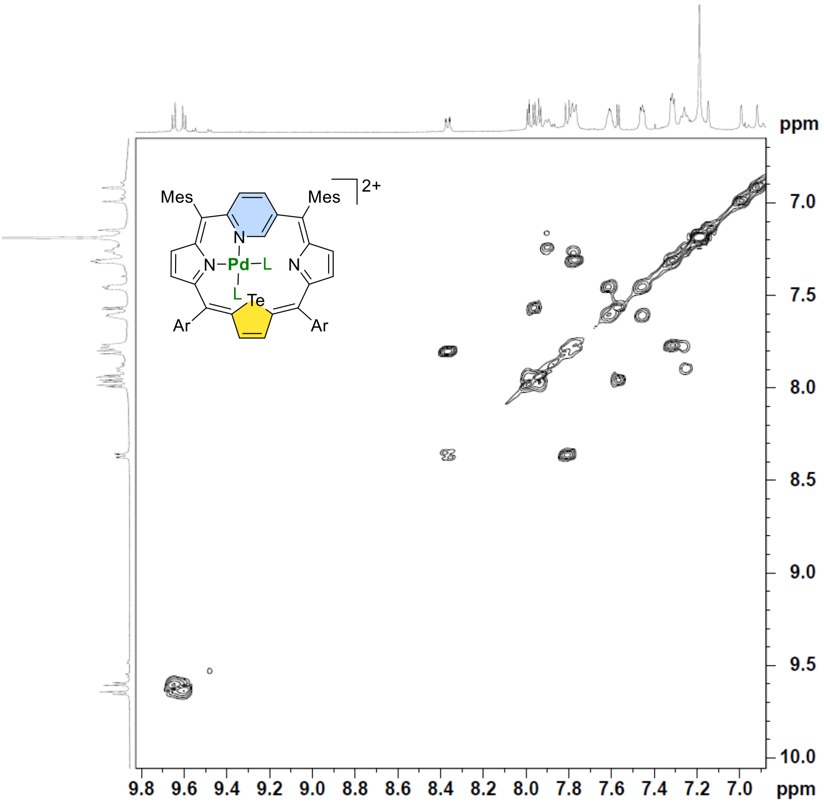

Figure S38. Part of the COSY spectrum of 13-2+ (600 MHz, 250 K, $CDCl_3$).

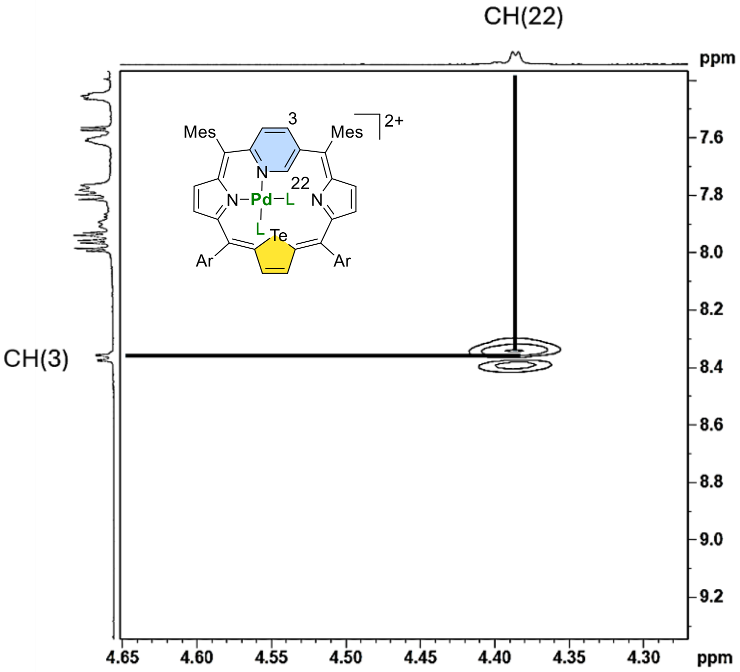

Figure S39. Part of the COSY spectrum of 13-2+ (600 MHz, 250 K, $CDCl_3$).

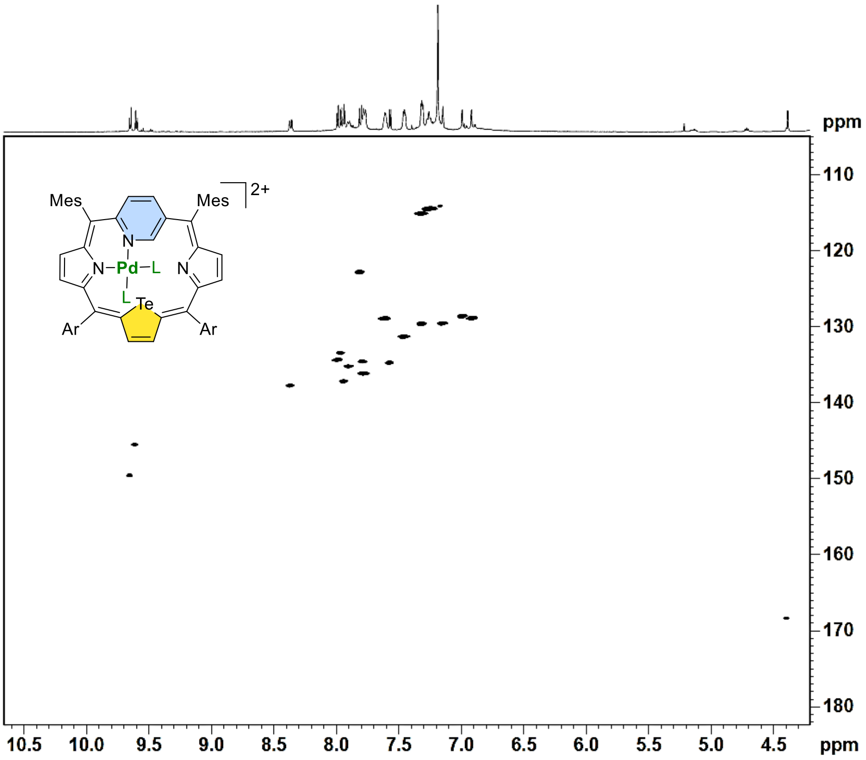

Figure S40. Part of the HSQC spectrum of 13-2+ (600 MHz, 250 K, $CDCl_3$).

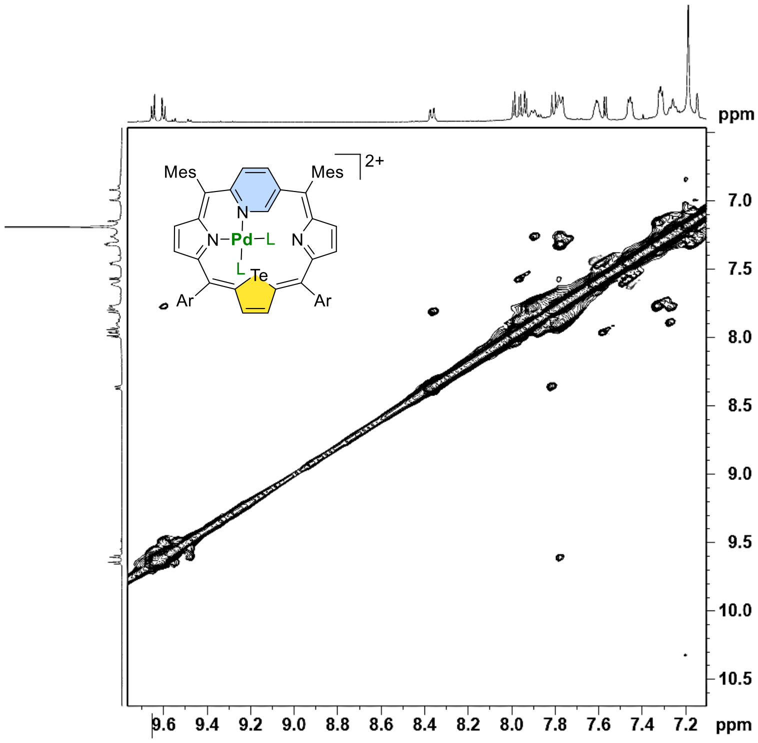

Figure S41. Part of the NOESY spectrum of 13-2+ (600 MHz, 250 K, $CDCl_3$).

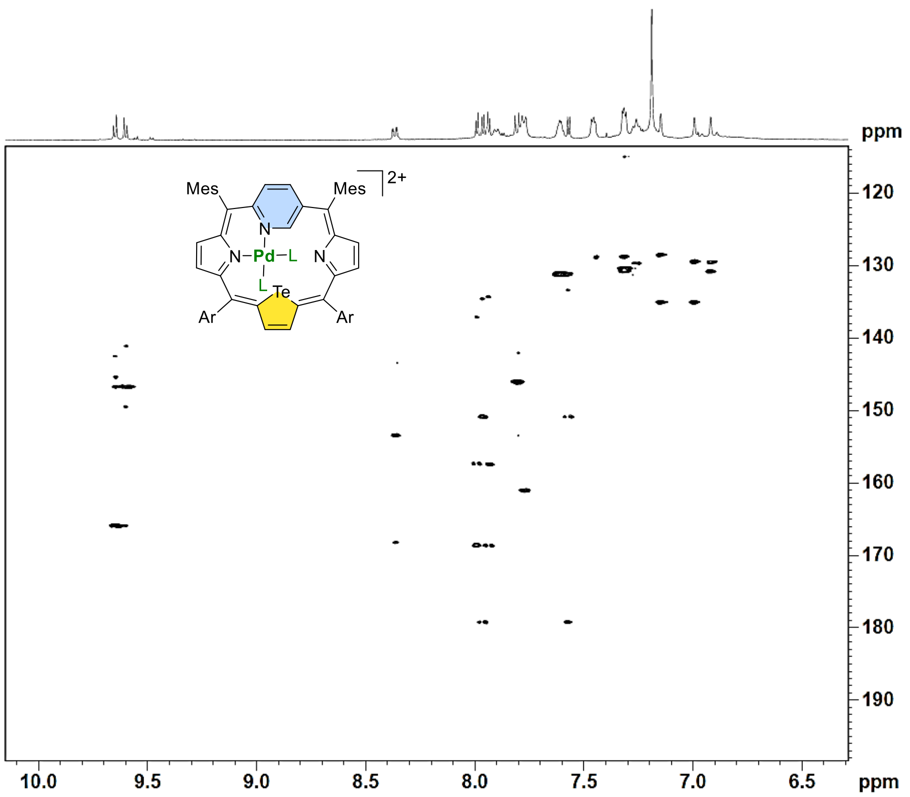

Figure S42. Part of the HMBC spectrum of 13-2+ (600 MHz, 250 K, CDCl₃).

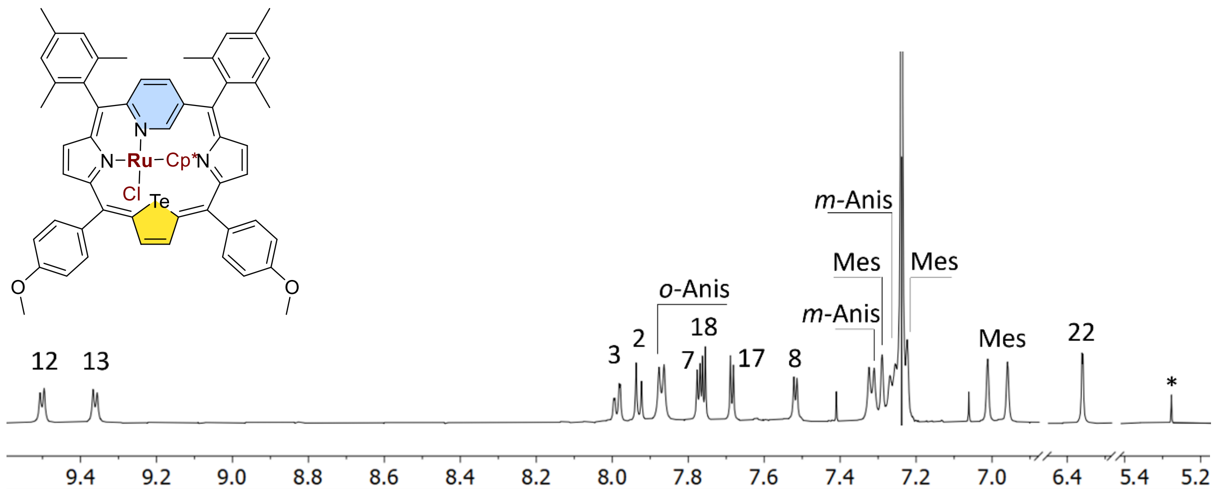

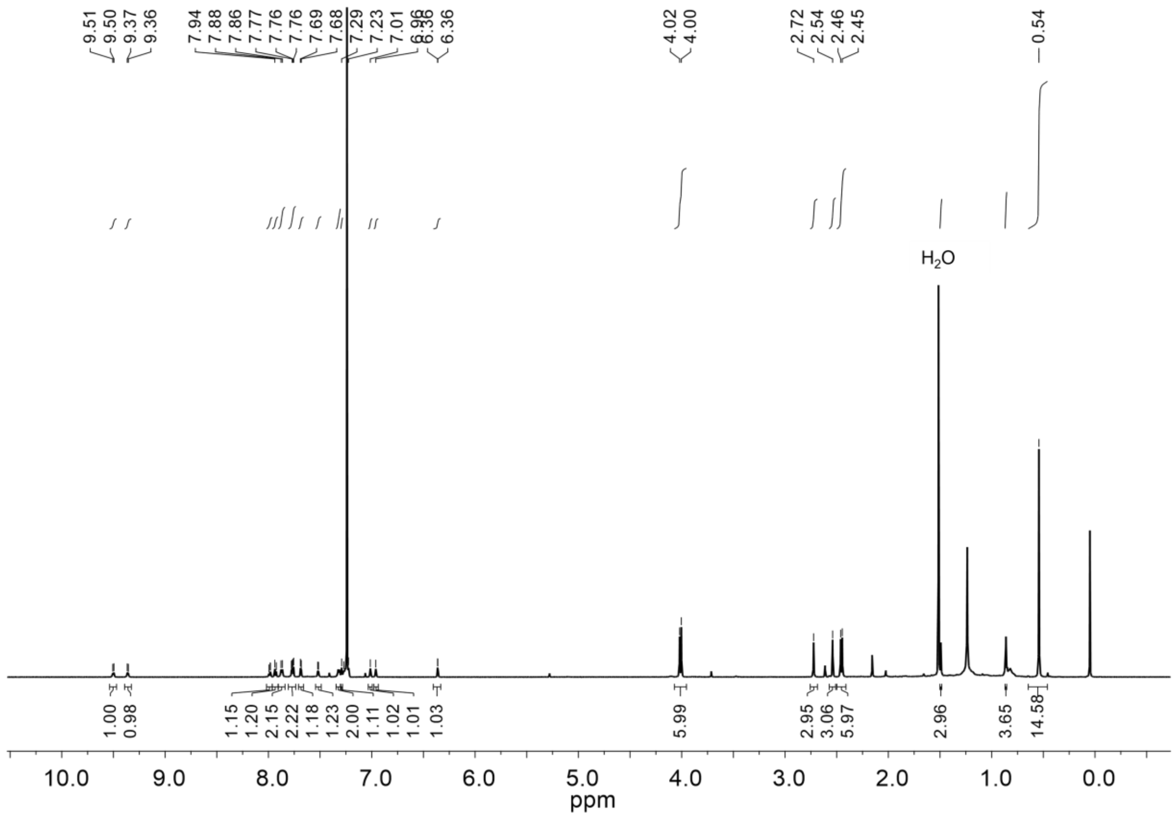

Figure S43. Signals assignment - part of the ^1H NMR spectrum of 15 (top; 600 MHz, 300 K, $CDCl_3$), the ^1H NMR spectrum of 15 (bottom; 600 MHz, 300 K, $CDCl_3$).

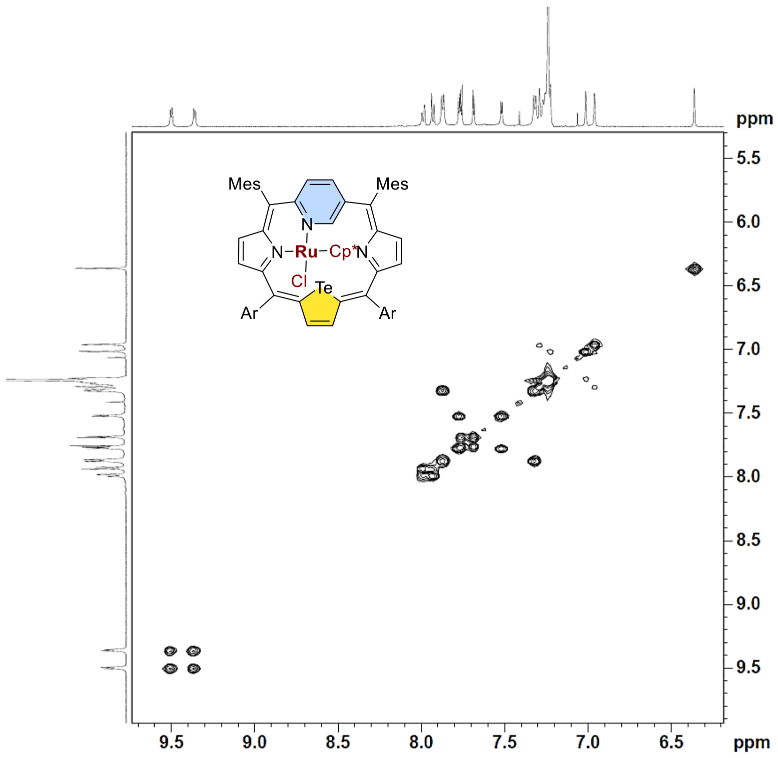

Figure S44. Part of the COSY spectrum of 15 (600 MHz, 300 K, $CDCl_3$).

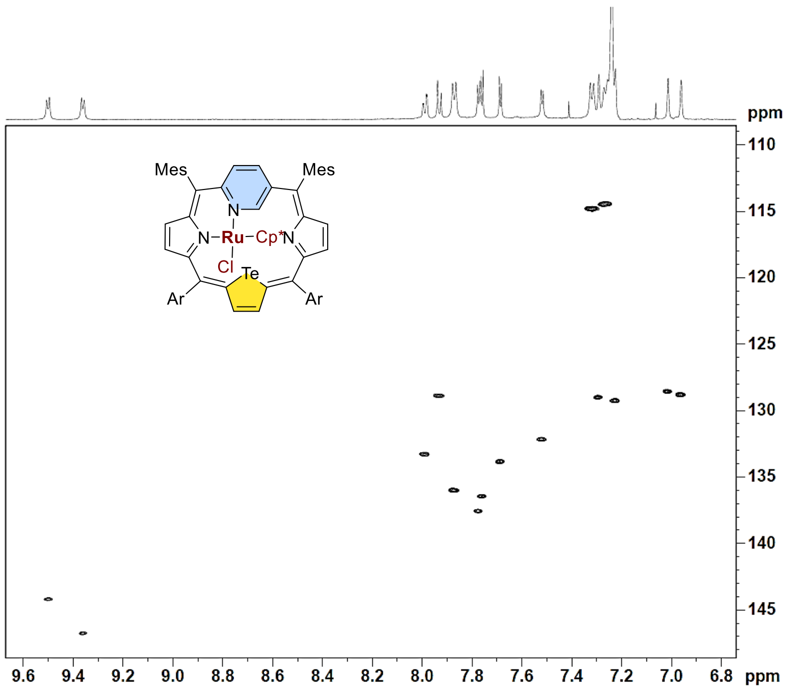

Figure S45. Part of the HSQC spectrum of 15 (600 MHz, 300 K, $CDCl_3$).

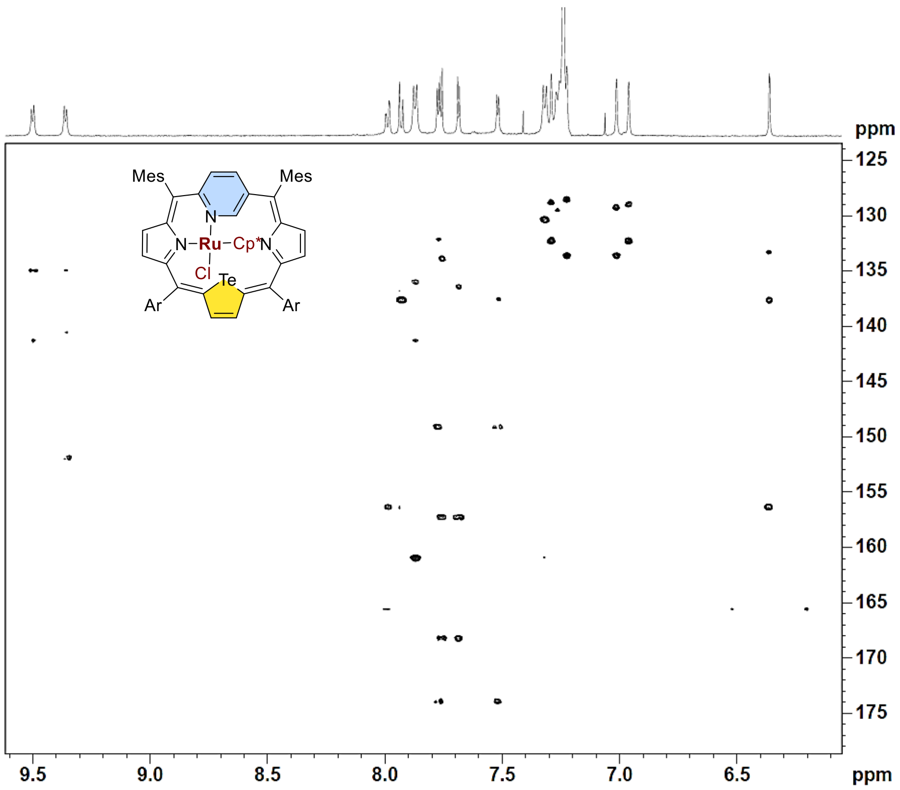

Figure S46. Part of the HMBC spectrum of 15 (600 MHz, 300 K, CDCl₃).

MS

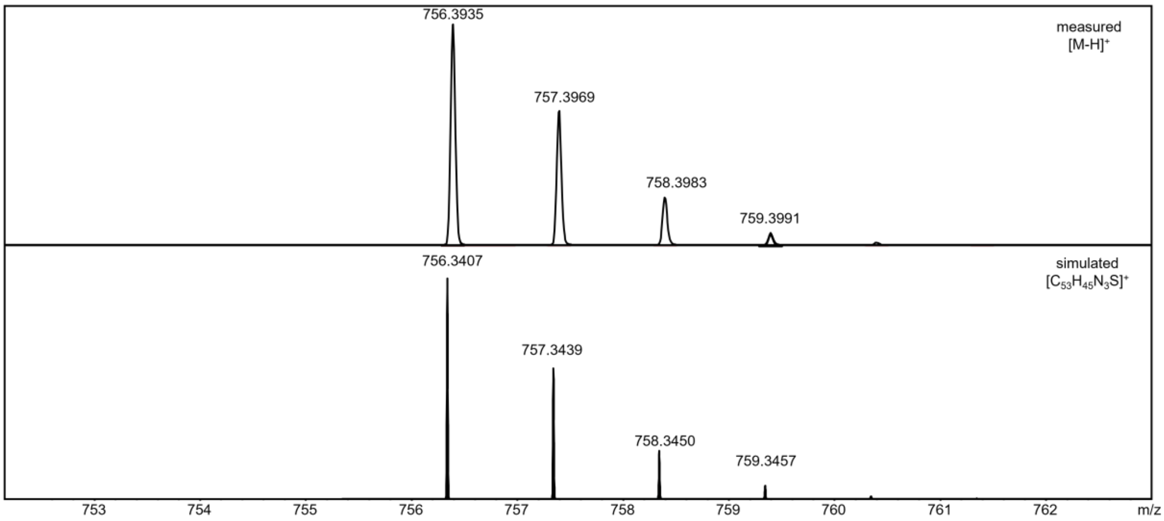

Figure S47. Selected region of the HRMS ESI (+MS) spectrum of 5.

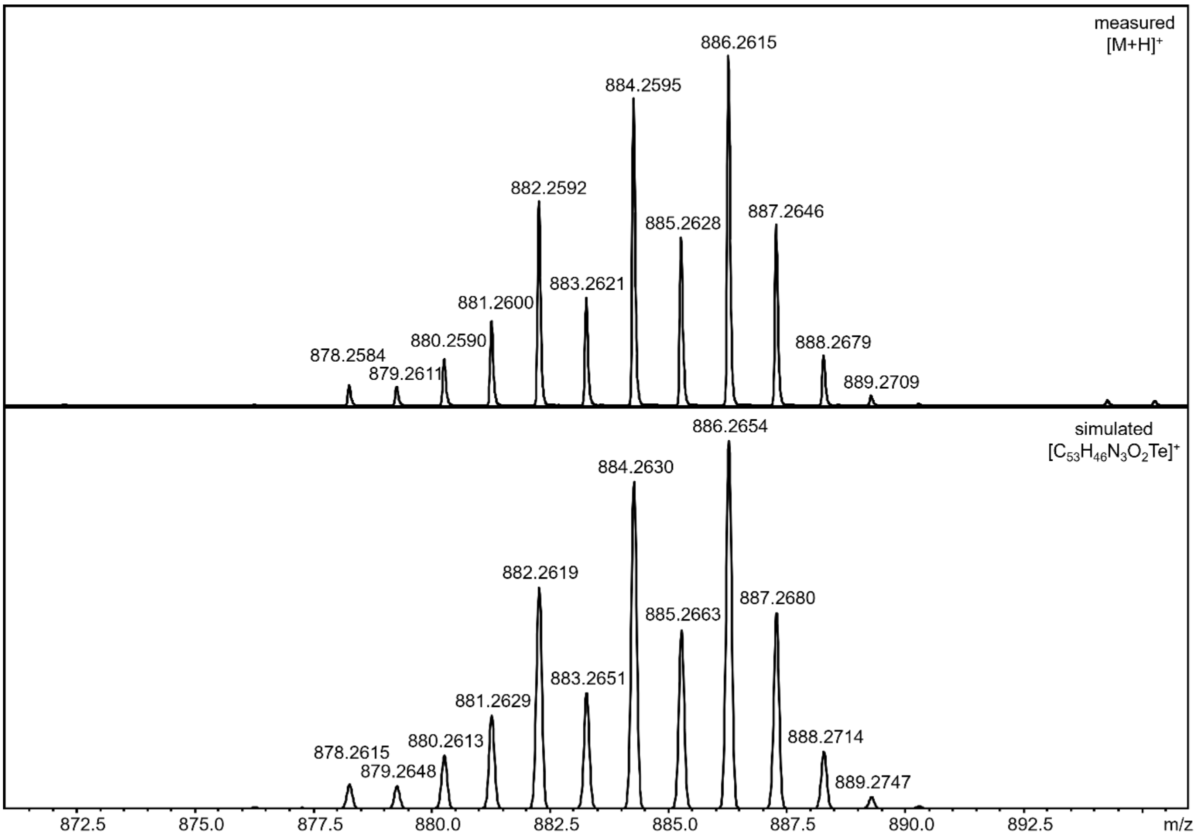

Figure S48. Selected region of the HRMS ESI (+MS) spectrum of 6.

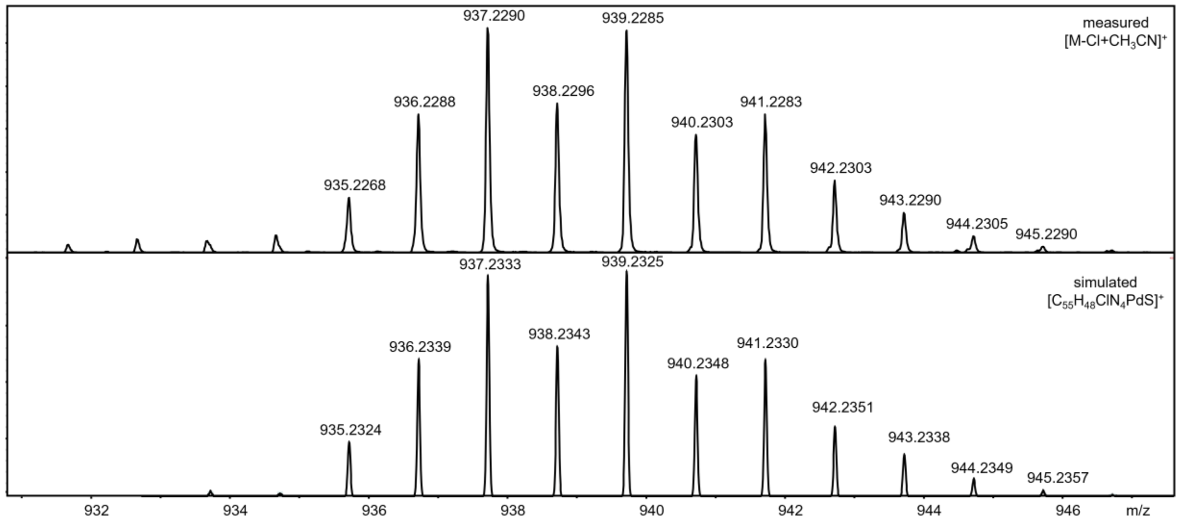

Figure S49. Selected region of the HRMS ESI (+MS) spectrum of 12.

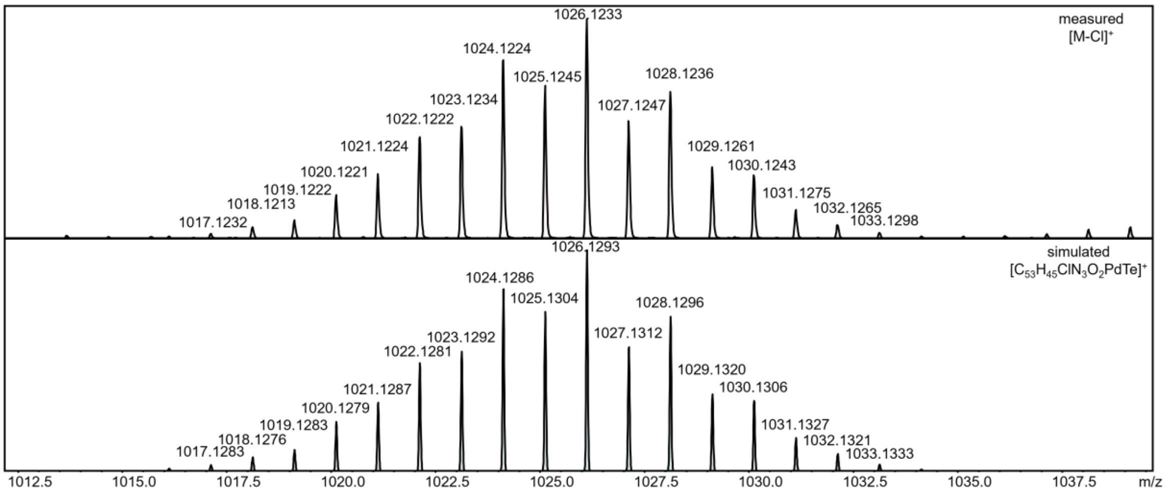

Figure S50. Selected region of the HRMS ESI (+MS) spectrum of 13.

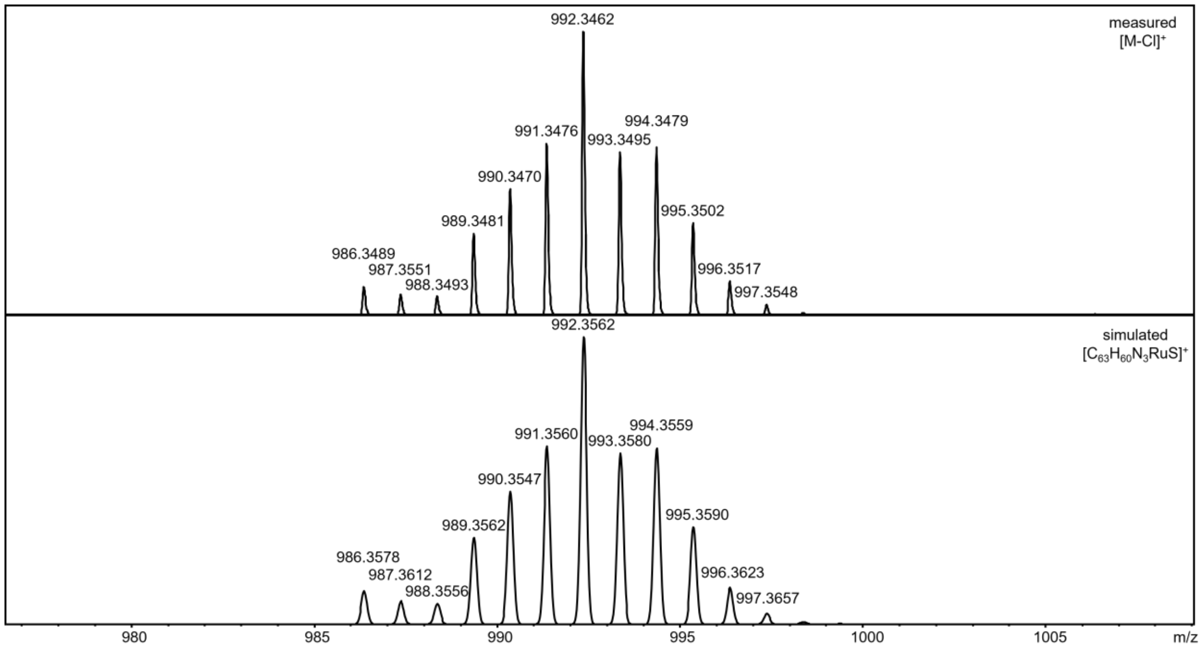

Figure S51. Selected region of the HRMS ESI (+MS) spectrum of 14.

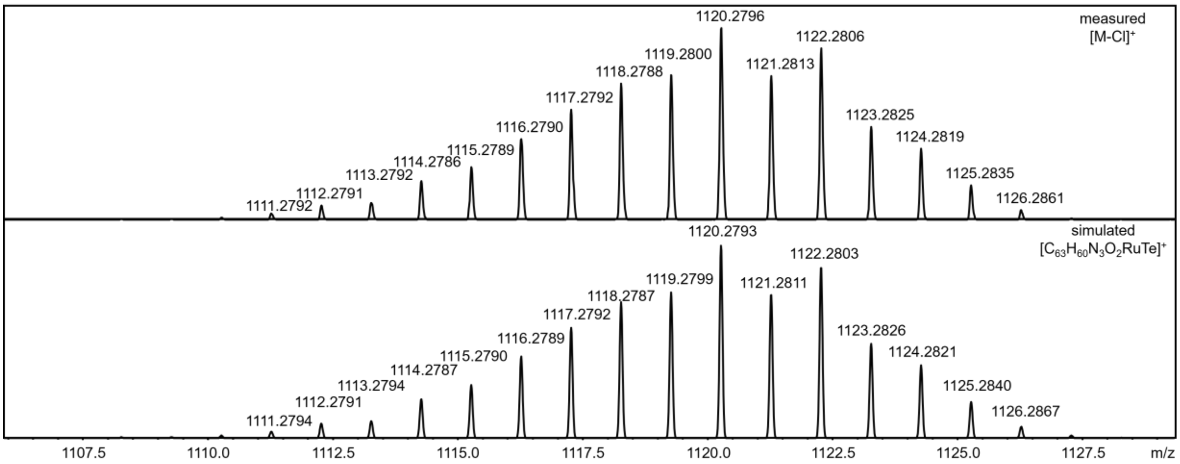

Figure S52. Selected region of the HRMS ESI (+MS) spectrum of 15.

DFT

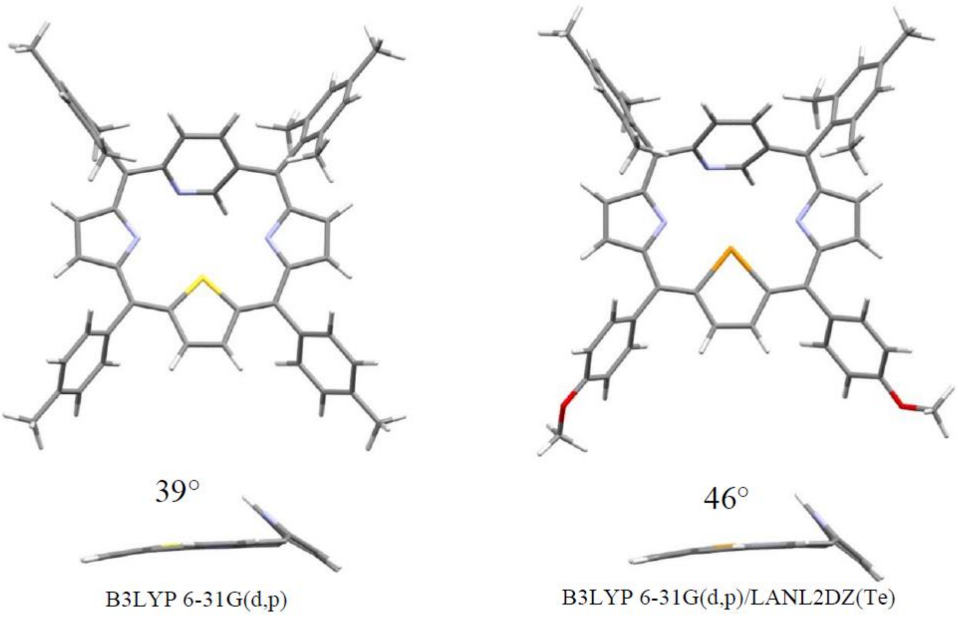

Figure S53. The DFT-optimised models of 5 (left) and 6 (right). For clarity, aryl groups were omitted from the side views (bottom).

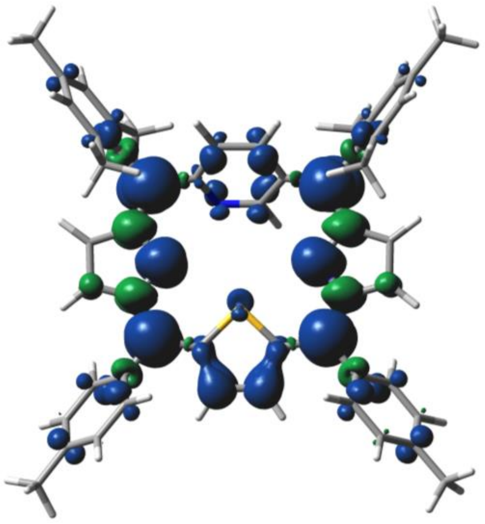

Figure S54. Spin density distribution for an optimized triplet model. Isovalue = 0.002.

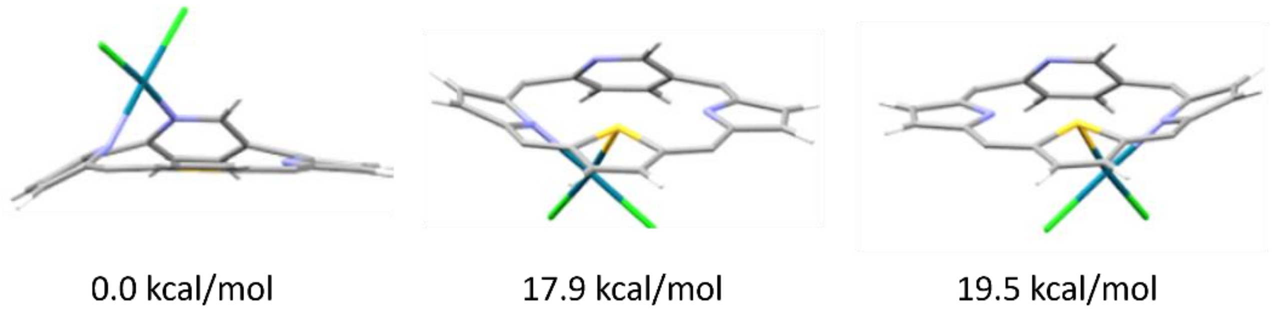

Figure S55. The geometries and relative energies of the three possible coordination modes of the side-on palladium(II) complex with 24-thia-*p*-pyriporphyrin were obtained by DFT optimisation. Aryl groups omitted for clarity.

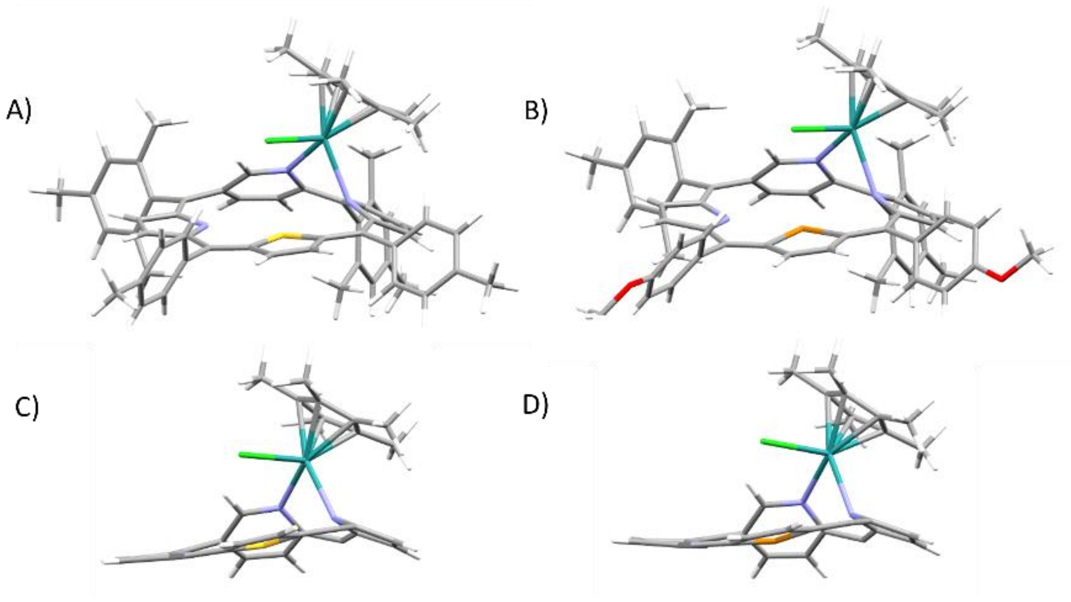

Figure S56. The DFT-optimized models (B3LYP 6-31G(d,p)/LANL2DZ(Te, Ru)) of 14 (A and C) and 15 (B and D). For clarity, aryl groups were omitted from the side views (C and D).

Investigate the effect of metalation on π-conjugation and ring current within macrocycles 5 and 12.

$EDDB_G(r)$ surfaces have been calculated to confirm delocalization of electrons within the macrocycle in **5** and **12**. It shows similarly efficient delocalization for **5** as for its Pd^{II} complex **12**. This is expected as the macrocycle conformation in both cases is similar in the energetic minimum. The difference between them lies in locking the conformation via Pd coordination, whereas the free ligand is more labile, as we have shown in our investigations, and its

aromaticity is a sum of all conformations' aromaticities. Thus, when investigating a ligand model with the pyridine ring more perpendicular to the meso plane of the macrocycle (the conformation that has been found during a dihedral-angle-altering scan), one can clearly see a lower level of electron delocalization density. This is the reason for which complex aromaticity, as judged from ^{1}H NMR spectra, is slightly more pronounced.

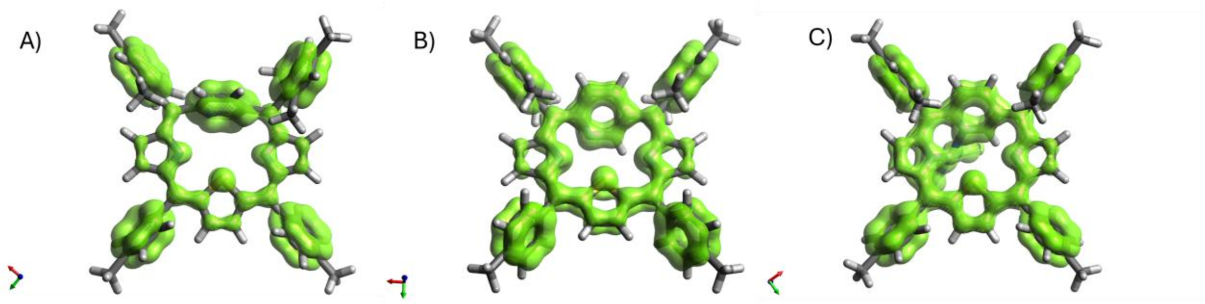

Figure S57. Electron density isosurface of A) and B) different conformers of 5, c) 12, drawn with an isovalue of 0.017. EDDB population analysis has been done at ωB97xD/def2sVP level using previously optimized DFT models.

NMR calculations

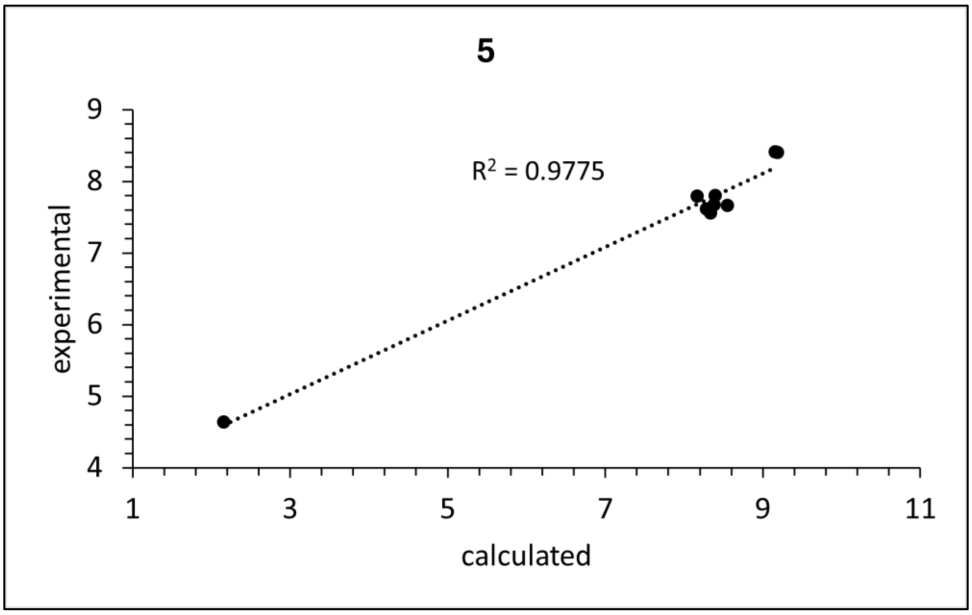

Figure S58. The correlation between calculated and experimental NMR values for 5.

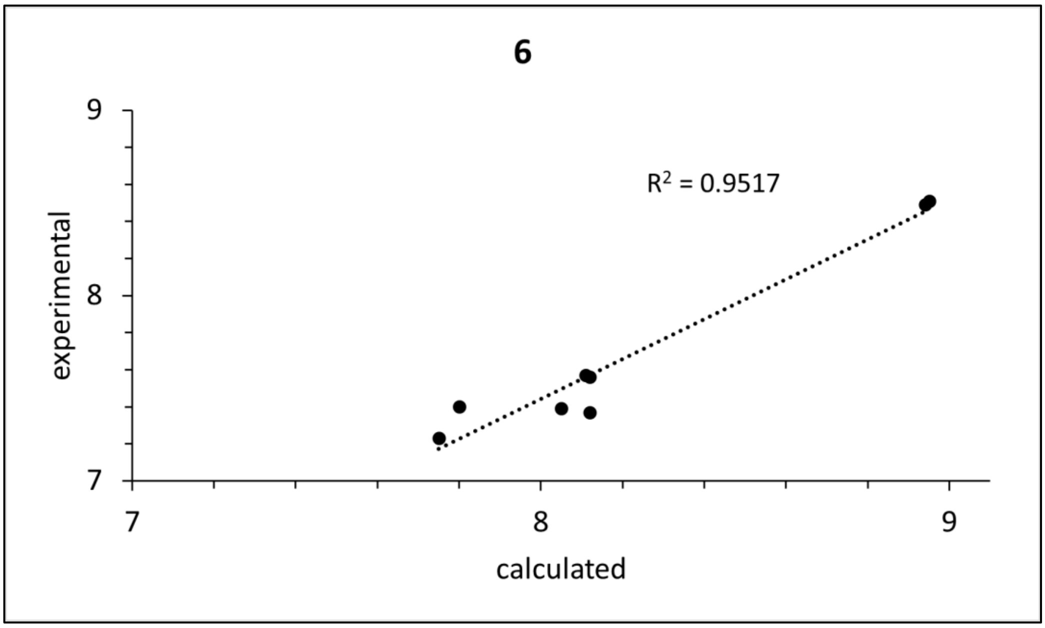

Figure S59. The correlation between calculated and experimental NMR values for 6.

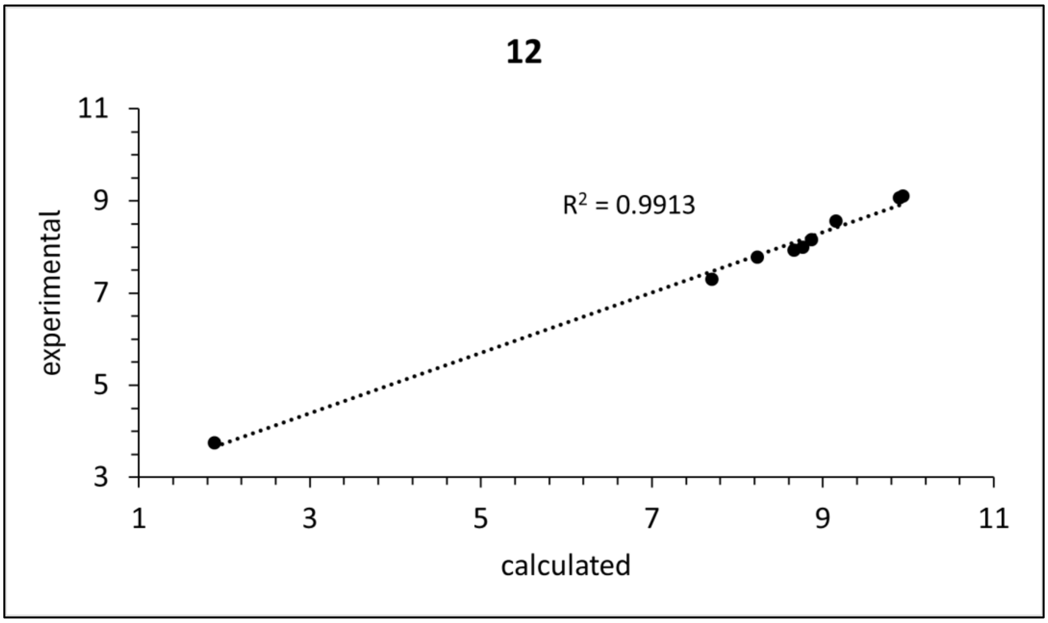

Figure S60. The correlation between calculated and experimental NMR values for 12.

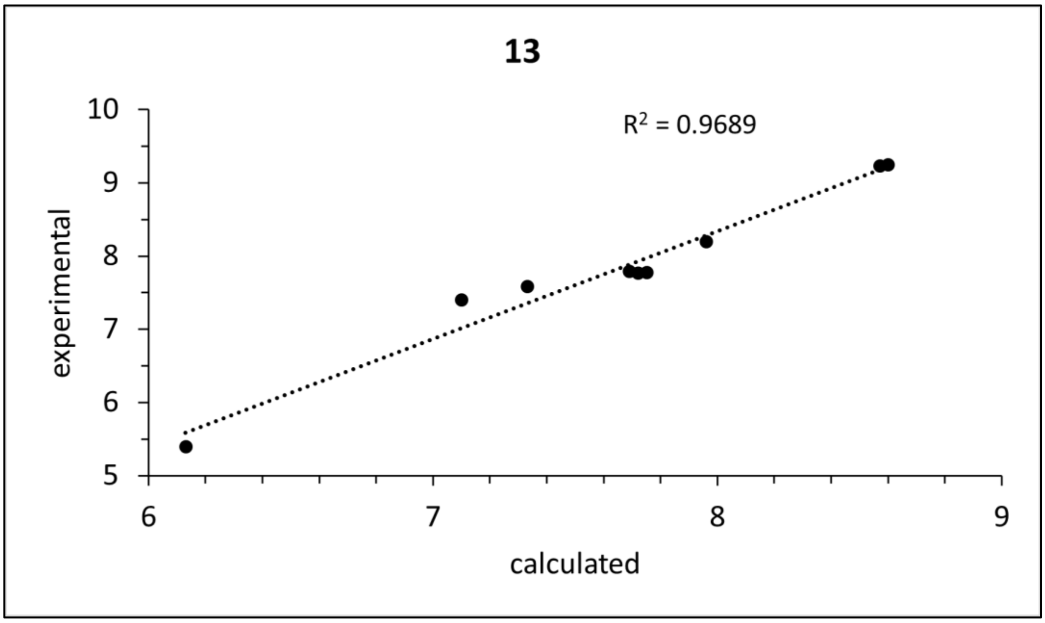

Figure 61. The correlation between calculated and experimental NMR values for 13.

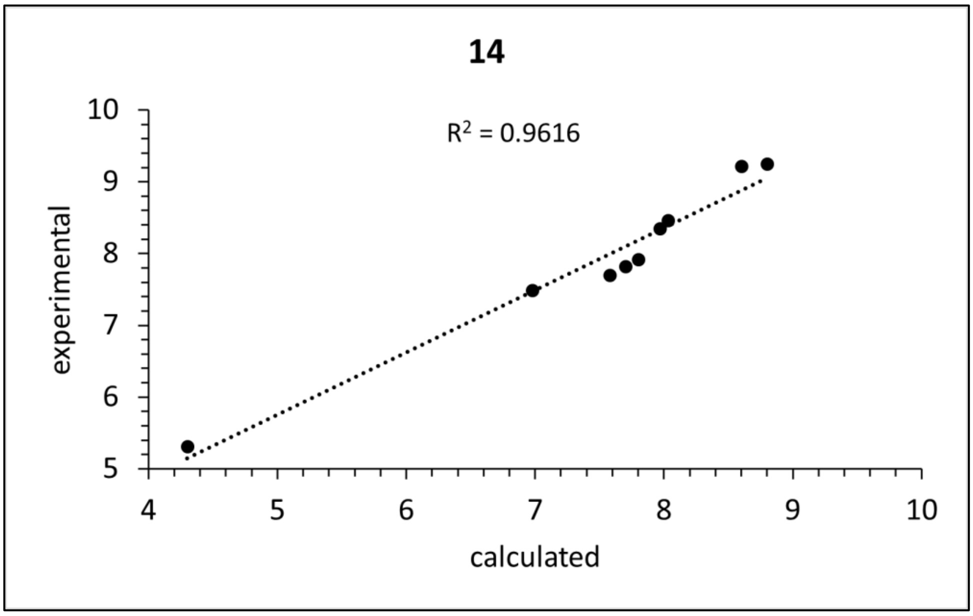

Figure S62. The correlation between calculated and experimental NMR values for 14.

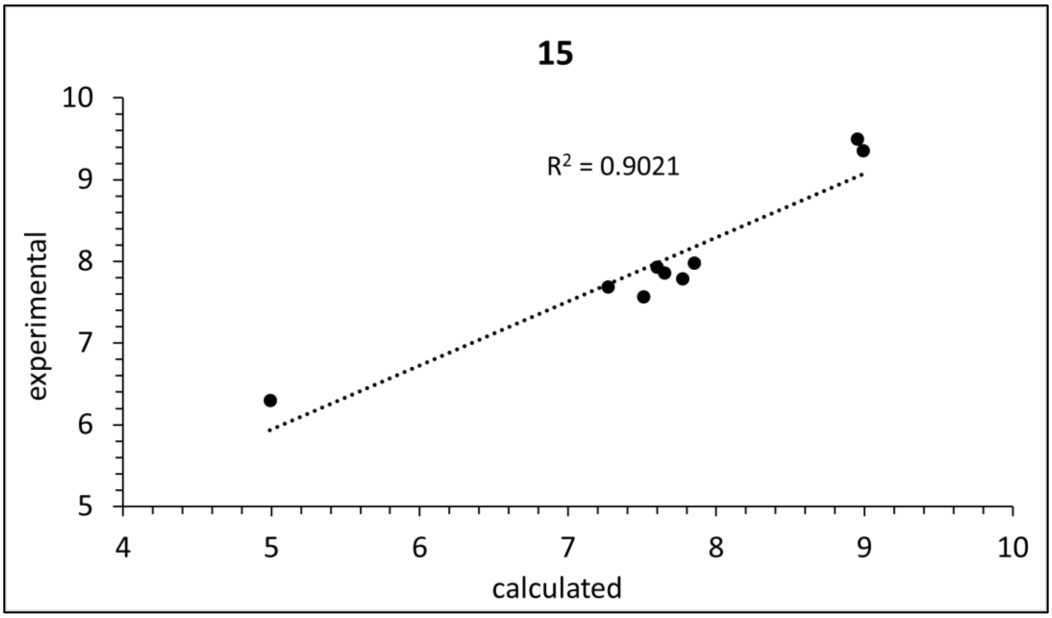

Figure S63.The correlation between calculated and experimental NMR values for 15.

Table 1. Coordinates for compound 5.

C	4.64414	-0.15794	-0.20064
C	4.64059	1.19718	-0.14727
C	3.22364	1.60065	-0.10147
C	2.71059	2.93751	-0.00841
C	1.34258	3.2262	-0.00951

C	0.76985	4.52727	0.1729
C	-0.59628	4.55253	0.15366
C	-1.21046	3.27224	-0.04152
C	-2.58804	3.03511	-0.06738
C	-3.15792	1.72074	-0.15609
C	-4.58869	1.37999	-0.18737
C	-4.65665	0.02372	-0.21546
C	-3.27559	-0.45642	-0.2402
C	-2.8371	-1.77921	-0.26372
C	2.75627	-1.87724	-0.29764
C	3.23755	-0.57397	-0.23225
N	2.42197	0.53731	-0.16018
S	0.04672	2.02042	-0.1777
N	-2.40741	0.61718	-0.19909
N	0.67487	-1.22809	-1.32653
C	1.33827	-2.10787	-0.53079
C	0.64037	-3.16358	0.10122
C	-0.63683	-1.21109	-1.29568
C	-1.43225	-2.05518	-0.4684
C	-0.74594	-3.12995	0.13752
H	-1.29716	-3.8738	0.70421
H	1.1885	-3.93235	0.63461
H	5.49731	-0.82	-0.26027
H	5.4934	1.86127	-0.14618
H	1.38289	5.40474	0.33271
H	-1.18062	5.45247	0.29503
H	-5.41068	2.08182	-0.18699
H	-5.5408	-0.59803	-0.25341
C	3.66108	-3.04033	-0.05606
C	3.96757	-3.91726	-1.12434
C	4.20552	-3.27901	1.22698
C	4.82139	-4.99867	-0.89352
C	3.41458	-3.68872	-2.51348

C	5.04326	-4.38514	1.41253
C	3.89363	-2.38668	2.40918
C	5.36662	-5.25563	0.36893
H	5.06905	-5.65686	-1.72334
H	5.45233	-4.56998	2.40322
C	6.2552	-6.45459	0.60009
C	3.69102	4.06132	0.09786
C	4.56909	4.14342	1.19177
C	3.76716	5.04963	-0.89773
C	5.49239	5.18489	1.2885
H	4.51659	3.39192	1.97373
C	4.6919	6.09006	-0.80116
H	3.10509	4.99055	-1.75623
C	5.55713	6.16151	0.29238
H	6.15822	5.23496	2.1451
H	4.73929	6.84134	-1.58404
H	6.27683	6.97121	0.36745
C	-3.52379	4.19955	0.0149
C	-4.39917	4.34059	1.10471
C	-3.55658	5.16938	-1.00082
C	-5.27922	5.42086	1.17712
H	-4.37887	3.60415	1.90235
C	-4.4381	6.24868	-0.92865
H	-2.89416	5.06628	-1.85494
C	-5.30206	6.37807	0.16052
H	-5.9439	5.51629	2.03074
H	-4.45243	6.98528	-1.72664
H	-5.98808	7.21797	0.21673
C	-3.78684	-2.9027	-0.00083
C	-4.31828	-3.10332	1.29343
C	-4.13741	-3.78453	-1.05075
C	-5.18919	-4.17828	1.50878
C	-3.95611	-2.20425	2.456

C	-5.02176	-4.8341	-0.79006
C	-3.5958	-3.5975	-2.45041
C	-5.56065	-5.05025	0.48284
H	-5.58391	-4.33796	2.50958
H	-5.29644	-5.50157	-1.60381
C	-6.53123	-6.18007	0.73177
H	5.65968	-7.35725	0.78537
H	6.88584	-6.65818	-0.27067
H	6.90559	-6.30944	1.46723
H	4.16379	-2.88296	3.34498
H	4.44833	-1.44343	2.36103
H	2.83208	-2.1267	2.45417
H	2.33849	-3.88994	-2.55761
H	3.55448	-2.65329	-2.83916
H	3.90488	-4.34447	-3.23743
H	-4.08139	-4.28431	-3.14805
H	-3.75769	-2.5764	-2.81118
H	-2.51717	-3.78229	-2.49544
H	-2.88436	-1.98727	2.48642
H	-4.472	-1.23984	2.39533
H	-4.23658	-2.67151	3.40356
H	-6.554	-6.46231	1.78806
H	-7.55135	-5.89326	0.44762
H	-6.27142	-7.06704	0.14587
H	-1.13018	-0.51716	-1.96778

Table 2. Coordinates for compound 6.

C	-0.60594	-4.73506	-0.23265
C	0.74977	-4.69534	-0.16082
C	1.12613	-3.27409	-0.16256
C	2.43733	-2.68819	-0.04467

C	2.57379	-1.30703	-0.06069
C	3.78447	-0.56136	0.13122
C	3.73118	0.80981	0.08669
C	2.46476	1.44438	-0.14433
C	2.22739	2.81049	-0.19841
C	0.87911	3.30491	-0.33016
C	0.41532	4.69915	-0.34787
C	-0.94304	4.65598	-0.38716
C	-1.31717	3.24297	-0.42589
C	-2.58911	2.70266	-0.37416
C	-2.3729	-2.90063	-0.32502
C	-1.06609	-3.3461	-0.31457
N	0.03897	-2.51343	-0.28019
Te	0.93459	-0.00584	-0.31709
N	-0.16282	2.47691	-0.40953
N	-1.97118	-0.79919	-1.45468
C	-2.68706	-1.49102	-0.53963
C	-3.66462	-0.84114	0.23983
C	-2.03423	0.51675	-1.45058
C	-2.79176	1.27367	-0.52738
C	-3.70788	0.54427	0.25214
H	-4.38677	1.06571	0.9164
H	-4.30866	-1.42038	0.88894
H	-1.24434	-5.60527	-0.25772
H	1.43125	-5.53081	-0.11855
H	4.71946	-1.07195	0.33276
H	4.62467	1.40217	0.24953
H	1.04462	5.57541	-0.33173
H	-1.63363	5.48557	-0.40793
C	-3.48437	-3.82764	0.01114
C	-4.45165	-4.13237	-0.96908
C	-3.59489	-4.37092	1.30608
C	-5.49252	-5.00114	-0.64672

C	-4.35993	-3.54977	-2.35787
C	-4.66461	-5.22412	1.58998
C	-2.61411	-4.02575	2.40196
C	-5.61603	-5.5602	0.62769
H	-6.22573	-5.24743	-1.40848
H	-4.7585	-5.62935	2.59259
C	-6.738	-6.51603	0.94403
C	3.61176	-3.58395	0.10196
C	3.69266	-4.50593	1.15847
C	4.67447	-3.53382	-0.80342
C	4.79166	-5.3374	1.30338
H	2.88954	-4.55635	1.88423
C	5.78531	-4.3674	-0.67478
H	4.62866	-2.84233	-1.63628
C	5.84731	-5.2748	0.38535
H	4.85579	-6.03965	2.12568
H	6.58123	-4.30308	-1.40345
C	3.33973	3.79063	-0.10001
C	3.382	4.74014	0.92384
C	4.38955	3.78389	-1.03229
C	4.42866	5.65589	1.02953
H	2.5931	4.75815	1.66662
C	5.43457	4.69016	-0.94438
H	4.37346	3.06507	-1.84285
C	5.46235	5.63334	0.08979
H	4.42809	6.36722	1.84354
H	6.23818	4.68758	-1.67081
C	-3.75626	3.55664	-0.03087
C	-3.87854	4.10259	1.26122
C	-4.76143	3.78078	-0.99336
C	-5.0024	4.87692	1.56167
C	-2.84737	3.84374	2.33431
C	-5.85739	4.57279	-0.65523

C	-4.65446	3.19479	-2.37966
C	-5.99623	5.13199	0.61748
H	-5.10477	5.28389	2.56276
H	-6.62263	4.75675	-1.4029
C	-7.17972	6.00418	0.95115
H	-7.6637	-6.22164	0.44403
H	-6.49222	-7.52799	0.60486
H	-6.92713	-6.56541	2.01811
H	-3.04103	-4.255	3.37969
H	-1.68454	-4.59243	2.30104
H	-2.343	-2.96825	2.38249
H	-4.5815	-2.47857	-2.35458
H	-3.35584	-3.66317	-2.77331
H	-5.06921	-4.0372	-3.02831
H	-5.42129	3.61043	-3.03478
H	-3.6762	3.3966	-2.8233
H	-4.77817	2.10821	-2.36243
H	-2.50798	2.80583	2.32679
H	-1.96002	4.46723	2.19487
H	-3.26024	4.06438	3.31995
H	-7.36322	6.0304	2.02706
H	-7.00777	7.03402	0.62039
H	-8.0865	5.65038	0.45521
H	-1.45521	1.03377	-2.20711
O	6.52982	6.48165	0.09739
C	6.60423	7.46285	1.12957
H	7.52002	8.0202	0.94445
H	5.74826	8.14328	1.08998
H	6.65529	6.99426	2.11694
O	6.88365	-6.13257	0.60696
C	7.98828	-6.10207	-0.2946
H	7.67521	-6.35755	-1.31135
H	8.4703	-5.11982	-0.29488

H	8.68917	-6.85072	0.0685

Table 3. Coordinates for compound 12.

C	-3.15087	-2.82909	-0.97767
C	-3.90427	-1.69644	-1.08095
C	-3.17176	-0.62853	-0.42098
C	-3.49237	0.76073	-0.48186
C	-2.52521	1.77977	-0.38994
C	-2.79611	3.17945	-0.31646
C	-1.67472	3.96846	-0.30152
C	-0.44204	3.24689	-0.36577
C	0.84024	3.8194	-0.34195
C	2.0441	3.04882	-0.25824
C	3.41995	3.56624	-0.30412
C	4.23624	2.4841	-0.23115
C	3.36232	1.3166	-0.11943
C	3.75684	-0.02152	-0.00432
C	-0.78049	-3.23853	-0.13528
C	-1.94344	-2.47169	-0.25742
N	-2.03498	-1.14037	0.10943
S	-0.77635	1.51455	-0.40154
N	2.04399	1.71216	-0.16402
N	0.503	-1.46319	0.96593
C	0.47223	-2.64252	0.25768
C	1.71036	-3.16007	-0.19797
C	1.58281	-0.70364	0.97428
C	2.76423	-1.03213	0.26054
C	2.84272	-2.36593	-0.2035
H	3.76273	-2.73085	-0.6478
H	1.73044	-4.14722	-0.64275
H	-3.35366	-3.80032	-1.40557
H	-4.83092	-1.56877	-1.62145
H	-3.80383	3.56621	-0.2443

H	-1.70097	5.04686	-0.2189
H	3.7058	4.60436	-0.39229
H	5.31726	2.4638	-0.2213
C	-0.7878	-4.65777	-0.61984
C	-0.93311	-5.69639	0.32774
C	-0.65478	-4.96053	-1.9927
C	-0.9504	-7.02067	-0.11961
C	-1.08146	-5.3964	1.80234
C	-0.67239	-6.303	-2.39101
C	-0.49566	-3.87729	-3.03755
C	-0.81873	-7.34715	-1.47374
H	-1.07098	-7.81762	0.61067
H	-0.56807	-6.53526	-3.44836
C	-0.80898	-8.78868	-1.92468
C	-4.91445	1.11177	-0.73348
C	-5.29597	1.9617	-1.78652
C	-5.92418	0.56711	0.08115
C	-6.6387	2.2578	-2.01087
H	-4.53733	2.37505	-2.4442
C	-7.2632	0.8741	-0.14693
H	-5.64414	-0.06957	0.91459
C	-7.64698	1.72084	-1.19741
H	-6.90921	2.91199	-2.83576
H	-8.0235	0.45517	0.50726
C	0.95781	5.30576	-0.38915
C	0.46601	6.03622	-1.48347
C	1.57666	6.01218	0.65629
C	0.59119	7.42397	-1.52848
H	0.00177	5.51002	-2.31229
C	1.69161	7.39981	0.60681
H	1.955	5.47066	1.51803
C	1.20086	8.13236	-0.48399
H	0.21345	7.9644	-2.39269

H	2.16761	7.92335	1.43197
C	5.17315	-0.42109	-0.25475
C	5.71662	-0.35275	-1.55851
C	5.9668	-0.88829	0.82084
C	7.0436	-0.75109	-1.758
C	4.90112	0.11266	-2.74611
C	7.29089	-1.25879	0.57265
C	5.42284	-0.96766	2.23016
C	7.85063	-1.1984	-0.70855
H	7.45431	-0.71314	-2.76433
H	7.90149	-1.60423	1.4038
C	9.29114	-1.58586	-0.94303
H	0.18207	-9.23811	-1.78539
H	-1.51906	-9.39127	-1.34994
H	-1.06317	-8.87828	-2.98454
H	-0.23432	-4.31197	-4.0055
H	-1.41931	-3.30313	-3.17008
H	0.28661	-3.16053	-2.76775
H	-0.2046	-4.87308	2.19968
H	-1.94631	-4.75376	1.9993
H	-1.20833	-6.31814	2.37519
H	6.2221	-1.19579	2.93938
H	4.956	-0.02634	2.53776
H	4.65997	-1.74773	2.32923
H	3.89732	-0.32279	-2.75056
H	4.77509	1.20091	-2.74815
H	5.39498	-0.16433	-3.68102
H	9.46776	-1.85683	-1.9877
H	9.96539	-0.7548	-0.70135
H	9.58508	-2.43205	-0.31446
H	1.53886	0.18495	1.58422
Pd	-1.16622	-0.76292	1.95186
Cl	-3.19563	-0.06596	2.96577

Cl	0.03261	-0.53209	3.99207
C	1.30411	9.63813	-0.51827
H	0.44999	10.10136	-0.0087
H	2.20977	9.98836	-0.01435
H	1.31461	10.01523	-1.54486
C	-9.10272	2.02296	-1.45832
H	-9.22592	2.96963	-1.99186
H	-9.56248	1.23855	-2.07253
H	-9.67196	2.07753	-0.5256

Table 4. Coordinates for compound 13.

C	-3.84957	-2.15864	-0.83477
C	-4.2737	-0.873	-0.82759
C	-3.21648	-0.06902	-0.22614
C	-3.15644	1.37712	-0.23425
C	-1.94189	2.021	-0.29666
C	-1.74171	3.45978	-0.33078
C	-0.47477	3.94003	-0.43119
C	0.63434	3.00182	-0.47326
C	1.97135	3.29946	-0.44773
C	2.94127	2.2166	-0.32932
C	4.40627	2.31818	-0.2937
C	4.87528	1.05667	-0.18447
C	3.70622	0.17644	-0.14171
C	3.69761	-1.18489	-0.05289
C	-1.58515	-3.16523	-0.30561
C	-2.51298	-2.16573	-0.26001
N	-2.22118	-0.86503	0.16995
Te	-0.07899	1.05637	-0.35301
N	2.55537	0.96068	-0.24883
N	0.18408	-1.96689	0.90877
C	-0.16656	-2.908	0.0016
C	0.83354	-3.53154	-0.75313

C	1.43262	-1.50133	0.94991
C	2.42529	-1.9203	0.06159
C	2.12799	-3.03299	-0.73083
H	2.89022	-3.46355	-1.37239
H	0.56789	-4.34875	-1.41275
H	-4.34668	-3.02279	-1.25264
H	-5.1893	-0.47356	-1.23945
H	-2.58861	4.13313	-0.24808
H	-0.27821	5.00753	-0.43985
H	4.97652	3.23433	-0.35092
H	5.90316	0.72787	-0.11619
C	-1.94765	-4.5035	-0.84882
C	-2.11652	-5.56261	0.06054
C	-2.09259	-4.70839	-2.23019
C	-2.45196	-6.8199	-0.43424
C	-1.94422	-5.33564	1.54154
C	-2.42195	-5.98753	-2.68174
C	-1.89081	-3.58736	-3.22165
C	-2.61177	-7.05123	-1.80175
H	-2.5902	-7.64155	0.2645
H	-2.52876	-6.15538	-3.75079
C	-3.00078	-8.41671	-2.307
C	-4.45686	2.09221	-0.30281
C	-4.75238	2.98443	-1.33422
C	-5.4392	1.85144	0.6706
C	-5.98165	3.63649	-1.4013
H	-4.01641	3.16522	-2.11226
C	-6.6649	2.49104	0.61585
H	-5.22019	1.16674	1.48514
C	-6.94546	3.39029	-0.42086
H	-6.17474	4.31899	-2.22005
H	-7.42288	2.31407	1.37118
C	2.46432	4.70024	-0.5008

C	2.14667	5.53184	-1.58441
C	3.24758	5.22874	0.5266
C	2.59424	6.84117	-1.63525
H	1.54787	5.13811	-2.4004
C	3.706	6.54333	0.48989
H	3.49619	4.60908	1.38342
C	3.37842	7.35775	-0.59682
H	2.35279	7.48366	-2.47508
H	4.30478	6.91673	1.31162
C	4.95254	-1.97617	-0.15982
C	5.64008	-2.05123	-1.3836
C	5.41904	-2.67784	0.96846
C	6.79782	-2.82882	-1.45262
C	5.15282	-1.33112	-2.61902
C	6.58451	-3.42977	0.85603
C	4.68335	-2.60774	2.2834
C	7.28898	-3.51757	-0.34628
H	7.32687	-2.89853	-2.40008
H	6.95394	-3.96286	1.72897
C	8.55851	-4.32502	-0.43212
H	-2.54103	-9.20654	-1.70675
H	-4.08618	-8.55404	-2.2514
H	-2.70193	-8.55743	-3.3487
H	-1.7416	-3.98472	-4.2279
H	-2.75846	-2.9201	-3.2531
H	-1.02358	-2.97003	-2.96758
H	-0.92076	-5.0265	1.7815
H	-2.60739	-4.54352	1.90396
H	-2.1596	-6.24559	2.10515
H	5.27887	-3.04554	3.08712
H	4.44981	-1.57421	2.55727
H	3.7339	-3.15225	2.23904
H	4.06695	-1.40054	-2.73141

H	5.40037	-0.26522	-2.58219
H	5.61615	-1.75048	-3.51482
H	8.82209	-4.54553	-1.46944
H	9.39553	-3.77809	0.01528
H	8.46262	-5.27157	0.10721
H	1.63935	-0.74955	1.70107
Pd	-1.25202	-0.98865	2.01473
Cl	-2.97617	0.10724	3.12047
Cl	-0.08185	-1.39123	3.98027
O	3.76854	8.64675	-0.73644
O	-8.1719	3.9633	-0.389
C	-8.50364	4.88586	-1.40898
C	4.56143	9.21782	0.28745
H	-8.48704	4.41366	-2.39828
H	-9.51551	5.22624	-1.19114
H	-7.82462	5.7465	-1.40718
H	5.5126	8.68481	0.4016
H	4.75943	10.24394	-0.02081
H	4.03254	9.22485	1.24749

Table 5. Coordinates for compound 14.

C	-3.14773	-2.39043	-1.43548
C	-3.88119	-1.24662	-1.39179
C	-3.10175	-0.2745	-0.63189
C	-3.30556	1.13173	-0.70493
C	-2.2489	2.04989	-0.69318
C	-2.38908	3.46768	-0.64918
C	-1.20687	4.14573	-0.7425
C	-0.05584	3.30303	-0.81655
C	1.26104	3.766	-0.76057
C	2.39699	2.93081	-0.60161
C	3.77654	3.39882	-0.38553
C	4.53076	2.28842	-0.19399
C	3.60995	1.15316	-0.25378
C	3.91214	-0.18191	-0.02907
C	-0.78394	-2.92496	-0.72094
C	-1.93339	-2.14424	-0.67569

N	-2.00672	-0.87275	-0.10871
S	-0.54444	1.61523	-0.84648
N	2.33744	1.59402	-0.54297
N	0.51016	-1.42948	0.72103
C	0.45868	-2.50879	-0.13934
C	1.67109	-3.08342	-0.58506
C	1.63734	-0.73926	0.78108
C	2.82963	-1.11723	0.12055
C	2.86285	-2.41109	-0.42825
H	3.78428	-2.81011	-0.83426
H	1.63513	-4.00151	-1.15347
H	-3.36369	-3.29661	-1.97886
H	-4.80716	-1.03526	-1.90311
H	-3.34985	3.94442	-0.52395
H	-1.12301	5.22172	-0.71283
H	4.09661	4.42921	-0.36713
H	5.58611	2.22671	0.02532
C	-0.73554	-3.99838	-1.77278
C	-0.62723	-3.57636	-3.11914
C	-0.7317	-5.36978	-1.47495
C	-0.57891	-4.53402	-4.13039
C	-0.52243	-2.11471	-3.48654
C	-0.68444	-6.2971	-2.52168
C	-0.76924	-5.88705	-0.06013
C	-0.6228	-5.90274	-3.85505
H	-0.48898	-4.20247	-5.16019
H	-0.68432	-7.3554	-2.27985
C	-0.60025	-6.91607	-4.97087
C	-4.67929	1.60783	-0.99894
C	-4.93675	2.47318	-2.07345
C	-5.76898	1.16392	-0.23919
C	-6.23127	2.8861	-2.36291
H	-4.11561	2.80514	-2.69675
C	-7.06281	1.58535	-0.52922
H	-5.59658	0.49821	0.59481
C	-7.31917	2.45242	-1.59558
H	-6.40228	3.55025	-3.20368
H	-7.88589	1.23857	0.0866
C	1.49758	5.24094	-0.77189
C	2.04087	5.86377	-1.8985
C	1.21581	6.02262	0.35278
C	2.28768	7.23471	-1.90222
H	2.27278	5.26969	-2.77522

C	1.46649	7.39184	0.3448
H	0.80986	5.54926	1.23926
C	2.00229	8.02285	-0.78311
H	2.71013	7.69779	-2.7879
H	1.24855	7.97793	1.23154
C	5.31285	-0.66995	-0.03717
C	6.08017	-0.6157	-1.21733
C	5.86353	-1.21938	1.13973
C	7.3892	-1.10346	-1.19446
C	5.51206	-0.07877	-2.50976
C	7.1787	-1.68094	1.12068
C	5.06262	-1.29052	2.41722
C	7.9598	-1.6307	-0.03636
H	7.97383	-1.07652	-2.10868
H	7.60515	-2.08842	2.03211
C	9.38774	-2.11395	-0.02397
H	-1.58549	-6.99724	-5.44184
H	0.1095	-6.6309	-5.75132
H	-0.32676	-7.90662	-4.60259
H	-0.2836	-6.86248	0.00362
H	-0.26304	-5.21185	0.62886
H	-1.79818	-6.01098	0.28827
H	-1.48248	-1.60247	-3.3829
H	0.18806	-1.58737	-2.84616
H	-0.19487	-2.00761	-4.52157
H	5.70687	-1.54114	3.26123
H	4.56708	-0.34063	2.63145
H	4.27879	-2.05086	2.35499
H	4.48902	-0.42401	-2.67368
H	5.4808	1.01415	-2.51021
H	6.12396	-0.39649	-3.3556
H	9.72847	-2.37237	-1.02851
H	10.05661	-1.33735	0.36211
H	9.50477	-2.99142	0.61626
H	1.60996	0.14804	1.39722
C	2.24188	9.51134	-0.79851
C	-8.72338	2.88333	-1.93132
Ru	-1.18957	-0.8447	1.91994
C	-1.0142	-1.2555	4.05222
C	-2.26168	-0.55088	3.83494
C	-3.06571	-1.33203	2.96508
C	-1.08193	-2.48792	3.31998
C	-2.34933	-2.54962	2.64407

C	-2.94792	-3.73592	1.96402
H	-3.522	-4.33704	2.67998
H	-3.6236	-3.4417	1.15975
H	-2.18267	-4.37449	1.53403
C	-4.46519	-1.03066	2.53686
H	-4.65346	-1.38565	1.52244
H	-5.18492	-1.52653	3.19869
H	-4.66592	0.04035	2.56825
C	-0.03491	-3.5557	3.32202
H	-0.16312	-4.21924	4.18442
H	-0.09188	-4.16595	2.42179
H	0.96765	-3.12839	3.37459
C	-2.65328	0.73251	4.49109
H	-3.4152	1.25874	3.91457
H	-3.05929	0.53428	5.49017
H	-1.7955	1.39655	4.58832
C	0.08799	-0.81221	4.96007
H	1.03299	-1.29198	4.69834
H	0.23202	0.26631	4.88814
H	-0.13705	-1.06294	6.00394
Cl	-0.23989	1.49133	2.31144
H	-8.75035	3.92293	-2.26573
H	-9.13458	2.27004	-2.7403
H	-9.38655	2.78104	-1.07034
H	3.04032	9.77792	-1.49406
H	1.33979	10.0475	-1.11215
H	2.51243	9.88003	0.19332

Table 6. Coordinates for compound 15.

C	-3.60221	-1.96901	-1.32649
C	-4.09616	-0.70203	-1.23741
C	-3.10227	0.09189	-0.53009
C	-3.0072	1.52214	-0.60334
C	-1.76311	2.14454	-0.68412
C	-1.52126	3.54999	-0.62066
C	-0.22663	3.99584	-0.72187
C	0.82827	3.0343	-0.8214
C	2.18814	3.30676	-0.6682
C	3.12964	2.24951	-0.50595
C	4.54312	2.37367	-0.13105
C	5.01508	1.11049	0.02594

C	3.88257	0.2115	-0.20005
C	3.86801	-1.16072	-0.0267
C	-1.32841	-2.93779	-0.78128
C	-2.31972	-1.9729	-0.64704
N	-2.1171	-0.71483	-0.07208
Te	0.05453	1.11345	-0.96699
N	2.77812	0.95592	-0.57149
N	0.27685	-1.81489	0.70187
C	0.00014	-2.77476	-0.2507
C	1.07194	-3.48897	-0.82932
C	1.52032	-1.35605	0.78152
C	2.60076	-1.86306	0.03011
C	2.37226	-3.05906	-0.66813
H	3.18923	-3.56562	-1.16728
H	0.85169	-4.31414	-1.49093
H	-4.02402	-2.80703	-1.85857
H	-4.99112	-0.31009	-1.69506
H	-2.33336	4.24481	-0.44487
H	0.00444	5.05185	-0.65732
H	5.07475	3.30151	0.0141
H	6.00036	0.80099	0.34071
C	-1.53236	-3.96938	-1.85442
C	-1.48485	-3.52958	-3.19931
C	-1.70189	-5.33628	-1.58062
C	-1.68365	-4.45225	-4.22437
C	-1.16809	-2.09629	-3.55645
C	-1.90265	-6.22696	-2.64066
C	-1.66219	-5.89358	-0.1808
C	-1.91562	-5.80546	-3.96694
H	-1.63815	-4.10818	-5.25288
H	-2.03863	-7.28049	-2.41631
C	-2.16557	-6.77513	-5.09344
C	-4.26336	2.28522	-0.76918
C	-4.40285	3.25752	-1.77688
C	-5.37099	2.03288	0.04674
C	-5.58738	3.95279	-1.94492
H	-3.57628	3.44651	-2.45007
C	-6.56819	2.72884	-0.1044
H	-5.2935	1.28979	0.82792
C	-6.67952	3.69893	-1.10417
H	-5.69419	4.69167	-2.72973
H	-7.3935	2.51147	0.5589
C	2.68016	4.70534	-0.54106

C	3.55319	5.24002	-1.48951
C	2.3057	5.51489	0.5424
C	4.03757	6.54444	-1.3832
H	3.85739	4.63238	-2.33406
C	2.78113	6.81129	0.66442
H	1.64909	5.11165	1.3039
C	3.64825	7.33702	-0.30104
H	4.70599	6.92389	-2.14333
H	2.49821	7.43194	1.50596
C	5.12692	-1.94252	0.0256
C	5.98759	-1.98397	-1.09005
C	5.44542	-2.67595	1.18942
C	7.1539	-2.74984	-1.01593
C	5.66616	-1.25506	-2.37362
C	6.62669	-3.41442	1.22369
C	4.54697	-2.64923	2.40221
C	7.49645	-3.46405	0.13143
H	7.80639	-2.79423	-1.88227
H	6.87675	-3.96279	2.12671
C	8.77966	-4.25178	0.20332
H	-3.21174	-6.73438	-5.41474
H	-1.54916	-6.53969	-5.96419
H	-1.95383	-7.80185	-4.78871
H	-1.21366	-6.88903	-0.18049
H	-1.08096	-5.26218	0.48873
H	-2.66832	-5.99059	0.23648
H	-2.01526	-1.43163	-3.37092
H	-0.33052	-1.71233	-2.97016
H	-0.90718	-2.01896	-4.61285
H	5.05931	-3.07048	3.2684
H	4.2362	-1.63096	2.64723
H	3.63475	-3.22982	2.23696
H	4.60642	-1.32979	-2.62548
H	5.8983	-0.18925	-2.29833
H	6.24863	-1.66631	-3.19978
H	9.15452	-4.49244	-0.79339
H	9.55759	-3.67958	0.71997
H	8.64275	-5.18499	0.75476
H	1.67345	-0.53825	1.47303
O	4.05974	8.622	-0.0992
C	4.94752	9.20232	-1.05195
H	5.14095	10.21378	-0.70089
H	5.88924	8.6475	-1.1038

H	4.4902	9.24181	-2.04524
O	-7.79898	4.43513	-1.34137
C	-8.94307	4.21136	-0.51813
H	-9.30117	3.18203	-0.61296
H	-8.72204	4.42914	0.53083
H	-9.70663	4.89699	-0.87889
Ru	-1.27476	-0.97646	1.96019
C	-1.15964	-1.47404	4.06879
C	-2.27648	-0.56718	3.8887
C	-3.2031	-1.18047	3.00654
C	-1.43524	-2.65658	3.30229
C	-2.70118	-2.48896	2.64075
C	-3.49584	-3.54104	1.94025
H	-4.1162	-4.0924	2.65773
H	-4.15907	-3.10904	1.18972
H	-2.85444	-4.25782	1.4369
C	-4.53613	-0.6382	2.60491
H	-4.78116	-0.90404	1.5756
H	-5.32396	-1.04707	3.24817
H	-4.56245	0.44758	2.69575
C	-0.57681	-3.88057	3.26292
H	-0.81287	-4.54343	4.10278
H	-0.72932	-4.443	2.34286
H	0.4819	-3.62408	3.32634
C	-2.44497	0.74367	4.58471
H	-3.11024	1.40729	4.03081
H	-2.87307	0.58777	5.58202
H	-1.48766	1.25159	4.693
C	0.00515	-1.24949	4.97894
H	0.86052	-1.86043	4.68447
H	0.31842	-0.20549	4.95248
H	-0.25071	-1.50796	6.01373
Cl	0.05995	1.14489	2.38139

Table S7. Computational details for the optimized structures of compounds.

Structure	Zero-point vibration Energy [a.u.]	Lowest freq. [cm^{-1}]
**5**	-2608.422123	12.17
**6**	-2368.624309	11.70
**12**	-3655.631894	12.88
**13**	-3415.874308	11.86

**15**	-3552.419813	11.53
**16**	-3312.664106	11.18

EPR

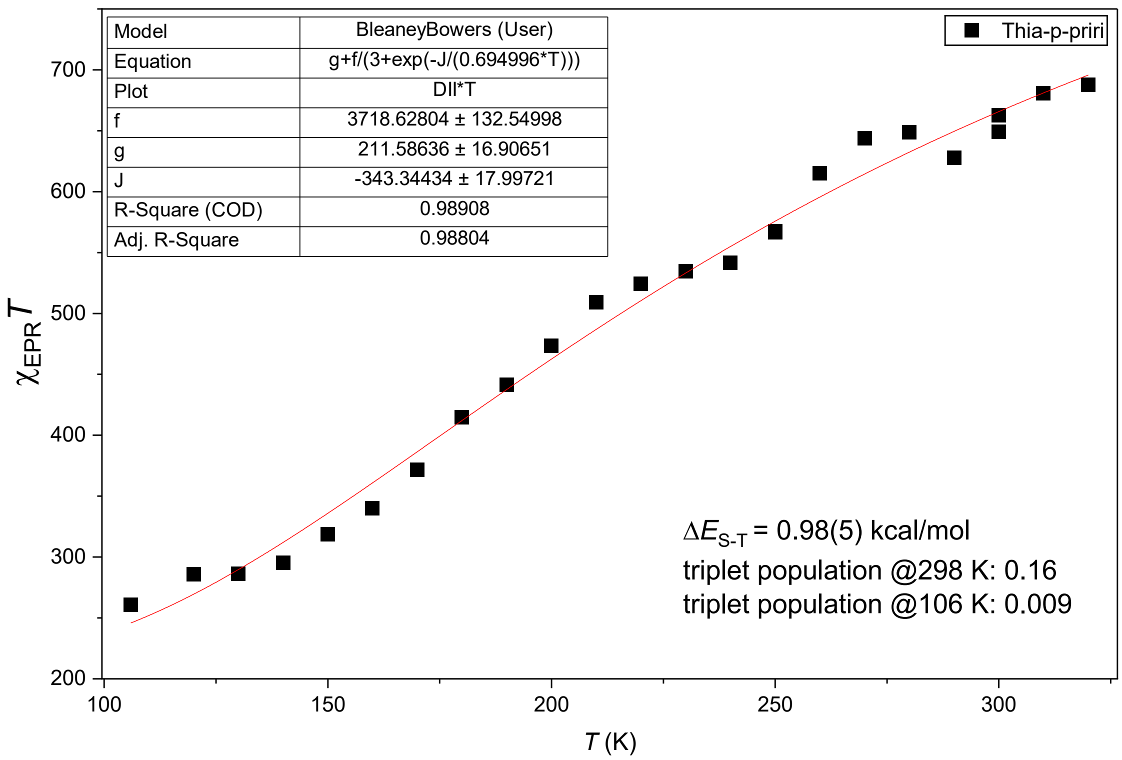

Figure S64. The associated χ_{EPR}T vs T plot (B) for 5.

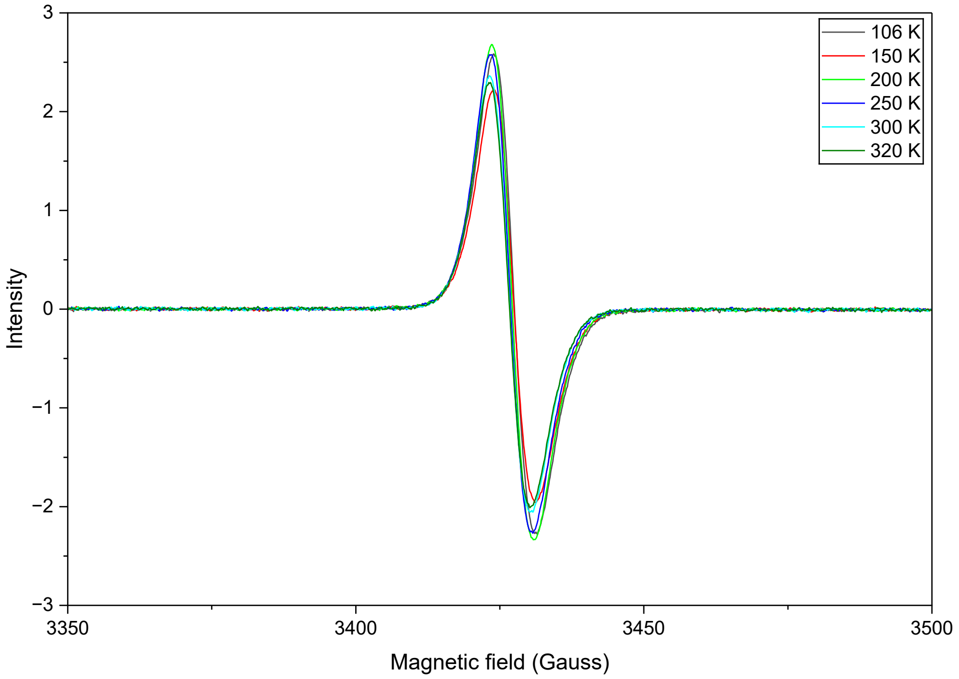

Figure *S*65. X-band EPR spectra of 5 in the solid state recorded at variable temperatures*.*

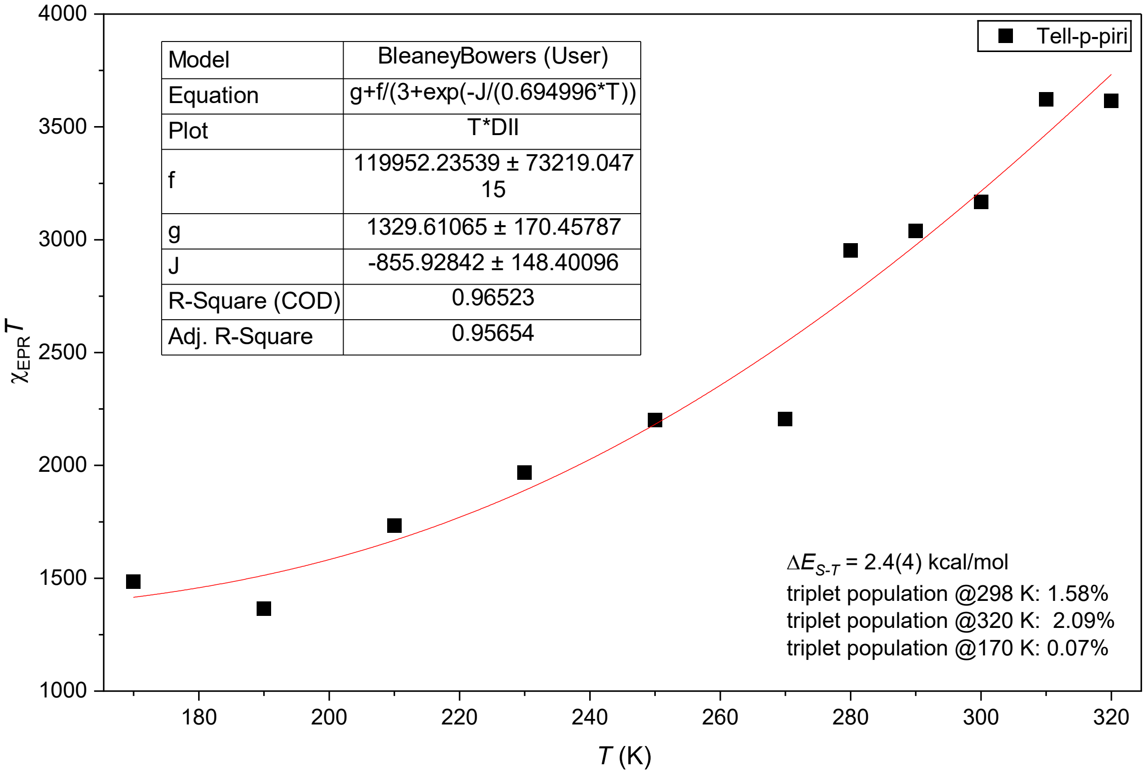

Figure S66. The associated χ_{EPR}T vs T plot (B) for 6.

Catalytic reactions

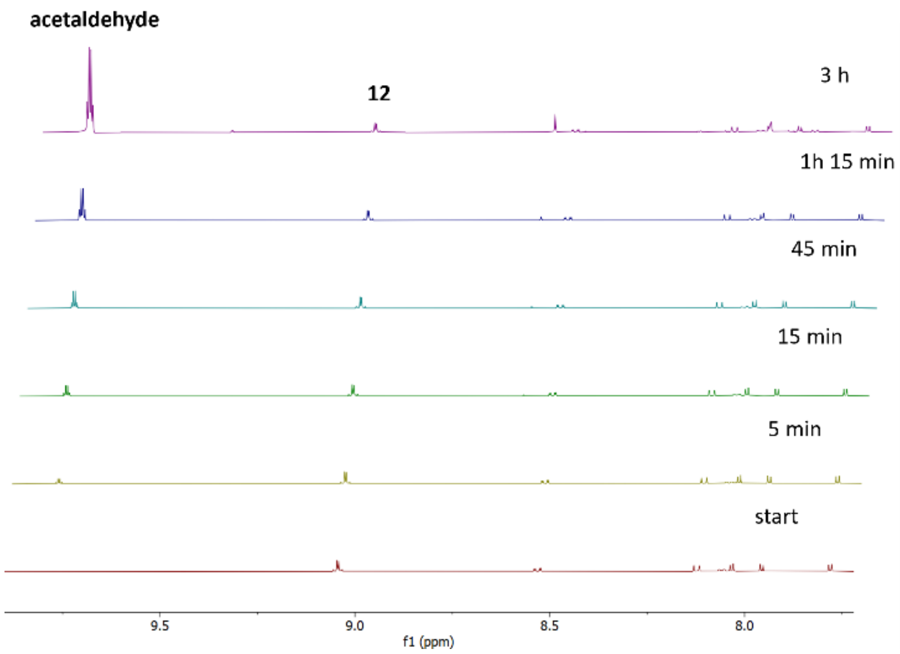

Figure S67. TEA dealkylation following in time by ^{1}H NMR spectroscopy ($CDCl_3$, 300 K, 600 Mz) with formyl signal marked.

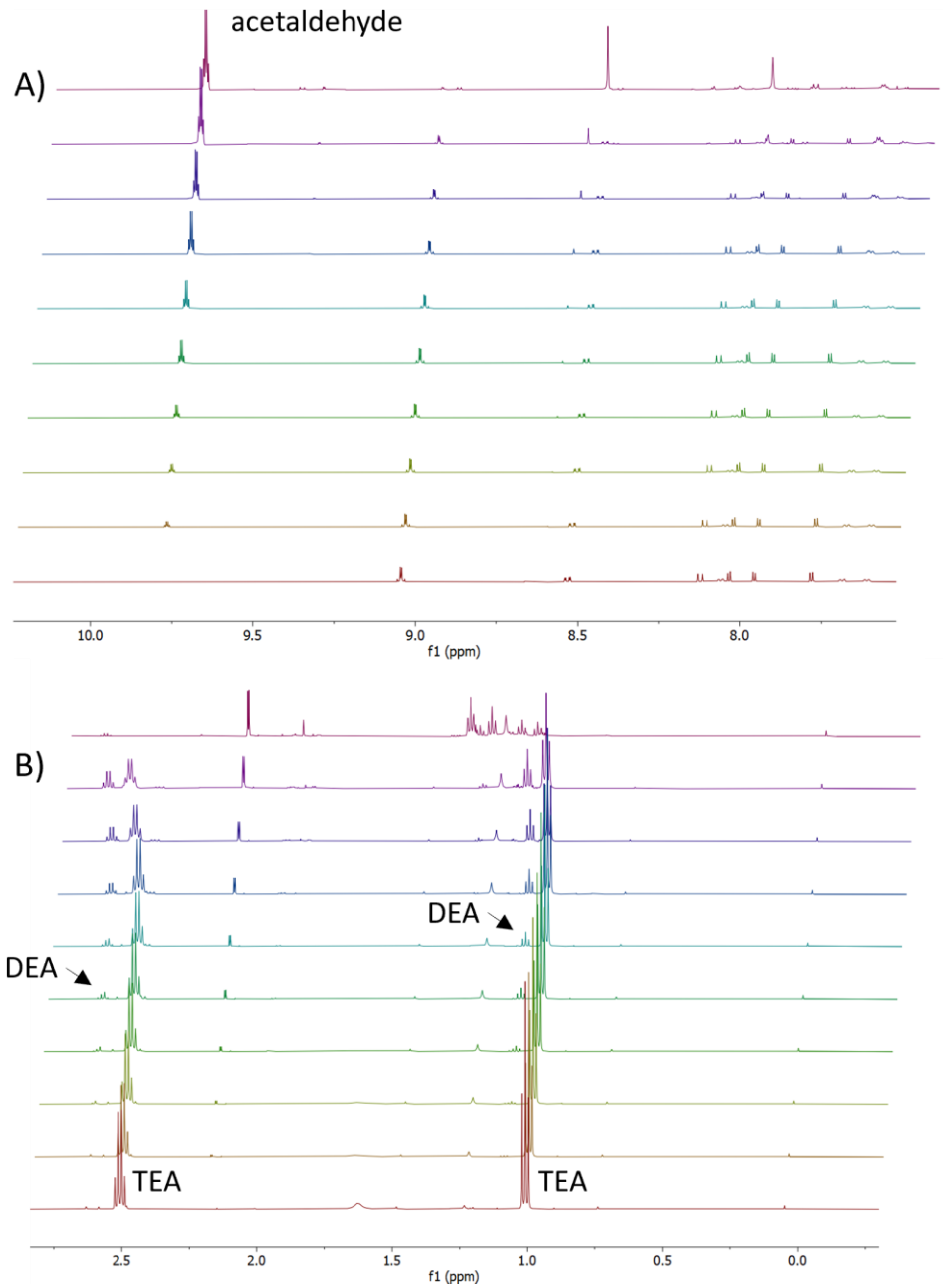

Figure S68. TEA dealkylation following in time by ^{1}H NMR spectroscopy ($CDCl_3$, 300 K, 600 Mz). The scale of the spectra in Figure B is one-third that of Figure A. General conditions: 2 mg of catalyst 12 or 13, 0.1 mmol of tertiary amine, 1 ml of chloroform. The mixture was stirred for 3 hours at room temperature in open air under a blue light (470 nm).

The reaction was more efficient when light was used as a co-catalyst. For both catalysts, the amines conversion efficiency was similar, with a few percent advantage for **12**. The experiment with blue light (470 nm) yielded a higher substrate conversion rate than the experiments with white light or without a catalyst (**12**/**13**). The reaction is conducted under aerobic conditions in chloroform stabilized with amylene as the solvent. This reaction was followed by the NMR (Figure 6). The proton spectra below show an increase in the acetaldehyde signal intensity over the course of the experiment, indicating a successful catalytic reaction.

Titrations

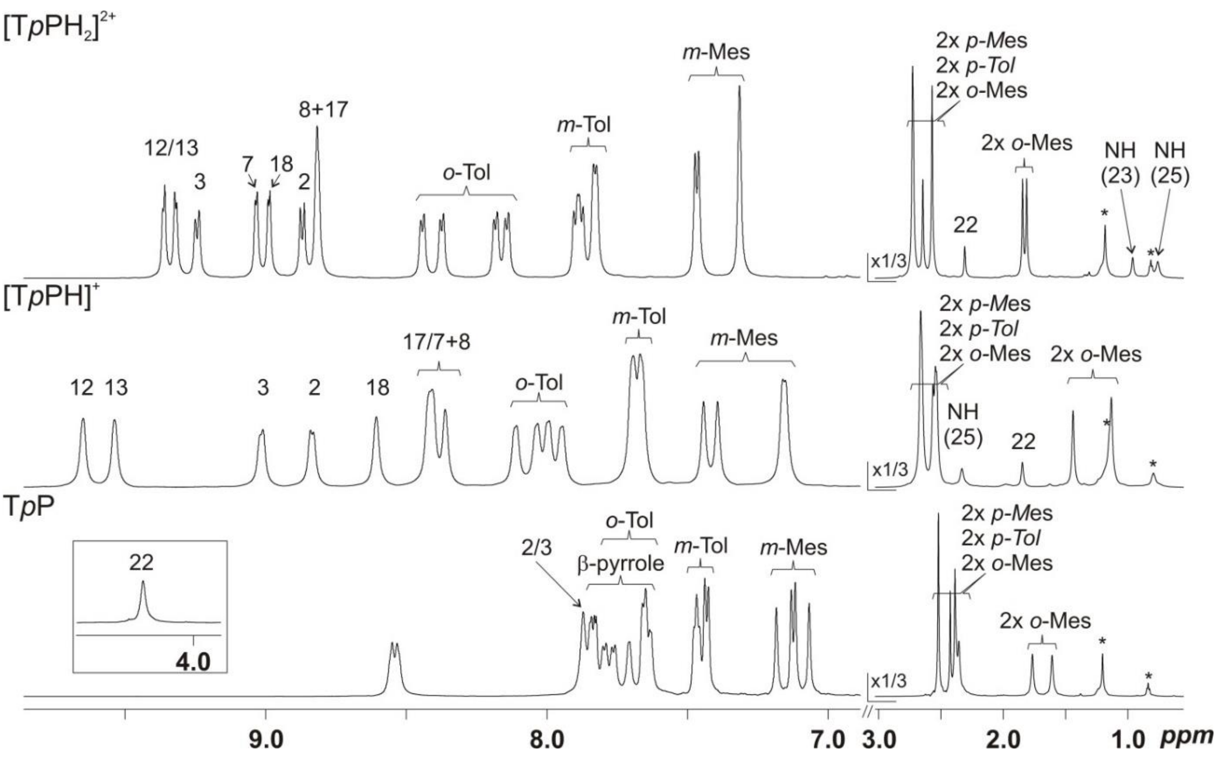

Figure S69. Titration of 5 with HBF_4 (600 MHz, CD_2Cl_2, 200 K).

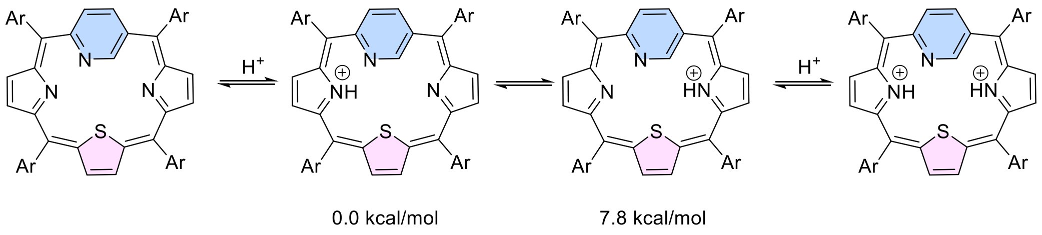

A)

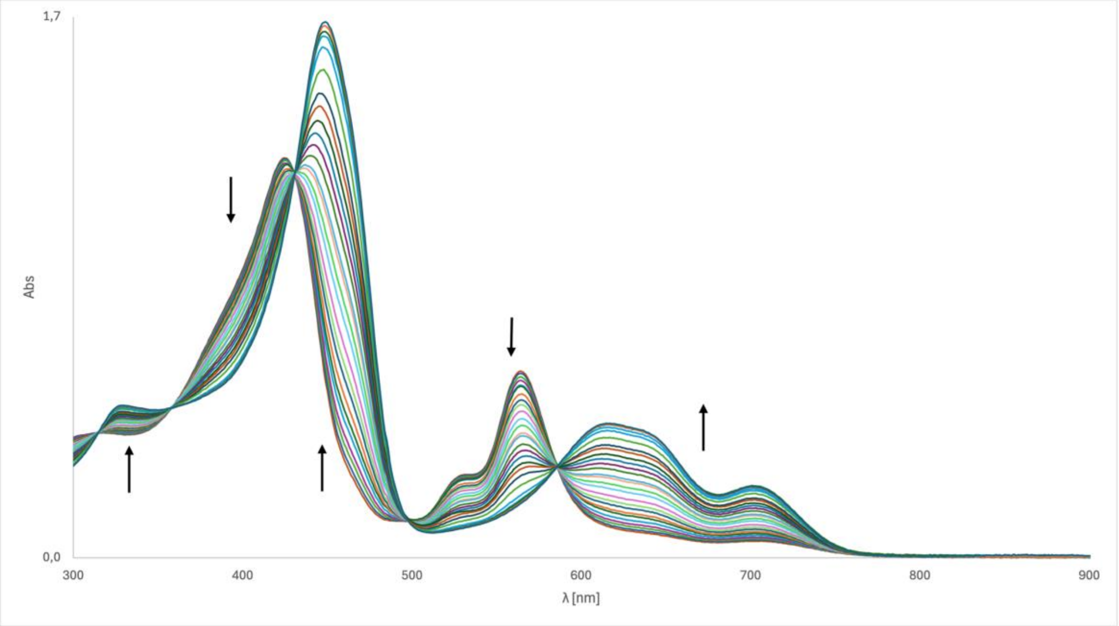

B)

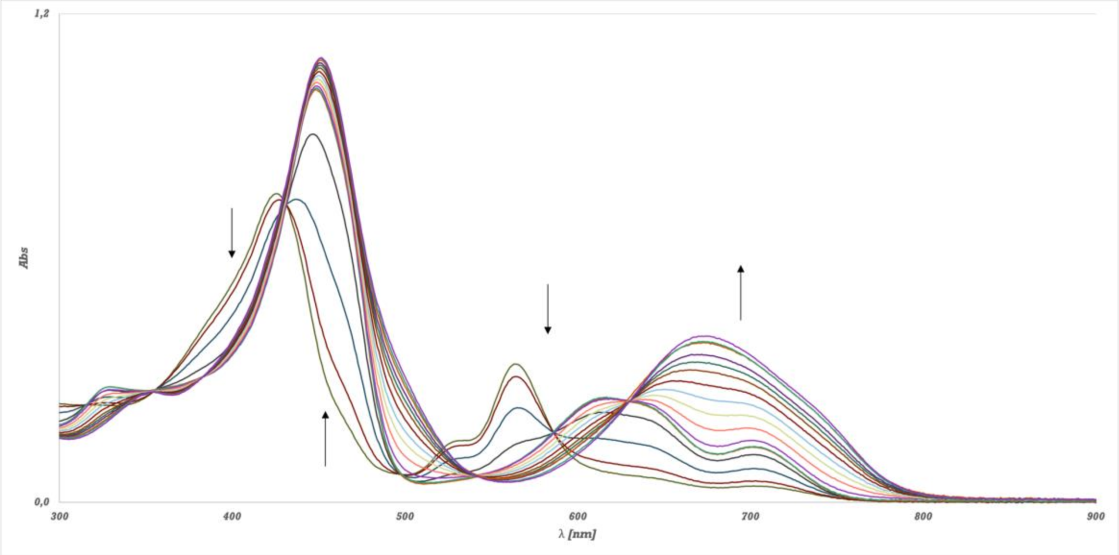

Figure S70. Titration of 5 A) with TFA to monocationic form, B) with HBF_4 to dicationic form (RT, DCM).

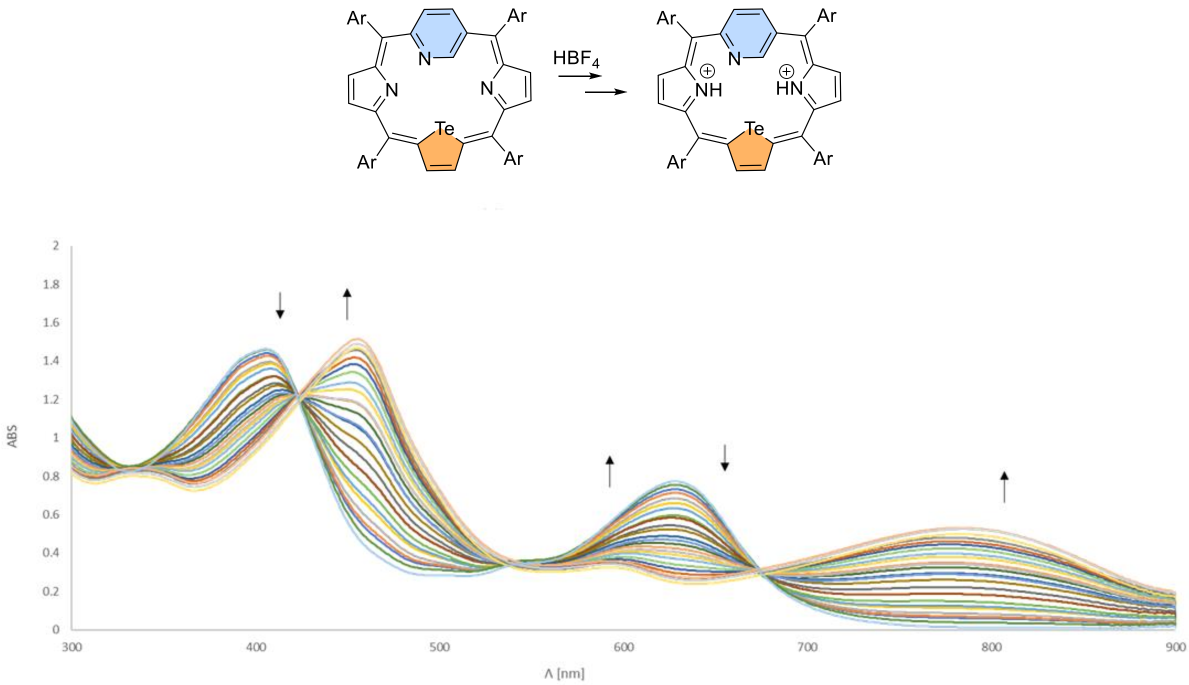

Figure S71. Titration of 6 with HBF$_4$ (RT, DCM).

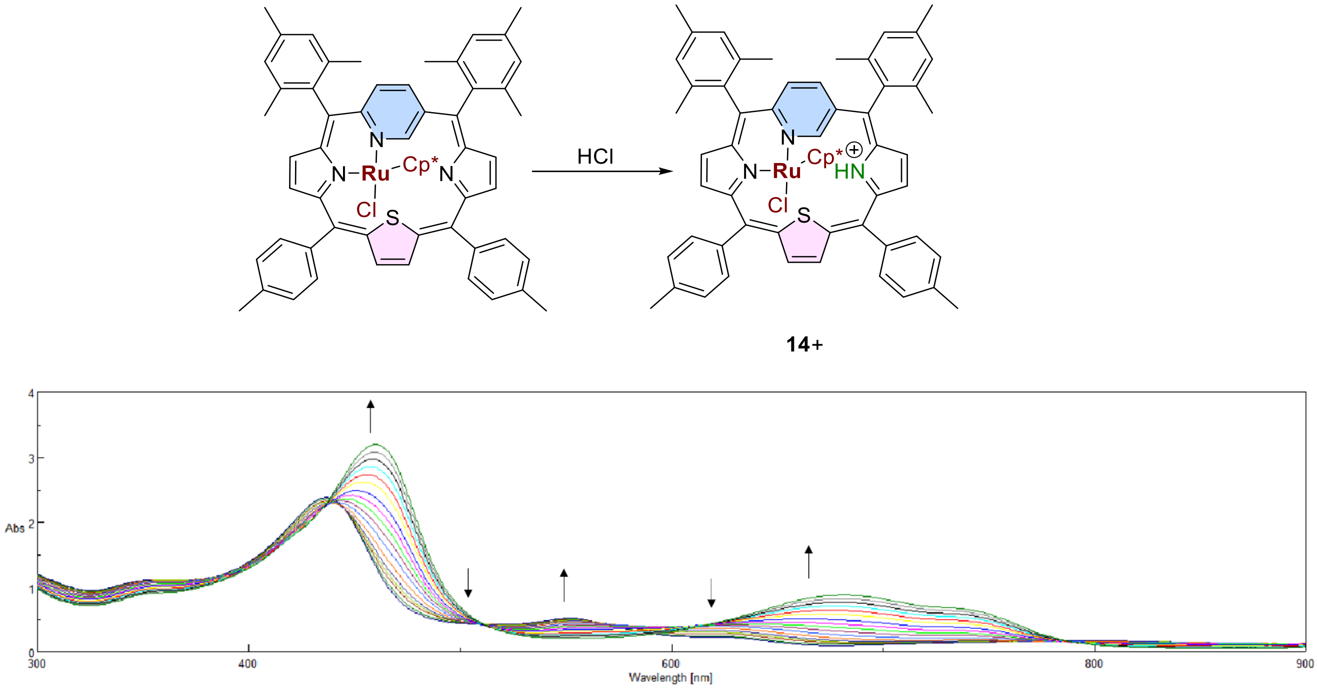

Figure S72. Titration of 14 with HCl (RT, DCM).

CD spectroscopy

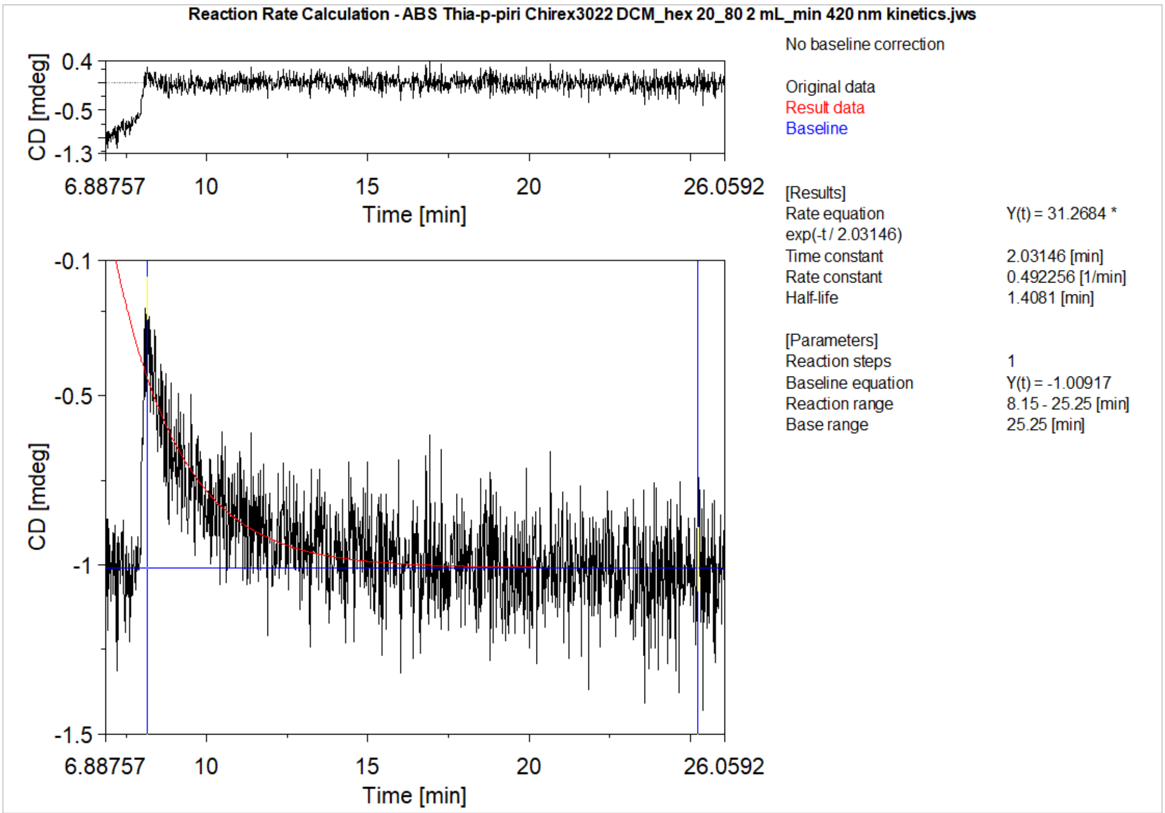

Figure S73. Changes in the CD intensity.

The racemization process - that is, inversion of the pyridine moiety through the interior of the macrocycle - is very slow on the ^{1}H NMR timescale. This is evidenced by the persistent observation of four *meta*-H and four *ortho*-CH_3 resonances throughout the entire temperature range studied. If racemization were fast, an averaged pattern would be expected, giving rise to only two *meta*-H and two *ortho*-CH_3 resonances. Importantly, CD experiments following HPLC separation of the principal enantiomers revealed that racemization occurs, with an enantiomeric half-life of approximately 60 s (see Figure S71).

Photoluminescence

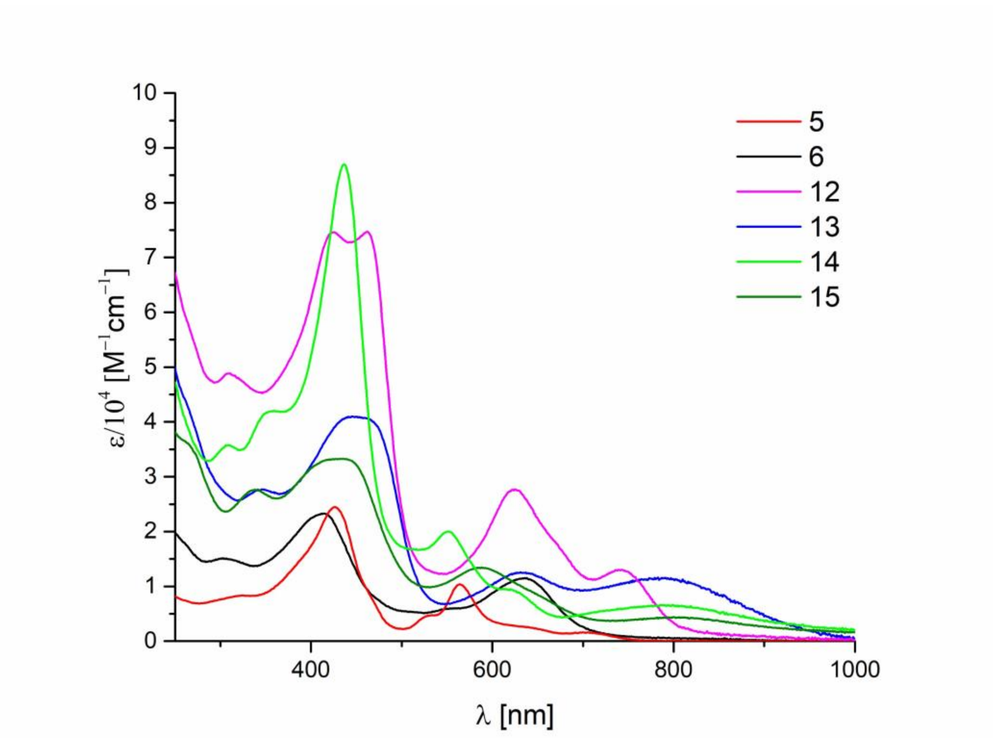

Figure S74. UV-Vis spectra of compounds 5, 6, 12, 13, 14, and 15.

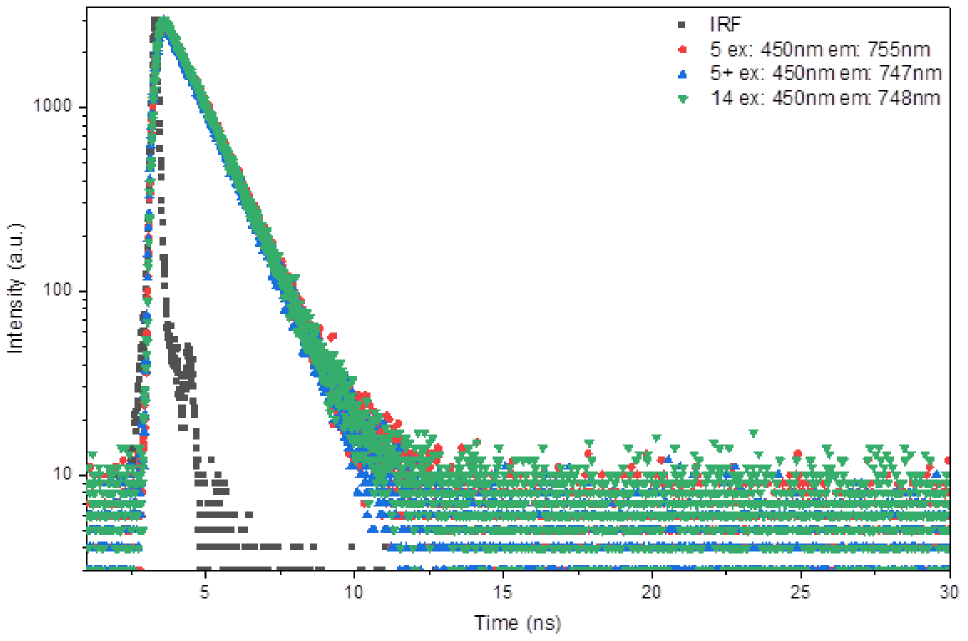

Figure S75. Fluorescence decay curves of samples 5, 5+, and 14 in dichloromethane, along with the instrument response function (IRF).

Table S8. Fluorescence lifetime (τ), the correlation coefficient (χ^2), and fluorescence quantum yield (QY, %) for samples 5, 5+, and 14 in dichloromethane.

Sample	τ (ns)	χ^2	QY (%)
**5**	1.22	1.2588	7.3%
**5+**	1.21	1.1635	5.0%
**14**	1.21	1.2702	4.9%

The fluorescence decay curves of samples **5**, **5+**, and **14** in dichloromethane are presented in Figure S65. All decay profiles were satisfactorily fitted using a monoexponential model (Eq. 1):

$$I(t) = A_0 + A \times \exp\left(-\frac{t}{\tau}\right) \quad (1)$$

where $I(t)$denotes the fluorescence intensity at time t, A_0 is the background intensity, A is the amplitude of the decay component, and τ represents the fluorescence lifetime of the emitting species. A single lifetime component adequately described the decay curves of all samples. The fitted parameters are summarized in Table S8.

REFERENCES:

[1] G. M. Sheldrick, *Acta Crystallogr. A* **2015** 71, 3–8, 11

[2] G. M. Sheldrick, *Acta Crystallogr. C* **2015** 71, 3–8.

[3] M. J. Frisch, G. W. Trucks, H. B. Schlegel, G. E. Scuseria, M. A. Robb, J. R. Cheeseman, G. Scalmani, V. Barone, G. A. Petersson, H. Nakatsuji, X. Li, M. Caricato, A. V. Marenich, J. Bloino, B. G. Janesko, R. Gomperts, B. Mennucci, H. P. Hratchian, J. V. Ortiz, A. F. Izmaylov, J. L. Sonnenberg F. Ding, F. Lipparini, F. Egidi, J. Goings, B. Peng, A. Petrone, T. Henderson, D. Ranasinghe, V. G. Zakrzewski, J. Gao, N. Rega, G. Zheng, W. Liang, M. Hada, M. Ehara, K. Toyota, R. Fukuda, J. Hasegawa, M. Ishida, T. Nakajima, Y. Honda, O. Kitao, H. Nakai, T. Vreven, K. Throssell, Jr., J. A. Montgomery J. E. Peralta, F. Ogliaro, M. J. Bearpark, J. J. Heyd, E. N. Brothers, K. N. Kudin, V. N. Staroverov, T. A. Keith, R. Kobayashi, J. Normand, K. Raghavachari, A. P. Rendell, J. C. Burant, S. S. Iyengar, J. Tomasi, M. Cossi, J. M. Millam, M. Klene, C. Adamo, R. Cammi, J. W. Ochterski, R. L. Martin, K. Morokuma, O. Farkas, J. B. Foresman, D. J. Fox, Gaussian 16 Rev. C.01. Wallingford, CT, **2016**.

[4] C. Lee, W. Yang, R. G. Parr, Development of the Colle-Salvetti correlation-energy formula into a functional of the electron density. *Phys. Rev. B* **1988**, 37, 785-789.

[5] A. D. Becke, Density-functional exchange-energy approximation with correct asymptotic behavior. *Phys. Rev. A* **1988**, 38, 3098-3100.

[6] Myśliborski, R.; Hurej, K.; Pawlicki, M.; Latos-Grażyński, L. Inversion Triggered by Protonation—A Rubyrin with Embedded α,β′-Pyridine Moieties. *Angewandte Chemie International Edition* **2018**, *57* (51), 16866-16870.

[7]Malakalapalli, R. R.; Mangalampalli, R. Tetrahedron 2012, 68, 1306−1314.

[8] Ahmad, S.; Yadav, K. K.; Singh, S. J.; Chauhan, S. M. S. Synthesis of 5,10,15,20 Meso-Unsubstituted and 5,10,15,20-Meso-Substituted-21,23-Ditellura/Diselena Core Modified Porphyrinogens: Oxidation and Detection of Mercury(II). RSC Adv. 2014, 4 (7), 3171–3180.
